# Proceedings of the IX International Symposium on Phlebotomine Sandflies (ISOPS IX), Reims, France, June 28th–July 1st, 2016

**DOI:** 10.1051/parasite/2016051

**Published:** 2016-09-27

**Authors:** Jérôme Depaquit, Bernard Pesson, Denis Augot, James Gordon Campbell Hamilton, Phillip Lawyer, Nicole Léger

**Affiliations:** 1 Université de Reims Champagne Ardenne, ANSES, SFR Cap santé, EA 4688 – USC « Transmission Vectorielle et Épidémiosurveillance de Maladies Parasitaires (VECPAR) » Reims France; 2 Infectious Disease Transmission and Biology Group, Department of Biomedical and Life Sciences, Faculty of Health and Medicine, Lancaster University Lancaster LA1 4YG UK; 3 Laboratory of Parasitic Diseases, National Institute of Allergy and Infectious Diseases, National Institutes of Health Bethesda Maryland USA


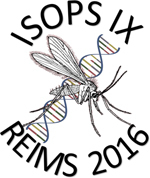


## Table of contents

### Inventories, ecology and ethology (oral communications)

**Phlebotomine sand fly fauna in the Federal District of Brazil**

*Aline Machado Rapello, Andrey José de Andrade, Douglas de Almeida Rocha, Jônatas C.B. Ferreira, Renata VelôsoTimbó, Marcos Takashi Obara, Rodrigo Gurgel Gonçalves*

**Identification of host preferences from sand flies captured in endemic leishmaniasis regions of Ecuador**

*Estefanía Palacios, Estefanía Espinosa, Gabriela Vela, Denis Augot, Jérôme Depaquit, Sonia Zapata*

**Diversity of phlebotomine sand fly assemblage in a tropical forest of southern Mexico**

*Jorge J. Rodríguez-Rojas, Eduardo A. Rebollar-Téllez*

**Phlebotomine sand flies (Diptera: Psychodidae) of Texas, United States**

*Stavana E. Strutz, Cory L. Schlesener, Ryan Baggerly, Jérôme Depaquit, Camille Parmesan*

**EU-ECDC/EFSA VectorNet Project: Distribution of sand fly species (Diptera: Psychodidae), community analysis and pathogen detection in Balkans**

*Vit Dvorak, Ozge Erisoz Kasap, Gizem Oguz, Nazli Ayhan, S. Vaselek, J. Omeragic, I. Pajovic, F. Martinkovic, O. Mikov, J. Stefanovska, D. Petric, D. Baymak, Yusuf Ozbel, Jérôme Depaquit, Vladimir Ivovic, Petr Volf, Bulent Alten*

**First data on sand fly presence in Slovenia**

*Alexandra Popovič, Eva Praprotnik, Vladimir Ivović*

**Distribution and travel distance of *Phlebotomus papatasi* (Diptera: Psychodidae) in a zoonotic cutaneous leishmaniasis focus, the Northern Negev, Israel**

*Laor Orshan, Shirly Elbaz, Yossi Ben-Ari*

**Phlebotomine sand flies (Diptera: Psychodidae) in Morocco: Results of entomological surveys in five regions of Morocco, Marrakech, Al Haouz-Immintanoute, Tlat Hanchan, and Had Dra**

*Mohamed Echchakery, Fouad Ouanaimi, Samia Boussaa, Ali Boumezzough*

**First Phlebotominae in Botswana**

*Andreas Krüger*

**Uncharted phlebotomine sand flies in Gabon**

*Nil Rahola, Judicael Obame, Boris Makanga, Diego Ayala, Jérôme Depaquit, Christophe Paupy*

### Inventories, ecology and ethology (posters)

**Ecological niche modelling of phlebotominae sand flies and the relation to the distribution of cutaneous leishmaniasis in an endemic region of South-Eastern Brazil**

*Blima Fux, Viviane Coutinho Meneguzzi, Claudiney Biral dos Santos, Carina Margonari Gustavo Rocha Leite, Aloísio Falqueto*

**First recording of *Sciopemyia vattierae* (Diptera: Psychodidae: Phlebotominae) in the State of Acre, Southeast of the Brazilian Amazon**

*Márcia Moreira de Ávila, Andreia Fernandes Brilhante, Eunice Aparecida Bianchi Galati, Reginaldo Peçanha Brazil*

**Sand fly fauna (Diptera: Psychodidae) in an endemic area of American cutaneous leishmaniasis of Brazilian Amazon**

*Márcia Moreira de Ávila, Andreia Fernandes Brilhante, Eunice Aparecida Bianchi Galati, Reginaldo Peçanha Brazil*

**Environmental factors and the occurrence of *Nyssomyia whitmani* and *Lutzomyia cruzi* in the Brazilian Central West**

*Wagner de Souza Fernandes, Anny Keli Aparecida Alves Cândido, Leandro Machado Borges, Aline Etelvina Casaril, Jucelei de Oliveira Moura Infran, Elisa Teruya Oshiro, Suellem Petilim Gomes, Antônio Conceição Paranhos Filho, Eliane de Mattos Piranda, Alessandra Gutierrez de Oliveira*

**Normalized Difference Vegetation Index (NDVI) in the characterization of sand flies environments**

*Helen Rezende de Figueiredo, Anny Keli Aparecida Alves Cândido, César Claudio Cáceres Encina, Everton Falcão de Oliveira, Jaíza Santos Motta, Jucelei de Oliveira Infran, Aline Etelvina Casaril, Elisa Teruya Oshiro, Antonio Conceição Paranhos Filho, Alessandra Gutierrez de Oliveira*

**Preliminary information on insular sand flies, in Paraná River, Brazil**

*Barbara Andreo dos Santos, Kárin Rosi Reinhold-Castro, Herintha Coeto Neitzke-Abreu, Edilson Colhera Cristóvão, Thaís Gomes Versignassi Silveira, Ueslei Teodoro*

**Entomological surveillance in *Leishmania* transmission areas on the border between Brazil and Bolivia**

*Elizabeth F. Rangel, Adriana Zwetsch, Antônio Luís F. Santana, Rodrigo E. Godoy, Júlia S. Silva, José O. Silva, Paulo S. Almeida, Zaida E. Yadon*

**Analysis of feeding preference of sand flies captured in peridomestic habitats in Panama**

*Ana Rosa Caballero, Anayansi Valderrama*

**Why sand fly samplings of a single night are insufficient? An analysis in an urban area of northeast Argentina based on light traps**

*Maria Soledad Santini, Maria Soledad Fernández, Maria Gabriela Quintana, Regino Cavia, Oscar Daniel Salomón*

**Identification of natural breeding sites of sand flies in an endemic zone of cutaneous leishmaniasis in Argentina**

*Mariana Manteca Acosta, Maria Soledad Santini, Maria Eugenia Utgés, Oscar Daniel Salomón*

**Update on Phlebotominae fauna from the Chaco region, Argentina**

*Enrique Alejandro Szelag, Jose Dilermando Andrade Filho, Juan Ramón Rosa, María Gabriela Quintana, Oscar Daniel Salomón*

**Phlebotominae: spatial-temporal distribution in Corrientes city, Argentina**

*Pablo E. Berrozpe, Maria Soledad Santini, A.V. Araujo, D. Lamattina, Oscar Daniel Salomon*

**Distribution, abundance, and genetic variability of *Lutzomyia longipalpis* (Diptera: Psychodidae) in Tartagal city, Salta, Argentina**

*María Gabriela Quintana, María Soledad Santini, Andrea Gómez Bravo, Ana Denise Fuenzalida, Mariana Manteca Acosta, Angélica Pech-May, Oscar Daniel Salomón*

**Updated distribution records of phlebotomine sand flies (Diptera: Psychodidae) of Spain**

*Javier Lucientes, Rosa Estrada, Vladimir Oropeza-Velasquez, Sarah Delacour-Estrella, Pedro María Alarcón-Elbal, José Ignacio Ruiz-Arrondo, Ricardo Molina*

**Presence of *Phlebotomus* (*Transphlebotomus*) *mascittii* Grassi, 1908, in northern Spain: first record for the Cantabrian Region and second for the Iberian Peninsula**

*Javier Lucientes, Rosa Estrada, Vladimir Oropeza-Velasquez, Sarah Delacour-Estrella, Pedro María Alarcón-Elbal, José Ignacio Ruiz-Arrondo, Ricardo Molina*

**Blood feeding behavior of *Phlebotomus perniciosus* collected in the human leishmaniasis focus of southwest Madrid, Spain, during the period 2012–2015**

*Estela González, Ricardo Molina, Ana Tello, Andrés Iriso, Ángeles Vázquez, Maribel Jiménez*

**Ecological aspects of phlebotomine sand flies in Gran Canaria (Canary Islands, Spain) and risk of *Leishmania* transmission**

*Francisco Morillas Márquez, Montserrat Gállego Culleré, M.J. Morillas Mancilla, V. Diaz Saez, G. Merino Espinosa, Bernard Pesson, C. Muñoz Batet, V. Corpas López, Joaquina Martín Sánchez*

**First study of phlebotomine sand flies (Diptera, Psychodidae), vectors of *Leishmania* sp., in Castelo Branco District, Central East region, Portugal**

*M.L. Vilela, Daniela De Pita-Pereira, Thais de Araujo-Pereira, J.M. Cristovão, Carla Maia, Leana Campino, M. Magalhães, Elisabeth F. Rangel, Maria Odete Afonso*

**Phlebotomine sand fly species distribution in Croatia and implications in *Leishmania* transmission**

*Sanja Bosnić, Gioia Bongiorno, Cristina Khoury, Trentina Di Muccio, Luigi Gradoni, Marina Gramiccia, Michele Maroli*

**Sand flies (Diptera: Psychodidae) of Mediterranean Africa: Inventory and distribution**

*Adel Rhim, Youmna M’Ghirbi, Jacques Brunhes, Ali Bouattour*

**An entomological survey for sand flies in two counties of Taiwan**

*Chizu Sanjoba, Yusuf Ozbel, Jiamei Sun, Mehmet Karakus, Kwang-Poo Chang, Chi-Wei Tsai, Tai-Chuan Wang, Yoshitsugu Matsumoto*

**An inventory of phlebotomine sand flies from Cambodia**

*Thibault Vallecillo, Eva Krupa, Julian Gratiaux, Idiyana Rahima Abdou el Aziz, Kimsour Kang, Kalian Ouk, Mathieu Loyer, Frédérick Gay, Arezki Izri, Jérôme Depaquit*

### Laboratory studies (oral communications)

**Keynote – The unparalleled efficiency of *Leishmania* transmission by sand fly bites**

*Ranadhir Dey, Vanessa Atayde, Amritanshu Joshi, Hamide Aslan, Lais da Silva, Shannon Townsend, Claudio Meneses, Hira Nakhasi, Martin Olivier, Jesus Valenzuela, Shaden Kamhawi*

**A comparison of vector competence in different sand fly species to transmit *Leishmania donovani***

*Jovana Sadlova, Jitka Myskova, Katerina Pruzinova, K. Homola, M. Yeo, Petr Volf*

***Leishmania donovani* in *Phlebotomus argentipes*: comparison of development and transmission of amastigote- and promastigote-initiated infections**

*Tereza Lestinova, Jovana Sadlova, Jitka Myskova, Jan Votypka, V. Yeo, Petr Volf*

**Establishing, expanding and certifying a closed working colony of *Phlebotomus argentipes* (Diptera: Psychodidae) for xenodiagnostic studies at the kala azar medical research center, Muzaffarpur, Bihar, India**

*Puja Tiwary, Shakti Kumar Singh, O.P. Singh, David Sacks, Shyam Sundar, Edgar Rowton, Phillip Lawyer*

***Leishmania tropica* development in *Phlebotomus sergenti*: The effect of temperature, gregarines and geographic origin of sand flies**

*Jana Hlavacova, Magdalena Jancarova, Jan Votypka, Petr Volf*

*Lutzomyia umbratilis* population captured in the south of the Negro River is refractory to interaction with *Leishmania guyanensis*

*R.P. Soares, P.M. Nogueira, N.F.C. Secundino, E.F. Santos, C.M. Ríos-Velásquez, F.A. Pessoa*

*Lutzomyia migonei* is a permissive vector competent for *Leishmania infantum*

*Katerina Pruzinova, Vanessa Cristina Fitipaldi Veloso Guimarães, Jovana Sadlova, Vera Volfova, Sinval Pinto Brandão Filho, Petr Volf*

***Leishmania* proteophosphoglycans regurgitated from infected sand flies accelerates dermal wound repair and exacerbates leishmaniasis via insulin-like growth factor 1-dependent signalling**

*Emilie Giraud, Tamsyn Derrick, Oihane Martin, Rod J. Dillon, Tereza Leštinová, Petr Volf, Ingrid Műller, Paul A. Bates, Matthew E. Rogers*

**Attraction of *Lutzomyia* sp. (Diptera: Psychodidae: phlebotomine) to volatile organic compounds from the skin odour of individuals residing in an endemic area for tegumentary leishmaniasis**

*D.S. Tavares, P.R.R. Mesquita, V.R. Salgado, F.M. Rodrigues, J.C. Miranda, A. Barral*

**Blood derived haem as a potential elicitor of anti-leishmanial activity in the gut of the female sand fly *Lutzomyia longipalpis***

*José R. Silva, Emma Shawcross, Rod J. Dillon*

**Bacterial communities associated with the digestive tract of wild populations of *Lutzomyia evansi*: a vector of *Leishmania* in Colombia**

*Rafael José Vivero, Gloria Ester Cadavid-Restrepo, Sandra I. Uribe Soto, Claudia Ximena Moreno Herrera, Ivan D. Velez*

### Systematics and phylogeny (posters)

**What we know of the classification, evolution, and dispersion of *Leishmania* parasites and sand flies?**

*Mohammad Akhoundi, Katrin Kuhls, Arnaud Cannet, Jan Votýpka, Pierre Marty, Pascal Delaunay, Denis Sereno*

**Illustrated identification key to females of Phlebotominae recorded in the Central-West Region of Brazil using only head and spermathecae**

*Douglas de Almeida Rocha, Eunice Aparecida Bianchi Galati, Andrey José de Andrade*

**First record of *Psychodopygus francoisleponti* Zapata, Depaquit & Léon 2012 (Diptera: Psychodidae) in Acre State, Brazil**

*Andreia Fernandes Brilhante, Márcia Moreira de Ávila, Rodrigo Espíndola Godoy, Jailson Ferreira de Souza, Cristiane de Oliveira Cardoso, Eunice Aparecida Bianchi Galati*

**Morphological and morphometric characters to distinguish females of three sympatric species of the genus *Trichophoromyia* (Diptera: Psychodidae: Phlebotominae) in a Brazilian Amazonian area**

*Andreia Fernandes Brilhante, Priscila Bassan Sábio, Eunice Aparecida Bianchi Galati*

**Metaphase karyotyping organization of *Lutzomyia cruzi* – preliminary result**

*Mirella Ferreira da Cunha Santos, Natália Camargo Braga, Douglas Araújo, Lucas Osti de Freitas, Wagner Fernandes, Elisa Teruya Oshiro, Alessandra Gutierrez de Oliveira*

**Phylogeography and genetic variability of populations of *Lutzomyia longipalpis* (Diptera: Psychodidae) inferred from ND4 gene**

*Angélica Pech-May, Janine Ramsey, Domingo Liotta, Magali Giuliani, Pablo Berrozpe, María Gabriela Quintana, Oscar Daniel Salomón*

**It is time to use a non destructive method for DNA extraction from phlebotomine sand flies**

*Julian Gratiaux, Eva Krupa, Thibault Valecillo, Denis Augot, Véronique Lehrter, Jean-Charles Gantier, Jean-Yves Rasplus, Jérôme Depaquit*

### Systematics and phylogeny (oral communications)

**Keynote – Fossil contribution in the classification of Psychodidae**

*Dany Azar*

**Geometric and linear morphometry as a tool for discriminating cryptic female specimens of *Psychodopygus* genus Chagasi series**

*Rodrigo Espíndola Godoy, Elizabeth Ferreira Rangel, Eunice Aparecida Bianchi Galati*

**Lutzodex^TM^ – a digital key for sand flies (Diptera: Phlebotominae) using Android App**

*Douglas de Almeida Rocha, Maxwell Ramos de Almeida, Andrey José de Andrade*

***Phlebotomus* (*Paraphlebotomus*) *chabaudi* Croset, Abonnenc & Rioux, 1970 and *Phlebotomus riouxi* Depaquit, Killick-Kendrick & Léger, 1998: synonyms or closely related species?**

*Véronique Lehrter, Jérôme Depaquit*

**Beware of *Sergentomyia* from Southeastern Asia due to untimely synonymies and a need to describe new species**

*Jérôme Depaquit*

### Epidemiology, laboratory studies & modern tools (posters)

**The aminosugar galactosamine reduces the trypsinolytic activity of *Lutzomyia longipalpis* (Diptera: Psychodidae) and promotes *Leishmania mexicana* and *Leishmania infantum* development within the sand fly gut**

*T. Lima-Silva, L.K. Castro, A. Bortolini, Marcos H. Pereira, R.N. Araújo, N.F. Gontijo, Mauricio R.V. Sant’ Anna*

**Evaluation of different diets for feeding larvae of *Nyssomyia neivai* (Diptera: Psychodidae: Phlebotominae)**

*Antonio Carlos Ferrari Júnior, Kleiton Maciel dos Santos, Magda Freitas Fernandes, Wedson Desidério Fernandes, Herintha Coeto Neitzke-Abreu, Maria Elizabeth Moraes Cavalheiros Dorval, Alessandra Gutierrez de Oliveira, Eunice Aparecida Bianchi Galati*

**Is there oviposition pheromone in *Nyssomyia neivai* (Diptera: Psychodidae)?**

*Thais Marchi Goulart, Camila Feitosa de Castro, Wanderson Henrique Cruz Oliveira, Flávia Benini da Rocha Silva, Vicente Estevam Machado, Dennys Ghenry Samillan Ortiz, Christiann Davis Tosta, Mara Cristina Pinto*

**Experimental infection of *Phlebotomus perniciosus* by bioluminescent *Leishmania infantum* using a murine model and artificial feeder**

*Arnaud Cannet, Mohammad Akhoundi, Michel Gregory, Pierre Marty, Pascal Delaunay*

**Exploring the migration of kinetoplastid parasites in sand flies; why are hypopylarian parasites backward in coming forward?**

*Raquel J. Vionette-Amaral, C.T. Nogueira, M. Ginger, Rod J. Dillon*

**Molecular and serological methods for evaluating blood meal sources in phlebotomines sand flies (Diptera: Psychodidae)**

*Mauricio Baum, Edilene Alcântara de Castro, Elias Seixas Lorosa, Mara Cristina Pinto, Thais Marchi Goulart, Walter Baura Magda Clara Vieira da Costa-Ribeiro*

**Host feeding preference and molecular screening of *Leishmania* infection in wild-caught sand flies in an endemic focus Aydın, Turkey**

*Mehmet Karakuş, Metin Pekağırbaş, Samiye Demir, Hasan Eren, Seray Töz, Yusuf Özbel*

**Anthropophilic behaviour and detection of *Leishmania* spp. in *Sergentomyia minuta* collected in the human leishmaniasis focus of Madrid, Spain**

*Estela González, Ana Tello, Ricardo Molina, Andrés Iriso, Ángeles Vázquez, Maribel Jiménez*

**Molecular detection of *Leishmania tropica* parasites kDNA from naturally infected sand flies in a new foothill endemic area, southeast Iran**

*M.D. Moemenbellah-Fard, K. Azizi, M.R. Fakoorziba, T. Dabaghmanesh, M. Ahmadyousefi-Sarhadi*

**Epidemiology of cutaneous leishmaniasis in the municipality of Brasiléia, Acre State: Study on the sandy fly fauna**

*Thais De Araujo-Pereira, Daniela De Pita-Pereira, Mariana Boité, Daniella Alves Martins, Taina A.N. Da Costa-Rego, Israel De Souza Pinto, Regina Barbosa Moreira, Andressa A. Fuzari, José Dilermano Andrade-Filho, Marcia Oliveira, Reginaldo Brazil, Constança Britto*

**Seasonal dynamics, evolution of *Leishmania infantum* infection rates, and host-feeding preferences of *Phlebotomus perniciosus* in the focus of human leishmaniasis in the Madrid region, Spain (2012–2014)**

*Ricardo Molina, Estela González, Sonia Hernández, Inés Martín-Martín, Maribel Jiménez*

**Molecular tools for the identification of phlebotomine sand flies and detection of *Leishmania* spp. parasites in Misiones province, Argentina**

*Sofía L. Moya, Magalí G. Giuliani, Mariana Manteca Acosta, Oscar D. Salomón, Domingo J. Liotta*

### Epidemiology and control (oral communications)

**Keynote – Can *Sergentomyia* spp. play a role in the transmission of human and animal leishmaniases?**

*Carla Maia*

**Molecular analysis of parasite, vector and blood meal DNA from field-caught sand flies in a Moroccan focus of cutaneous leishmaniasis: Genetically heterogenous *Leishmania tropica* in *Phlebotomus sergenti* as a mono-specific and multi-host feeding vector**

*Malika Ajaoud, Nargys Es-Sette, Rémi N. Charrel, Abderahmane Laamrani-Idrissi, Myriam Riyad, Meryem Lemrani*

**Sand flies abundance, ecology and oviposition preferences in Bihar, India**

*Rajesh B. Garlapati, Shanta Mukherjee, Rahul Chaubey, Tahfizur Rahaman, Piyoosh Babele, Akanksha Chowdhury, Suman Prakash, Vinod Kumar, Mukesh Kumar, Gregory Franckowiak, Dan Somers, Lindsay Briley, Katelyn Wagner, Jenna Hulke, McCall Calvert, Larisa Polyakova, David Poche, Richard Poche*

**Keynote – Phlebotomine flies vectors of arbovirus: review and recent data**

*Rémi N. Charrel*

**Sand fly fever in Iran: from the past up to the isolation of Dashli virus (a new Sicilian like virus)**

*Vahideh Moin-Vaziri, Cigdem Alkan, M. Badakhshan, N. Rahbarian, Xavier de Lamballerie, Rémi N. Charrel*

**Sand fly fauna of Palmas, state of Tocantins, Brazil: occurrence in different environments and natural infection by trypanosomatids**

*Tâmara Dias Oliveira Machado, Tauana de Sousa Ferreira, Alcinei de Souza Santos Junior, Nathyla Morgana Cunha Sales, Renata Velôzo Timbó, Tamires Emanuele Vital, Thaís Tâmara Castro Minuzzi-Sousa, Andrey José de Andrade, Marcos Takashi Obara, Rodrigo Gurgel-Gonçalves*

**First detection of an unknown *Trypanosoma* DNA in a phlebotomine sand fly collected from southern Thailand**

*Atchara Phumee, Apiwat Tawatsin, Usavadee Thavara, Theerakamol Pengsakul, Suwich Thammapalo, Jérôme Depaquit, Frédérick Gay, Padet Siriyasatien*

**Overview and an update of the current knowledge and perspectives on sand fly research in Mexico**

*Eduardo A. Rebollar-Téllez, Sergio I. Ibáñez-Bernal, Jorge J. Rodríguez-Rojas, David A. Moo-Llanes, Angélica Pech-May, Ana C. Montes de Oca-Aguilar, Oscar Mikeri-Pacheco, Miriam Berzunza-Cruz, Ingeborg Becker-Fauser, Janine Ramsey, Carlos Ibarra-Cerdeña, Ángel Rodríguez-Moreno, Christopher Stephens, Victor Sánchez-Cordero, Alfredo Castillo-Vera, Camila González, Wilfredo Arque-Chunga, Javier Escobedo-Ortegón, Silvia Pasos-Pinto, Laura Sánchez-García*

**Abundance of *Lutzomyia longipalpis* (Diptera, Psychodidae) in a kennel and its surroundings on a highly endemic visceral leishmaniosis area in São Paulo State, Brazil**

*Andre A. Cutolo, K.B.S. Briguente, G. Motoie, C.E.J. Pigozzi, B.L. Neves, I. Menz, V.L. Pereira-Chioccola*

**Canine visceral leishmaniasis in the São Paulo metropotian area dissociated of *Lutzomyia longipalpis: Pintomyia fischeri* as potential vector of *Leishmania infantum chagasi***

*Fredy Galvis Ovallos, Eunice A.B. Galati*

**The emergence and spread of leishmaniases in the borders of Argentina, Brazil, Paraguay and Uruguay**

*Oscar Daniel Salomón, María Gabriela Quintana, María Soledad Santini, Nilsa González-Britez, Nidia Martínez, Antonieta Rojas de Arias, Vanete Thomaz-Soccol, André Luiz Gonçalves, Alceu Bisetto Júnior, Gabriela Willat, Luis Calegari, Yester Basmadjian, Zaida E. Yadon, and the IDRC Project #107577 team*

**Evaluation of the synthetic sex pheromone, (*S*)-9-methylgermacrene-B, for recruitment and monitoring of *Lutzomyia longipalpis* (Diptera: Psychodidae) in an environmental reserve in Rio de Janeiro, Brazil**

*Vanessa De Araujo Barbosa, Andressa Alencastre Fuzari Rodrigues, James Gordon Campbell Hamilton, Reginaldo Peçanha Brazil*

**Synthetic pheromone and long lasting insecticidal nets (LLINs) as a new control strategy for *Lutzomyia longipalpis* (Diptera: Psychodidae), the vector of *Leishmania* (*Leishmania*) *infantum***

*Vanessa De Araujo Barbosa, Cristian Ferreira De Souza, James Gordon Campbell Hamilton, Reginaldo Peçanha Brazil*

**Identifying the Yeast community in the sand fly *Phlebotomus perniciosus:* towards a strategy for yeast-mediated biological control of vector-borne diseases**

*Elena Martin, Ilaria Varotto Boccazzi, Gioia Bongiorno, Leone De Marco, Luigi Gradoni, Nicoletta Basilico, Stefano Comazzi, Irene Ricci, Sara Epis*

**Targeting sand fly control by the use of systemic insecticides presented to mammalian reservoir hosts of ZCL and VL: A review of recent studies**

*Richard M. Poché, Daniel Hartman, Larisa Polyakova, Rajesh Babu Garlapati, David Poché*

**Systemic insecticides used in dogs: potential candidates for sand fly control?**

*Sonia Ares Gomez, Albert Picado*

**Repellent efficacy of a new combination of fipronil and permethrin against the main vector of canine visceral leishmaniasis in the Americas (*Lutzomyia longipalpis*)**

*Andre A. Cutolo, Fredy Galvis Ovallos, E.S. Neves, S. Sossai, M.M.F. Vieira, F.O. Silva, S.T. Chester, B. Fankhauser, M.D. Soll*

**Molecular and biochemical characterization of insecticide resistance in *Phlebotomus* and *Lutzomyia* sand flies**

*Scott A. Bernhardt, David S. Denlinger, Zachariah Gompert, Joseph S. Creswell*

**Evaluation of the spatial relationship between area of insecticide treatment and location of Leishmaniasis cases using geographical information systems in Adana, Turkey**

*Hakan Kavur, Ozan Artun, Kenan Koca*

**Manipulation of sand fly distributions within the peridomestic environment, and implications for the control of vector borne disease**

*Erin Dilger, Graziella Borges-Alves, Vicky Carter, M.G. Herededia, C.M. Nunes, L.M. Garcez, Reginaldo Peçanha Brazil, James Gordon C. Hamilton, Orin Courtenay*

**KalaCORE research on the efficacy of control measures against *Phlebotomus orientalis*, the principal vector of Visceral Leishmaniasis in East Africa**

*Dia-Eldin Elnaiem, Omran F. Osman, Wossenseged Lemma, Hanan A.A. Elhadi, Bakri Y.M. Nour, Noteila M. Khalid, Mulat Yimer, Jorgi Alvar, Orin Courtenay*

**Visceral Leishmaniasis on the Indian Subcontinent: modelling the dynamic relationship between vector control schemes and vector life cycles**

*David M. Poché, William E. Grant, Hsiao-Hsuan Wang*

### Epidemiology and control (posters)

**Dynamics of *Laroussius* populations and *Leishmania* infection rate of female sand flies in an endemic visceral leishmaniasis region, Tunisia, North Africa**

*Meriem Benabid, Adel Rhim, Rania Ben Romdhane, Manel Zerzri, Aïda Bouratbine*

**Epidemiologic survey of phlebotomine vectors in a canine leishmaniasis endemic area in Spain**

*Rita Velez, C. Ballart, E. Domenech, J. Cairó, Montserrat Portús, Montserrat Gállego*

**Evidence for stable endemic sand fly populations in the light of migration streams into Austria**

*Adelheid G. Obwaller, Mehmet Karakus, Wolfgang Poeppl, Seray Toz, Yusuf Ozbel, Horst Aspöck, Julia Walochnik*

**Absence of *Leishmania*-infected phlebotomines in gallery forests of the Federal District of Brazil**

*Aline Machado Rapello, Thaís Tâmara Castro Minuzzi-Sousa, Tamires Emanuele Vital, Tauana Ferreira, Renata Velôzo Timbó, Andrey José de Andrade, Rodrigo Gurgel Gonçalves*

**Vectors of the subgenus *Leishmania* (*Viannia*) in the Tapajós national forest reserve located in the lower Amazon Region of Brazil**

*Adelson Alcimar de Souza, Thiago Vasconcelos dos Santos, Yara Lins Jennings, Edna Aoba Ishikawa, Iorlando Barata, Maria das Graças Silva, José Aprígio Lima, Jeffrey Shaw, Ralph Lainson, Fernando Silveira*

**Natural transovarial and transstadial transmission of *Leishmania infantum* in *Rhipicephalus sanguineus* (Acari: Ixodidae)**

*Kourosh Azizi, Qasem Asgari, Mohammad Djaefar Moemenbellah-Fard, Aboozar Soltani, Tahereh Dabaghmanesh*

**Molecular epidemiology of phlebovirus in four provinces in Morocco**

*Nargys Es-Sette, Malika. Ajaoud, Rémi N. Charrel, Meryem Lemrani*

**Phleboviruses circulating in sand flies in Emilia-Romagna region (Northern Italy) in 2013–2015**

*Mattia Calzolari, Romeo Bellini, Paolo Bonilauri, Marco Pinna, Francesco Defilippo, Michele Dottori, Paola Angelini*

**Isolation of Piura virus, an insect-specific negevirus, from *Lutzomyia evansi* in Colombia**

*María Angélica Contreras-Gutiérrez, Hilda Guzman, Marcio R.T. Nunes, Sandra Uribe, Rafael Vivero, Iván Darío Vélez, Nikos Vasilaskis, Robert B. Tesh*

**Characterization of susceptibility of Phlebotominae (Diptera: Psychodidae) to the insecticide, alpha-cypermethrin**

*Douglas de Almeida Rocha, Andrey José de Andrade, Luciana Moura Reinaldo, Marcos Takashi Obara*

**Evaluation of the level of knowledge of public health professionals regarding the vector of visceral leishmaniasis and its control measures**

*Anna Ariel Polegato Martins, Mariana Fuga, Alessandra Gutierrez de Oliveira, Mirella Ferreira da Cunha Santos*

**Comparison of various recombinant salivary proteins as epidemiological markers for dog exposure to *Phlebotomus perniciosus* in different localities in Italy, Portugal and Spain**

*Laura Willen, Tatiana Kostalova, Nikola Polanska, Tereza Lestinova, Carla Maia, Petra Sumova, Michaela Vlkova, Eleonora Fiorentino, Aldo Scalone, Gaetano Oliva, Fabrizia Veronesi, José Manuel Cristóvão, Orin Courtenay, Lenea Campino, Luigi Gradoni, Marina Gramiccia, Cristina Ballart, Montserrat Gállego, Petr Volf*

**Can we identify *Leishmania* super-spreaders to reduce transmission to sand fly vectors?**

*Aurore Lison, Steve Reed, Orin Courtenay*

**Vector control using long-lasting insecticidal nets against kala-azar in Bangladesh**

*Chizu Sanjoba, Yusuf Ozbel, Bunpei Tojo, Eisei Noiri, Yoshitsugu Matsumoto*

**Analysis of gene expression in a *Lutzomyia longipalpis*-derived cell line**

*Luzia M.C. Cortes, Barbara C.A. Melo, Franklin Souza-Silva, Bernardo A.S. Pereira, Felio J. Bello, Otacilio C. Moreira, Daniela de Pita-Pereira, Constança Britto, Carlos R. Alves*

### Modern tools for sand flies studies (oral communications)

***Leishmania* HASP and SHERP genes are required for *in vivo* differentiation, parasite transmission and host virulence attenuation**

*Johannes S.P. Doehl, Jovana Sádlová, Hamide Aslan, Sonia Metangmo, Jan Votýpka, Shaden Kamhawi, Petr Volf, Deborah F. Smith*

**A glance at what *Leishmania infantum chagasi* expresses inside *Lutzomyia longipalpis***

*Erich Loza Telleria, Thais Lemos da Silva, João Ramalho Ortigão Farias, Yara Maria Traub-Csekö*

***Lutzomyia longipalpis* TGF-β has a role in *Leishmania infantum chagasi* survival in the vector**

*Tatiana Di-Blasi, E. Loza-Telleria, C. Marques, R. Macedo-Couto, M. Neves, A.J. Tempone, M. Ramalho-Ortigão, Yara Maria Traub-Csekö*

**Novel method to quantify *Leishmania* metacyclic promastigotes delivered by individual sand fly bite reveals the efficiency of parasite transmission**

*Émilie Giraud, Oihane Martin, Matthew Rogers*

**Blood feeding effect on *Phlebotomus papatasi* SP15 and SP44 salivary transcripts**

*Nasibeh Hosseini-Vasoukolaei, Amir Ahmad Akhavan, Mahmood Jeddi-Tehrani, Farah Idali, Ali Khamesipour, Mohammad Reza Yaghoobi-Ershadi, Shaden Kamhawi, Jesus G. Valenzuela*

***Phlebotomus orientalis* salivary proteins and antigens**

*Iva Rohousova, Alon Warburg, Petr Volf*

**Parity/nulliparity and sand fly salivary gland-gene expression**

*Nasibeh Hosseini-Vasoukolaei, Amir Ahmad Akhavan, Mahmood Jeddi-Tehrani, Farah Idali, Ali Khamesipour, Mohammad Reza Yaghoobi-Ershadi, Shaden Kamhawi, Jesus G. Valenzuela*

**Different approaches for further application of ALDI-TOF mass spectrometry for species identification of phlebotomine sand flies**

*Kristýna Hlavackova, Vit Dvorak, Petr Halada, Petr Volf*

**MALDI-TOF protein profiling as a method of choice for high-throughput species identification of sand flies – an example from the Balkan**

*Vit Dvorak, Kristýna Hlavackova, Petr Halada, Bulent Alten, Vladimir Ivovic, J. Omeragic, I. Pajovic, F. Martinkovic, O. Mikov, J. Stefanovska, Petr Volf*

**New generation sequencing (NGS) as a tool for identification of pooled sand flies**

*Nazli Ayhan, Vit Dvorak, Cigdem Alkan, Petr Volf, Rémi N. Charrel*

## Inventories, ecology and ethology (oral communications)

**Phlebotomine sand fly fauna in the Federal District of Brazil**

Aline Machado Rapello^1^, Andrey José de Andrade^1,2^, Douglas de Almeida Rocha^1^, Jônatas C.B. Ferreira^1^, Renata VelôsoTimbó^1^, Marcos Takashi Obara^1^, Rodrigo Gurgel Gonçalves^1^

^1^Laboratório de Parasitologia Médica e Biologia de Vetores, Área de Patologia, Faculdade de Medicina, Universidade de Brasília, Brasil

^2^Laboratório de Parasitologia Molecular, Departamento de Patologia Básica, Universidade Federal do Paraná, Brasil

aline_rapello@hotmail.com

The Federal District (FD) is located in the Midwest Region (MR) of Brazil, which includes the States of Goiás, Mato Grosso and Mato Grosso do Sul. The FD has the lowest phlebotomine species richness of the MR (*n =* 29), which corresponds to 11% of the 273 registered species in Brazil. Some of these species such as *Lutzomyia longipalpis*, *Nyssomyia whitmani* and *Bichromomyia flaviscutellata* have been incriminated as important vectors of *Leishmania* species*.* This study updated the list of phlebotomine species in the FD by sampling sand flies in gallery forests in the area. Sand flies were captured in four areas, Água Limpa Farm (FAL), Biological Reserve of Contagem (REBIO), Brasilia’s National Park (PNB) and Botanic Garden of Brasília (JBB), in May and September, 2014. The entire capture effort entailed 1,280 HP-light trap nights and 16 Shannon trap sessions. A total of 1,209 sand flies were captured and 18 species were identified. The overall capture success was 18%, being higher in FAL (27%) and in September (20%). Most sand flies were captured in REBIO (*n* = 664) and in FAL (*n* = 472). In PNB, 64 sand flies were captured and nine were captured in JBB. The most captured species was *Bi. flaviscutellata* (*n* = 668), followed by *Psathyromyia pradobarrientosi* (*n* = 285). *Nyssomyia whitmani* (*Leishmania brasiliensis* vector) and *Bi. flaviscutellata* (*L. amazonensis* vector) were found in three gallery forests sampled. Even with the great capture effort, *Lu. longipalpis* was not captured, indicating that this species might be restricted to domiciliary areas in FD. *Brumptomyia guimaraesi*, *Br. brumpti*, *Micropygomyia ferreirana*, *Pa. pradobarrientosi*, *Pa. campograndensis* and *Evandromyia bourrouli* were reported for the first time in FD, expanding the known geographical distributions of these sand flies in Brazil. *Pa. pradobarrientosi* is reported for the first time in Brazil. Now, 35 species are registered in FD. phlebotomine species richness in FD is relevant when it is compared to Goiás (47 species), a state 59 times bigger than FD (area ~ 5.780 Km^2^) or even when compared to France, where there are six species registered and whose territory is about 111 times larger than FD.

**Identification of host preferences from sand flies captured in endemic leishmaniasis regions of Ecuador**

Estefanía Palacios^1^, Estefanía Espinosa^1^, Gabriela Vela^1^, Denis Augot^2^, Jérôme Depaquit^2^, Sonia Zapata^1^

^1^Instituto de Microbiología, Universidad San Francisco de Quito, Ecuador

^2^Université de Reims Champagne-Ardenne, ANSES, EA4688 – USC « Transmission Vectorielle et Épidémiosurveillance de Maladies Parasitaires (VECPAR) », Reims, France

szapata@usfq.edu.ec

Leishmaniasis is endemic in 22 of 24 Ecuadorian provinces. The disease is only recorded under its tegumentary form (cutaneous and mucocutaneous). Some wild animals play a crucial role in parasite transmission. However, a few reservoirs only have been identified in the past. Entomological collections were performed between 2012 and 2015 in five provinces of the northern part of the country where leishmaniasis is endemic (Bolívar, Esmeraldas, Orellana, Manabí and Pichincha). A total of 3,103 specimens were collected, of which 7.7% were engorged females. Ten species were collected in total including known *Leishmania* vectors. Amplification of the PNOC nuclear gene was performed to identify the source of blood meals from 50 specimens. The host preferences of sandflies are: *Choloepus hoffmanii*, *Choloepus hoffmani*, *Potos flavus*, *Bos taurus*, *Pecari tajacu*, *Capra hircus*, *Equus caballus*, *Cebus capucinus*, *Tapirus terrestris* and *Homo sapiens.* Of these, the first two had already been reported as reservoirs, while *Cebus capucinus* is suspected to be a reservoir of *Leishmania*. Moreover, we found anthropophylic behavior of two sand flies species in the Amazonia basin which are not related with *Leishmania* transmission. This study contributes to the understanding of the transmission cycle of *Leishmania* and identifying potential new sites of transmission due to the presence of vectors, reservoirs and humans.

**Diversity of phlebotomine sand fly assemblage in a tropical forest of southern Mexico**

Jorge J. Rodríguez-Rojas^1^, Eduardo A. Rebollar-Téllez^1,2^

^1^Universidad Autónoma de Nuevo León, Facultad de Ciencias Biológicas, Departamento de Zoología de Invertebrados, Laboratorio de Entomología Médica, Avenida Universidad S/N, Ciudad Universitaria, 66451 San Nicolás de los Garza, Nuevo León, México

^2^Universidad Autónoma de Nuevo León, Centro de Investigación en Ciencias de la Salud. Avenida Carlos Canseco S/N, Colonia Mitras Centro, 64460 Monterrey, Nuevo León, México

jorge.rodriguezrj@uanl.edu.mx; eduardo.rebollart@uanl.edu.mx

Knowledge of biotic components of a particular place must be understood in its true dimension, so the report of the species present in a given community is of fundamental value in terms of biodiversity. The main aim of this study was to estimate the diversity of phlebotomine sand flies in an endemic focus of cutaneous leishmaniasis in southern Mexico. Field work was carried out in a tropical forest (18°59′46″ N, 088°09′27″ W; 19 m above sea level) from August 2013 to July 2014. Sampling was conducted during three consecutive nights per month. In each trapping night, 48 traps were operated from 1800 to 2400 h. The traps were CDC light traps (incandescent and LEDs colors white, blue, red and green), as well as Disney traps, Shannon traps, Sticky traps and Delta traps. Collection of sand flies were carried out in four transects, using each transect as a randomized block design. Specimens were prepared for permanent slide mounting using Euparal^®^ and subsequent identification was accomplished using different morphological structures described in taxonomic papers. Measures of alpha community diversity were based on the quantification of the number of species (species richness) and the community structure as well as the dominance and evenness. To evaluate the number of species present in the area, we used the estimators Chao 2, Jacknife 2 and the equation of Clench. Heterogeneity was calculated with Shannon’s entropy index and true diversity. Dominance was evaluated by Simpson and Berger-Parker, and also evenness index and Margalef index were evaluated as well. With a total capture effort of 1,728 night-traps, 16,101 phlebotomine sand flies were collected, representing two genera and 13 species. The most abundant species were *Lu. cruciata* (Coquillett) (42.33%), *Lu. shannoni* (Dyar) (32.68%), *Brumptomyia mesai* (Sherlock) (9.75%) and *Lu. ovallesi* (Ortíz) (9.03%). Less abundant species were *Lu. carpenteri* (Fairchild and Hertig), *Lu. cayennensis maciasi* (Fairchild and Hertig), *Lu. cratifer* (Fairchild and Hertig), *Lu. deleoni* (Fairchild and Hertig), *Lu. olmeca olmeca* (Vargas and Díaz-Nájera), *Lu. permira* (Fairchild and Hertig), *Lu. steatopyga* (Fairchild and Hertig), *Lu. trinidadensis* (Newstead) and *Lu.* sp., which altogether accounted for only 5.66% of the total. Two species were observed (*Lu*. *permira* and *Lu*. sp) as “doubletons”. According to diversity estimates, 100% (Chao 2) and 85% (Jacknife2) of potential species in the study area were calculated. Species accumulation curves using Clench’s equation, presented a good fit to the predictive model (*a* = 4.47, *b* = 0.34, *r* ^2^ = 0.966, slope = 0.001) with 13 species, representing 100% of the species observed. Alpha diversity shows that Shannon entropy and true diversity were: H′ = 1.42 and ^1^D = 4.14 respectively, whereas the Simpson dominance index was *λ* = 0.31 and Berger-Parker *d* = 0.42, while equitativity index was *J* = 0.56 and Margalef index was *D*_Mg_ = 1.24. Four species of medical importance, namely *Lu*. *cruciata*, *Lu*. *shannoni*, *Lu*. *ovallesi* and *Lu*. *olmeca olmeca* were collected and represented 85.81% of the total. This inventory of phlebotomine sand flies is an important activity to enhance our knowledge of sand fly assemblages and guilds. The understanding of the population dynamics of sand flies could be an important factor for the implementation of strategies for epidemic control of these insect-borne diseases.

**Phlebotomine sand flies (Diptera: Psychodidae) of Texas, United States**

Stavana E. Strutz^1^, Cory L. Schlesener^1^, Ryan Baggerly^1^, Jérôme Depaquit^2^, Camille Parmesan^3^

^1^University of Texas at Austin, USA

^2^University of Reims Champagne-Ardenne, Reims, France

^3^Plymouth University, UK

stavana@utexas.edu

The phlebotomine sand fly fauna of Texas is poorly documented, which is somewhat surprising given that Texas has at least eight of the thirteen species known to occur within the United States and has active cutaneous leishmaniasis cases. The diversity of sand flies and the presence of leishmaniasis suggest that Texas is an especially important region to survey. Four of the species found in the United States may be potential vectors of leishmaniasis and three of these species have been documented within Texas. The recent spread of cutaneous leishmaniasis into northern portions of Texas and Oklahoma has increased the importance of documenting sand fly distributions. While it is important to document species’ ranges for biodiversity purposes, it is even more pressing now that human health risks have increased. Most surveys have been conducted in southern and central portions of Texas near historic cutaneous leishmaniasis foci. We surveyed 86 sites across the state and found at least four different genera (*Dampfomyia*, *Lutzomyia*, *Micropygomyia*, and *Psathyromyia*). Of these genera, seven species were tentatively identified with some species identification remaining ambiguous.

**EU-ECDC/EFSA VectorNet Project: Distribution of sand fly species (Diptera: Psychodidae), community analysis and pathogen detection in Balkans**

Vit Dvorak^1^, Ozge Erisoz Kasap^2^, Gizem Oguz^2^, Nazli Ayhan^3^, S. Vaselek^4^, J. Omeragic^5^, I. Pajovic^6^, F. Martinkovic^7^, O. Mikov^8^, J. Stefanovska^9^, D. Petric^4^, D. Baymak^10^, Yusuf Ozbel^11^, Jérôme Depaquit^12^, Vladimir Ivovic^13^, Petr Volf^4^, Bulent Alten^2,14^

^1^Charles University, Parasitology Department, Prague, Czech Republic

^2^Hacettepe University, Department of Biology, Ecology Division, Beytepe-Ankara, Turkey

^3^Aix Marseille University, Medical Faculty, Virology Laboratories, Marseille, France

^4^Novi Sad University, Faculty of Agriculture, Novi Sad, Serbia

^5^University of Sarajevo, Veterinary Faculty, Sarajevo, Bosnia & Herzegovina

^6^Podgorica University, Veterinary Faculty, Podgorica, Montenegro

^7^University of Zagrep, Veterinary Faculty, Zagrep, Croatia

^8^National Center for Infectious and Parasitic Diseases, Sofia, Bulgaria

^9^Ss. Cyril and Methodius University, Faculty of Veterinary Medicine, Skopje, Macedonia (FYROM)

^10^National Institute of Public Health, Pristina, Kosovo

^11^Ege University, Parasitology Department, Izmir, Turkey

^12^Université de Reims Champagne-Ardenne, Faculty of Pharmacy, ANSES, Reims, France

^13^Primorska University, Veterinary Faculty, Koper, Slovenia

^14^Hacettepe University, Institute of Science and Engineering, Beytepe, Ankara, Turkey

kaynas@hacettepe.edu.tr

VectorNet “A European network for sharing data on the geographic distribution of arthropod vectors, transmitting human and animal disease agents” project is supported by the EU-ECDC/EFSA consortium and coordinated by Avia-GIS, Belgium. This study shows some of the results and achievements of the sand fly-team efforts in eight Balkan countries in 2015 in the framework of the VectorNet Project. Eight countries (Bosnia & Herzegovina, Montenegro, Croatia, Bulgaria, Macedonia, Serbia, Kosovo, Slovenia), including 267 locations and 36 cities were studied by the sand fly team with the aim of determining the altitudinal and trans-sectional distribution of species, identifying species and detecting possible pathogens in the Balkans. Sand flies were collected with light traps for a total of 951 trap nights during the field missions. From this study, 12 species were identified and a total of 9,096 specimens collected from fieldwork. The results show that *Phlebotomus neglectus* (74%) is the dominant species in Balkan countries and this species was collected from all eight countries together with *Ph. tobbi* (10%). These two species comprise 84% of total sand fly abundance. Other species include: *Ph. perfiliewi s.l* (6.13%), *Sergentomyia minuta* (3.56%), *Ph. perniciosus* (1.57%), *Ph. papatasi* (1.35%), *Ph. simici* (0.9%), *Ph. mascitti* (0.45%), *Ph. sergenti* (0.1%), *Ph. alexandri* (0.07%), and *Ph. balcanicus* (0.03%) (found only in Montenegro). We also calculated some of the important community parameters such as similarity, richness, species diversity, species evenness and dominance for each country. The most similar countries in terms of species composition are B&H-Kosovo (0.77), Bulgaria-Kosovo (0.85), Serbia-Kosovo (0.86), Croatia-Macedonia (0.80) and Bulgaria-Macedonia (0.80). From these results, it appears that Macedonia and Serbia are ecotones (transition areas) in the Balkans. The highest species diversities were observed in Macedonia (1.366), Bulgaria (1.247) and Serbia (1.169), respectively. The lowest was B&H with 0.386 values. In contrast, B&H, like other less diverse countries, has the highest dominance value (81.4%). In pathogen detection studies, two novel viruses in B&H and Macedonia and *Leishmania infantum* parasites in B&H, Macedonia and Kosovo were detected. To the best of our knowledge, most of the information derived from this study was new to the Balkan countries.

**First data on sand fly presence in Slovenia**

Alexandra Popovič, Eva Praprotnik, Vladimir Ivović

Faculty of Mathematics, Natural Sciences and Information Technologies, University of Primorska, Slovenia

vladimir.ivovic@famnit.upr.si

Distribution of phlebotomine sand flies around Mediterranean basin is mostly well investigated and documented. Nevertheless, there are some regions where fauna of these, medically very important insects was studied many years ago or never. Slovenia is one of the smallest member countries of EU sited on the south of Austria and on the east of Italy but despite its small size it has very heterogeneous relief. The northern part of the country is composed of alpine and the southern part of Mediterranean and Karst landscape. Being the bridge between eastern and western part of the Northern Mediterranean, this region hosts unknown sand fly species and these are the first faunistic data. In order to evaluate Slovenia as a potential leishmaniasis endemic region we investigated presence of the disease vectors, particularly in the coastal part of the country. During the high season 2015, 565 specimens were collected and five species identified. *Ph. neglectus* and *Ph. perniciosus*, well known and proven vectors of *L. infantum* in the Mediterranean, were the most abundant (76% and 16% respectively). *Ph. papatasi* and *Ph. mascitti*, known and potential vectors of several phleboviruses and Leishmania parasites, were also present but in smaller numbers (3% and 4.8%). Medically not important species *Sergentomyia minuta* was also recorded (0.2%). During the study period the peak of sand fly abundance was in the beginning of July, gradually decreasing towards the end of August. It was expected to find all of recorded sand fly species and the biggest surprise was relatively high abundance of *Ph. mascitti* evenly present in all collection sites and always close to animal shelters.

**Distribution and travel distance of *Phlebotomus papatasi* (Diptera: Psychodidae) in a zoonotic cutaneous leishmaniasis focus, the Northern Negev, Israel**

Laor Orshan^1^, Shirly Elbaz^1^, Yossi Ben-Ari^2^

^1^Laboratory of Entomology, Ministry of Health, Jerusalem, Israel

^2^Israel Nature and Parks Authority, Jerusalem, Israel

Laor.Orshan@MOH.health.gov.iI

In recent years, endemic transmission of zoonotic cutaneous leishmaniasis (ZCL) has spread to new regions in Israel. In the southern part of the country, the new foci of ZCL caused by *Leishmania major* are located in the cultivated plains of the northwestern Negev. The agricultural pest *Meriones tristrami*, the main reservoir animal in this area, is very common as well as *Phlebotomus papatasi*, the only known vector species of *L. major* in Israel. A sand fly study was conducted in the summer of 2013 in and around a small cooperative community. The aim was to understand from where and how far away sand flies reach the residential area. Sand flies were collected from 55 sites in four categories of land use using CO_2_-baited modified CDC light traps. To study the flight distances sand flies were marked in the field by spraying the vegetation in five sites with sugar solutions containing different food dyes. The catch was counted, identified, *Leishmania* DNA was detected in pooled female samples and the presence of marked specimens was noted. Sand flies were abundant throughout the long summer, showing one seasonal peak in the warmest months August and September. *L. major* DNA was detected throughout the season except in June in 30/55 sites sampled. Infection rates increased towards the end of the season and the estimated risk of exposure was highest in September. Sand fly densities were low in the residential area and very high in the surrounding agricultural fields. The maximum dispersal distances were 1.91 km for females and 1.53 km for males. The maximum range recorded for females was limited by the distances between the marking sites and the most distant trap. The calculated mean distance traveled indicating the typical dispersal distances of the population was 0.75 km. The overall results indicated the existence of dense and mobile sand fly populations. In the agricultural fields there seemed to be numerous development sources and suitable resting sites for sand flies scattered over large areas. Sand flies apparently moved in all directions. Typically, *Leishmania-*infected *Ph. papatasi* females probably could reach the residential area from distances greater than 0.75 km.

**Phlebotomine sand flies (Diptera: Psychodidae) in Morocco: Results of entomological surveys in five regions of Morocco, Marrakech, Al Haouz-Immintanoute, Tlat Hanchan, and Had Dra**

Mohamed Echchakery^1^, Fouad Ouanaimi^1^, Samia Boussaa^1,2^, Ali Boumezzough^1^

^1^Équipe Écologie Animale et Environnement-Lab L2E (URAC 32), Université Cadi Ayyad, Faculté des Sciences Semlalia, Marrakech, Morocco

^2^I SPITS-Institut Supérieur des Professions Infirmières et des Techniques de Santé, Marrakech, Morocco

mohamedechchakery@gmail.com

In Morocco, 23 species have been described, which belong into two genera (14 of the genus *Phlebotomus* and nine of genus *Sergentomyia*). Only five species are involved in the transmission of leishmaniasis in Morocco: *Phlebotomus papatasi* vector of *Leishmania major*, *Ph. sergenti* vector of *L. tropica*; *Ph. ariasi* vector of *L. infantum* and *Ph. longicuspis*, *Ph. perniciosus*, potential vectors of *L. infantum*. Cases of cutaneous leishmaniasis (CL) due to *L. tropica* have gradually increased in the towns of Al Haouz, Chichaoua (Imintanoute), Essaouira (Tlat Hanchan-Had Dra) *tropica.* The objectives of this research were to inventory the phlebotomine sand flies species, and to identify the environmental factors that influence the abundance of species in different locations. Sand flies were captured with CDC light traps and sticky traps (castor-oil paper traps) placed at intradomicilary and peridomicilary sites for one night (18:00 h until 06:00 h) every 15 days, from May 2014 to June 2015. A total of 1,678 individuals were captured: 260 in Marrakech, 320 in Al Haouz, 380 in Imintanoute, 435 Tlat Hanchan and 283 in Had Dra. Relative abundance of species was as follows: In Marrakech, *Ph. papatasi* (40.3%), *Ph. sergenti* (17.7%), *Se. fallax* (12.9%), *Se. minuta* (22.6%) and *Ph. longicuspis* (6.5%). In Al Haouz, *Ph. papatasi* (26.90%), *Ph. sergenti* (46.36%), *Ph. perniciosus* (2.43%), *Ph. longicuspis* (4.3%), *Ph. alexandri* (1.3%), *Se. fallax* (9.3%), *Se. minuta* (6.2%), *Sergentomyia dreyfussi* (3.12%)*.* In Imintanoute, *Ph. sergenti* (56.40%), *Ph. longicuspis* (9.71%), *Ph. ariasi* (6.48%), *Ph. papatasi* (15.42%), *Ph. perniciosus* (0.85%), *Ph. alexandri* (2.36%), *Se. fallax* (2.12%), *Se. minuta* (6.30%), *Se. dreyfussi* (0.36%)*.* In Tlat Hanchan, *Ph. sergenti* (62.40%), *Ph. longicuspis* (12.60%), *Ph. alexandri* (4.54%), *Ph. perniciosus* (6.72%), *Ph. kazeruni* (0.26%), *Ph. langeroni* (0.36%), *Ph. bergeroti* (0.16%), *Se. minuta* (6.6%), *Se. antennata* (6.36%)*.* In Had Dra, *Ph. sergenti* (70.6%), *Ph. longicuspis* (11.6%), *Ph. alexandri* (5.6%), *Ph. perniciosus* (6.16%), *Ph. langeroni* (0.12%), *Ph. bergeroti* (0.13%), *Se. minuta* (7.6%), *Se. antennata* (5.22%). The abundance of species varied significantly. Sand fly population densities were highest in summer followed by fall, spring and winter. The eco-epidemiological scenarios in our endemic foci are associated with the presence of domestic mammals and poultry, sources of blood for sand flies which sustains the vector population and the risk of infection by *Leishmania*, The proximity of livestock manure, and cattle and sheep sheds to houses and Climatic conditions including rainfall, light, temperature, relative humidity, and air movement, are very important factors influencing the abundance of sand flies in endemic foci.

**First Phlebotominae in Botswana**

Andreas Krüger

Military Hospital Hamburg, Dept. Tropical Medicine, Hamburg, Germany, and Okavango Research Institute, Maun, Botswana

krueger@bnitm.de

Regarding the distribution of phlebotomine sandflies in the Afrotropical region Botswana (southern Africa) appeared as a blank spot on the map, although there are several reports from the surrounding countries, namely South Africa, Namibia, Zambia and Zimbabwe. The lack of data was probably due to the non-endemicity of leishmaniasis in this country. In a pilot vector survey with a CDC light trap, carried out during the wet season 2014–15 in Maun, northern Botswana, 41 sand fly specimens, belonging to four species, were detected: *Sergentomyia* (*Grassomyia*) *inermis* and *Se*. (*Sergentomyia*) “*bedfordi* group”. The latter comprised of specimens of *Se*. (*Ser*.) *congolensis*, *Se*. (*Ser*.) *caliginosa*, and *Se*. (*Ser*.) *salisburiensis*. None of these species are known vectors of human leishmaniasis parasites. Regarding the habitat, trap catches were all done beside a termite hill at a fan height of 50 cm, about 10–200 m off Thamalakane river. *Sergentomyia inermis* is a new record for the entire southern African fauna, and it remains to be confirmed whether older record of the closely related *Se. squamipleuris* from South Africa are correct. Molecular taxonomic analyses are underway to further characterize the taxa.

**Uncharted phlebotomine sand flies in Gabon**

Nil Rahola^1,2^, Judicael Obame^2^, Boris Makanga^2^, Diego Ayala^1,2^, Jérôme Depaquit^3^, Christophe Paupy^1^

^1^Unité MIVEGEC, UMR 224-5290 IRD-CNRS-UM, Centre IRD de Montpellier, BP 64501, 34394 Montpellier, France

^2^Centre International de Recherches Médicales de Franceville (CIRMF), BP 769, Franceville, Gabon

^3^Université de Reims Champagne Ardenne, ANSES, SFR Cap santé, EA 4688-USC « Transmission Vectorielle et Épidémiosurveillance de Maladies Parasitaires (VECPAR) », Reims, France

nil.rahola@ird.fr

Despite the former mention of an autochthonous case of visceral leishmaniasis, Gabon does not currently constitute an endemic country for this disease. As a result, the sand fly fauna in this country remains poorly documented and prospected. An exhaustive review of literature reports only four species in Gabon. Since 2012 we have managed to collect more than 10,000 sand flies through a large mosquito survey that consisted in 1850 CDC-miniature light traps installed in two different forest sites and 700 CDC-miniature light traps installed in two different caves. Over 2,000 specimens were collected in forest sites and 8,000 in caves. In addition, some collections were performed using other CDC-miniature light traps in anthropic environments such as small savannahs villages, forest villages and towns. These collections allowed us to expand the current checklist of phlebotomine sand flies of Gabon from four to 25 species, with already at least one species new to science (and two others being described). The forest had the highest species diversity and allowed us to discover and describe a new species of *Phlebotomus* with a quite singular morphology (*Phlebotomus* (*Legeromyia*) *multihamatus*) and also the unknown male of *Spelaeomyia moucheti*. This discovery led us to perform a molecular analysis of the whole genus *Spelaeomyia*. On the other hand, in caves, sand flies were very abundant but of low diversity. With these new collections we will be able to bring new morphological data, make some re-descriptions of some specimens such as the female of *Sa. moucheti* or *Sergentomyia lumsdeni*, but also to consider the possibility of the creation of a new subgenus of the genus *Sergentomyia*. This update of the phlebotomine sand flies of Gabon encourages further surveillance in this country. Even if only one case of leishmaniasis has been reported in Gabon, the role of sand flies in the transmission of such parasites or other infectious agents, as well as their trophic preferences, should be evaluated.

## Inventories, ecology and ethology (posters)

**Ecological niche modelling of Phlebotominae sand flies and the relation to the distribution of cutaneous leishmaniasis in an endemic region of South-Eastern Brazil**

Blima Fux^1^, Viviane Coutinho Meneguzzi^1^, Claudiney Biral dos Santos^2^, Carina Margonari^3^, Gustavo Rocha Leite^1^, Aloísio Falqueto^1^

^1^Unidade de Medicina Tropical da Universidade Federal do Espírito Santo. Av. Mal. Campos 1468, 29043-900, Vitória, ES, Brasil

^2^Núcleo de Entomologia da Secretaria da Saúde do Estado do Espírito Santo. Av. Mal. Campos 1468, 29043-900, Vitória, ES, Brasil

^3^Centro de Pesquisa Rene Rachou. Av. Augusto de Lima 1715, 30190-002, Belo Horizonte, MG, Brazil

blimafux@yahoo.com.br

Cutaneous leishmaniasis (CL) is caused by a protozoan of the genus *Leishmania*, which is transmitted by bites of phlebotomine sand flies. The State of Espírito Santo (ES), an endemic area in the Southeast of Brazil, has shown considerably high sand fly prevalence in recent decades, allowing the spread of the disease to unaffected areas. Computer tools, such as ecologic niche modelling (ENM), are useful for predicting potential disease risk. In this study, ENM was applied to species of sand flies and CL cases in ES to identify the principal vector and risk areas of the disease, aiming to understand the early origin and spread of this disease. Sand flies were collected in 466 rural localities between 1997 and 2013 during the three hours after evening twilight using a combination of active and passive capture. Insects were identified to the species level, and the localities were georeferenced. All autochthonous cases of CL treated at the University Hospital Cassiano Antonio Moraes (HUCAM) between 1978 and 2013 were evaluated. Twenty-one climate databases were selected from WorldClim. Maxent was used to construct potential distribution models for *Lu. intermedia*, *Lu. whitmani*, *Lu. migonei*, *Lu. lenti*, *Lu. choti* and CL cases. ENMTools was used to overlap the species and the CL case models. The Kruskal-Wallis and qui-quadrado tests were performed, adopting a 5% significance level. The 249,783 specimens captured represented 43 species. Of the 1,423 autochthonous cases recorded, 10.8% presented mucosal lesions. The area under the curve (AUC) was considered acceptable for *Lu. intermedia*, *Lu. whitmani*, *Lu. migonei*, *Lu. lenti*, *Lu. choti* and the CL cases. Topography was considered relevant to the construction of the models for all the species identified. *Lutzomyia intermedia* and *Lu. migonei* showed relevance with some variables, such as topography, BIO13, BIO12, and altitude. In order, variables such as topography, BIO13, and BIO15 were important to *L. lenti* and *L. whitmani*. For *L. choti*, topography, BIO4, BIO18, BIO17, and altitude presented as relevant variables. The overlay test identified *Lu. intermedia* as the main vector of CL in the study area. There is evidence of the existence of a primitive wild cycle of LTA in Atlantic forest areas in southeastern Brazil. It is possible that *L. braziliensis* has been transferred from the Amazon region to the Atlantic forest areas, for thousands of years through forest corridors linking the two biomes. There were differences between the contagion regions of patients, indicating that there was CL expansion to the east of ES, possibly caused by the intensification of migration to the urban center. Spatial modelling tools enable an analysis of the association among environmental variables, vector distributions, and CL cases in ES. Further, they allow better understanding of the factors related to the CL geographical spread in colonized areas of the Southeast of Brazil.

**First recording of *Sciopemyia vattierae* (Diptera: Psychodidae: Phlebotominae) in the State of Acre, Southeast of the Brazilian Amazon**

Márcia Moreira de Ávila^1^, Andreia Fernandes Brilhante^2^, Eunice Aparecida Bianchi Galati^2^, Reginaldo Peçanha Brazil^3^

^1^Instituto Federal do Acre (IFAC), Brasil

^2^Faculdade de Saúde Pública da Universidade de São Paulo, Brasil

^3^Fundação Oswaldo Cruz, Brasil

marcia.avila@ifac.edu.br

Sand flies (Diptera, Psychodidae, Phlebotominae) of various species are implicated in the transmission of *Leishmania* protozoans to humans and other vertebrates. Currently, 85 sand fly species representing several genera are known to occur in the Brazilian state of Acre. In this study, the occurrence of *Sciopemyia vattierae* (Le Pont & Desjeux, 1992), heretofore restricted to Peru (PE), Colombia and Bolivia, is reported in Brazil. During collections undertaken with CDC-light traps from December 2014 to January 2016 in forest and peridomilary areas of a rural settlement and in forested areas of an urban park located in the city of Rio Branco, Acre, three males and five females of *Sc. vattierae* were collected. The presence of papillae on flagellomere III distinguishes *Sc. vattierae* and *Sciopemyia sordellii* from the other species of the genus, in which the papillae are absent. The distinction of males of these two species was based on morphometric characters (lengths of FI and aedeagal ducts) and morphological characteristics were used to distinguish the females (In *Sc. vattierae*, the terminal knob clearly separated from the spermatheca and whereas it is sessil in *Sc. sordellii*). Thus, with this finding the geographical distribution of *Sc. vattiearae* in South America and the number of species of sand flies in Acre state are enlarged.

**Sand fly fauna (Diptera: Psychodidae) in an endemic area of American cutaneous leishmaniasis of Brazilian Amazon**

Márcia Moreira de Ávila^1^, Andreia Fernandes Brilhante^2^, Eunice Aparecida Bianchi Galati^2^, Reginaldo Peçanha Brazil^3^

^1^Instituto Federal do Acre (IFAC), Brasil

^2^Faculdade de Saúde Pública da Universidade de São Paulo, Brasil

^3^Fundação Oswaldo Cruz, Brasil

marcia.avila@ifac.edu.br

Cutaneous leishmaniasis is a zoonosis with wide geographic distribution and with different *Leishmania* species as etiological agents. As in most states of the Brazilian Amazon regions, the emergence of the disease in Acre seems to be related to anthropic actions and human occupation. This study aimed to identify the sand fly fauna in Rio Branco, capital of the state of Acre, Brazil. The sand flies were captured in a rural area and in an urban park of the municipality using light traps (CDC-type) once a month, from December 2014 to January 2016. We collected a total 2,210 sand flies belonging to 13 genera and 37 species. The most frequent was *Trichophoromyia auraensis* (43.2%) followed in descending or by *Trichophoromyia* sp. (27.4%), *Pressatia calcarata* (7.3%), *Pressatia* sp. (4.1%), *Evandromyia saulensis* (6.1%), *Ev. walkeri* (3.9%), *Psychodopygus carrerai carrerai* (3.6%), *Bichromomyia flaviscutellata* (2.7%), *Nyssomyia whitmani* (1.6%) and *Migonemyia migonei* (0.1%). Of the species found in Rio Branco, three are known vectors of *Leishmania* in the Amazon region: *Ny. whitmani*, *Bi. flaviscutellata* and *Mg. migonei*. The results show that the sand fly fauna is diverse and includes incriminated and proven vectors of *Leishmania.* This information will be of use to the epidemiological surveillance team of the Rio Branco municipality in establishing control actions in the urban and rural areas where there are reported leishmaniasis cases.

**Environmental factors and the occurrence of *Nyssomyia whitmani* and *Lutzomyia cruzi* in the Brazilian Central West**

Wagner de Souza Fernandes^1^, Anny Keli Aparecida Alves Cândido^2^, Leandro Machado Borges^1^, Aline Etelvina Casaril^1,3^, Jucelei de Oliveira Moura Infran^3^, Elisa Teruya Oshiro^3^, Suellem Petilim Gomes^1^, Antônio Conceição Paranhos Filho^2^, Eliane de Mattos Piranda^3^, Alessandra Gutierrez de Oliveira^1,3^

^1^Federal University of Mato Grosso do Sul, Postgraduate Program in Infectious and Parasitic Diseases, Campo Grande, MS, Brazil

^2^Federal University of Mato Grosso do Sul, Geoprocessing Laboratory for Environmental Applications, Campo Grande, MS, Brazil

^3^Federal University of Mato Grosso do Sul, Laboratory of Parasitology/CCBS, MS, Brazil

alessandra.oliveira@ufms.br

Biological and ecological relationships between vectors and their pathogens are important for understanding the epidemiology of vector transmission disease. Some sand flies species are vectors of *Leishmania*, among of them, *Lutzomyia cruzi* and *Nyssomyia whitmani* incriminated as vectors of *Leishmania* (*Leishmania*) *infantum* and *Leishmania* (*Viannia*) *braziliensis*, respectively*.* The municipality of Camapuã (Mato Grosso do Sul state, Brazil) is considered an endemic area for visceral and tegumentary leishmaniasis. We related, descriptively, the phytophysiognomy of ecotopes and the presence of *Lu. cruzi* and *Ny. whitmani* in Camapuã. A total of 24 captures were carried out, bimonthly, using Falcão automatic light traps intra and peridomicile, from May 2014 to April 2015. We calculated the Normalized Difference Vegetation Index (NDVI) from images obtained by LANDSAT 8. These images were classified according to the NDVI as follows, water (−1.00/+0.00), exposed soil (+0.00/+0.30), pothole vegetation (+0.30/+0.50), savannah (+0.50/+0.60) and forest (+0.60/+1.00). In total, 2,005 sand flies from nine species were collected. *Nyssomyia whitmani* (55.3%) and *Lu. cruzi* (41.3%) were the most representative among all collected species. *Nyssomyia whitmani* predominated in periurban neighborhoods. In these areas, the NDVI average values were higher than 0.50, demonstrating the presence of dense vegetation surrounding dwellings, consequently, the capture sites were shaded and there was considerable amount of organic matter in the soil. *Lutzomyia cruzi* was more frequent in urban neighborhoods, with NDVI average values below 0.50. We observed the preference of *Ny. whitmani* for preserved areas while *Lu. cruzi* was more commonly found in urban areas.

Financial support: FUNDECT and CAPES.

**Normalized Difference Vegetation Index (NDVI) in the characterization of sand flies environments**

Helen Rezende de Figueiredo^1^, Anny Keli Aparecida Alves Cândido^2^, César Claudio Cáceres Encina^2^, Everton Falcão de Oliveira^2^, Jaíza Santos Motta^2^, Jucelei de Oliveira Infran^3^, Aline Etelvina Casaril^1,3^, Elisa Teruya Oshiro^3^, Antonio Conceição Paranhos Filho^2^, Alessandra Gutierrez de Oliveira^1,3^

^1^Federal University of Mato Grosso do Sul, Postgraduate Program in Infectious and Parasitic Diseases, Campo Grande, MS, Brazil

^2^Federal University of Mato Grosso do Sul, Geoprocessing Laboratory for Environmental Applications, Campo Grande, MS, Brazil

^3^Federal University of Mato Grosso do Sul, Laboratory of Parasitology/CCBS, MS, Brazil

alessandra.oliveira@ufms.br

The biology and ecology of sand flies can suffer environmental influences as well as its interaction with vertebrate hosts in a particular breeding site. In Brazil, the State of Mato Grosso do Sul has lost native vegetation due to intense urbanization and this change has modified the habitat of sand flies and consequently the epidemiology of leishmaniasis. We characterized the environments of the urbanized area of Aquidauana (MS) associating land cover changes to the frequency of sand flies. Every fifteen days, 48 captures were carried out in intra and peridomicile areas with automatic light traps, from April 2012 to March 2014. We used the Normalized Difference Vegetation Index (NDVI) obtained from Landsat 5 and 8 to define types of vegetation in the collection sites and their surroundings. It was collected a total of 3,179 specimens in 10 species. *Lutzomyia longipalpis* presented 93.02% of the total, followed by *Evandromyia walkeri* (4.25%) and other species 2.74%. *Lutzomyia longipalpis* was observed in all months and collection sites including areas with higher degrees of anthropization. The NDVI was efficient to characterize the environments. It was possible to observe the relationship between the amount of phytomass by NDVI and frequency, abundance, distribution and diversity of sand flies.

Financial support: FUNDECT and CAPES.

**Preliminary information on insular sand flies, in Paraná River, Brazil**

Barbara Andreo dos Santos^1^, Kárin Rosi Reinhold-Castro^1^, Herintha Coeto Neitzke-Abreu^2^, Edilson Colhera Cristóvão^3^, Thaís Gomes Versignassi Silveira^1^, Ueslei Teodoro^1^

^1^Programa de Pós-Graduação em Ciências da Saúde, Universidade Estadual de Maringá, Brasil

^2^Faculdade de Ciências da Saúde, Universidade Federal da Grande Dourados, Brazil

^3^Secretaria Municipal de Saúde de Porto Rico, Núcleo de Entomologia, Brasil

karindecastro@yahoo.com.br

Leishmaniasis is a neglected disease with worldwide distribution, caused by the protozoan parasite *Leishmania*; which is transmitted to humans by the bites of infected female phlebotomine sand flies. Nowadays, 988 species of sand flies have been described. American species comprise 529 of these (512 contemporary and 17 fossils). In Brazil, 19 species are suspected or implicated in the transmission of *Leishmania* to humans. Among these, the most common are *Nyssomyia neivai* (Pinto), *Migonemyia migonei* (França), *Ny. whitmani* (Antunes & Coutinho), *Pintomyia pessoai* (Coutinho & Barreto) and *Pi. fischeri* (Pinto). The aim of this study was to gain preliminary knowledge of these insects on some islands in the Paraná River that belong to municipalities of Porto Rico, Querência do Norte, and São Pedro do Paraná, Paraná State, Brazil. Sand fly collections were made with Falcão light traps in the edges of and within forests, domestic animal shelters, and domiciles. The collections on Bandeira, Carioca and Fina Islands were conducted in June, August and October 2012; on Mutum Island, from October 2013 to October 2014; on Japonesa and São José Catarino Islands, from November 2013 and January, June, and September 2014; on Floresta Island, in December 2013, January, June, and September 2014; on Chapéu Velho Island, in December 2013, January, April, and June 2014. In Cruzeiro Island, in January, June, September, and October 2014; on Santa Rosa Island, in December 2013, January, April, and August 2014. On Mutum Island, which has a large number of permanent residents, the collections were conducted during one night each month, from 18:00 to 6:00 h, during 13 months. On the other islands, with smaller numbers of residents, one night’s collection during each season (fall, winter, spring, and summer), from 19:00 to 7:00 h, during one year. The sand flies collected were sacrificed with chloroform and placed in tubes containing 80% alcohol for their preservation and subsequent identification. A total of 55,761 sand flies were collected. In 2012, collections included 19,818 specimens of *Ny. neivai* were collected, (16,746 females and 3,072 males), and two females of *Ny. whitmani* and a female *Psathyromyia shannoni* (Dyar). In 2013 and 2014, 35,943 specimens of *Ny. neivai* were collected (31,414 females and 4,529 males) and one *Brumptomyia brumpti* (Larrousse). *Ny. neivai* was the predominant species, accounting for 99.9% of the collected specimens. A previous study also reported the capture of *Br. cunhai* (Mangabeira). In the inhabited islands, a large number of insects were collected in domestic animal shelters, which are close to domiciles. The presence of these animals insures blood sources, which sustain sand fly growth and density in these ecotypes, increasing the risk for locals and tourists who frequent the islands. The small number of species collected in relation to the 49 described in the Paraná state may be due to the isolation of the islands and the inability of other species to adapt to the insular environment. *Ny. neivai* is a proven vector of cutaneous leishmaniasis (CL) and also the dominant species in the studied islands and the most common in all ecotypes, especially in domestic animal shelters and human domiciles. The capacity of a vector to transmit pathogens is related to its density. Thus, our results indicate the necessity of strategies to reduce human-vector contact.

Financial support: Coordenação de Aperfeiçoamento de Pessoal de Nível Superior (CAPES).

**Entomological surveillance in *Leishmania* transmission areas on the border between Brazil and Bolivia**

Elizabeth F. Rangel^1^, Adriana Zwetsch^1^, Antônio Luís F. Santana^1^, Rodrigo E. Godoy^1,2^, Júlia S. Silva^1^, José O. Silva^3^, Paulo S. Almeida^3^, Zaida E. Yadon^4^

^1^Fundação Oswaldo Cruz (FIOCRUZ), Laboratório Interdisciplinar de Vigilância Entomológica em Diptera e Hemiptera/Lab. de Referência Nacional em Vigilância Entomológica, Taxonomia e Ecologia de Vetores de Leishmanioses, Brasil

^2^Departamento de Epidemiologia, Faculdade de Saúde Pública, Univer. de São Paulo, SP, Brasil

^3^Secretaria de Estado de Saúde de Mato Grosso do Sul, Laboratório Regional de Saúde de Dourados, Brasil

^4^Communicable Diseases and Health Analysis Department, Pan American Health Organization, Washington, DC, USA

adrianaz@ioc.fiocruz.br

The leishmaniases are emerging and re-emerging diseases in American countries. In Brazil, American Cutaneous Leishmaniasis (ACL) is registered in all states and American Visceral Leishmaniasis (AVL) in most of them, both with significant impact on human health. Bolivia records leishmaniasis in six of its nine departments. In the valley of “Yungas”, cutaneous and mucocutaneous leishmaniasis are public health issues, with 20% prevalence. The AVL, rare in the Andean countries, has been recorded in Bolivia with ten sporadic cases to date. Few studies have been conducted in relation to the *Leishmania* vectors in Bolivia, including border areas with Brazil. The study aimed to identify the species of sand fly vectors on the Brazil-Bolivia border; to know the spatial distribution associated with the environment and risk factors for transmission. Monitoring stations were established (MS) on both sides. In Brazil, in Corumbá (AVL – intense transmission area), for each MS, traps were set in peridomicilary sites (animal shelters) and in sites next to the forest. Captures (October and November/2015) were done with CDC light traps installed in areas surrounding houses and close to forest, and operated for 12 h from the start of the evening, for four consecutive nights. Six MS were set in a military field, in the main access to Bolivia (City of Arroyo Concepción), next to the main bridge that has a large flow of people, animals and vehicles. Four MS were located on small farms, one just a kilometer from the border. In Bolivia, in the city Arroyo Concepción, Department of Santa Cruz, the only MS studied was the one close to MS6 (in Brazil), using the same methodology, with two nights of captures. At this site, CDC light traps were installed in October close to a small river and in a henhouse; and in November only near the river. The methods for taxonomic identification follow the proposal by Galati (2003). Preliminary results from 120 h of captures in Brazil and 36 h in Bolivia include twelve identified sand fly species: *Brumptomyia brumpti*, *Evandromyia aldafalca*o*ae*, *Ev. corumbaensis*, *Ev. sallesi*, *Lutzomyia cruzi*, *Micropygomyia peresi*, *Psathyromyia ara*ga*oi*, *Pa. bigeniculata*, *Pa. dendrophyla*, *Pa. punctigeniculata* and *Sciopemyia sordellii*, totaling 1,394 speci*mens* from Brazil; and *Br. brumpti*, *Ev. corumbaensis*, *Lu. cruzi*, *Mi. peresi* and *Mi. quinquefer*, totaling 22 specimens from Bolivia. Noteworthy is the predominance of *Lu. cruzi* in all MS, including the home environment. *Lutzomyia cruzi* is the vector of AVL in endemic areas in the Central Region of Brazil. Possibly there is no geographical barrier to sand fly dispersion across the border between the two countries, may explain the record of *Lu. cruzi* on the Bolivian side. It is of particular interest that to date, the *Lu. longipalpis* appears to be absent at the monitoring stations in both Brazil and Bolivia, suggesting that *Lu. cruzi* is participating in the AVL transmission in Bolivia, as it is in Brazil.

Financial support: Small Grant Programme Tropical Diseases Research/Pan American Health Organization 2015. No: IOC-013-PPE-15; Instituto Oswaldo Cruz/ FIOCRUZ, RJ, Brazil; FAPESP, SP, Brazil.

**Analysis of feeding preference of sand flies captured in peridomestic habitats in Panama**

Ana Rosa Caballero^1^, Anayansi Valderrama^2^

^1^Panama Latin University, Panama

^2^Institute Conmemorative Gorgas, Panama

avalderrama@gorgas.gob.pa

Leishmaniasis comprises a group of parasitic diseases caused by about 13 species and subspecies of protozoans of the genus *Leishmania*. Parasites of this genus are transmitted to humans by the bites of phlebotomine sand flies. In Panama, vector species that exhibit the highest degree of anthropophily and relevance in the transmission of *Leishmania* in Panama include *Lutzomyia panamensis*, *Lu. trapidoi* and *Lu. ylephiletor*. Knowledge of preferences in their hematophagy can provide indirect information on potential reservoir hosts and facilitate better understanding of leishmaniasis ecology under natural conditions. For this, molecular techniques involving the amplification of a region of cytochrome *b* to were use to identify mammalian and avian sources of blood meals in midguts of relevant *Lutzomyia* species to determine blood-feeding preferences. An analysis was applied by simple correspondence resulting in a χ^2^: 57.24, *df*: 18, *p* < 0.05. It was observed that *Lu. trapidoi* and *Lu. panamensis* prefer to feed on humans and swine, while *Lu. ylephiletor* shows a preference for dogs or other mammals in general. This preliminary information helps clarify ecological aspects *Leishmania* transmission in perdomicilary hábitats, providing valuable knowledge for prevention of leishmaniasis.

**Why sand fly samplings of a single night are insufficient? An analysis in an urban area of northeast Argentina based on light traps**

Maria Soledad Santini^1^, Maria Soledad Fernández^2^, Maria Gabriela Quintana^3,4^, Regino Cavia^2^, Oscar Daniel Salomón^4^

^1^Centro Nacional de Diagnóstico e Investigación en Endemo-epidemias (CeNDIE), ANLIS, Ministerio de Salud de la Nación, Buenos Aires, and Consejo Nacional de Investigaciones Científicas y Técnicas (CONICET), Argentina

^2^Dto. de Ecología, Genética y Evolución, FCEN, UBA and Instituto de Ecología, Genética y Evolución de Buenos Aires (UBA-CONICET), Buenos Aires, Argentina

^3^Instituto Superior de Entomología (INSUE-UNT), Tucumán, and CONICET, Argentina

^4^Instituto Nacional de Medicina Tropical (INMeT), Ministerio de Salud de la Nación, Puerto Iguazú, Misiones, and CONICET, Argentina

mariasoledadsantini@gmail.com; msantini@anlis.gov.ar

In recent years, in Argentina, entomological surveys to study the occurrence and abundance of sand flies of epidemiological interest have become more frequent. Indicators of infestation levels, as the proportion of sampled sites where a particular sand fly species occurs, or its abundance, were usually estimated, as well as other community characteristics, such as the number of species present (richness). Some of these surveys cover large areas, so the number of nights that lights traps remains active, as well as the total number of light traps, has a cost in relation to human and material resources. The aim of this work was to study how the estimation of the proportion of sampled sites where a particular species occurred, the species abundances – trap success – and the richness changed when the light traps remained active for more than one night. We analyzed data from four entomological surveys conducted in the city of Puerto Iguazú, northeastern Argentina (summer 2011, fall and winter 2012 and spring 2014) at 49–55 sampling sites selected with worst-scenario criteria. In each site, a light trap for capturing Phlebotominae was active during two consecutive nights in summer 2011, fall and winter 2012 and three consecutive nights in spring 2014. *Lutzomyia longipalpis* and *Nyssomyia whitmani* (*Leishmania infantum* and *L. braziliensis* main vectors in the study area, respectively), were the species more frequently captured. For these vectors, the estimation in the percentage of sites with presence increased between 23% and 75% (depending on the species and the season) with two nights compared to the estimation of a single night. The pattern for the average number of captures showed that the estimation did not change substantially but the coefficients of variation slightly decreased. The richness increased with the number of trap nights in all samples. Our results show the importance of avoiding single-night sampling due to the increase in the estimated percentage of sites with occurrence of vectors with a two-night protocol and in the richness also with a three night protocol. As more nights are sampled, the probability of detection of sand flies of different species increases (even medically important species), and the variability between nights is compensated. However, due to cost restrictions, complementary studies are in progress to determine the optimal minimum number of nights to estimate these and other variables in different environments and seasons. Also, other characteristics of the survey, such as the type of trap (or traps) used and the main objective of the survey should be considered. Currently in Argentina, where operationally possible, the general recommendation is a three-night capture protocol.

**Identification of natural breeding sites of sand flies in an endemic zone of cutaneous leishmaniasis in Argentina**

Mariana Manteca Acosta^1^, Maria Soledad Santini^2,3^, Maria Eugenia Utgés^2^, Oscar Daniel Salomón^1,3^

^1^Instituto Nacional de Medicina Tropical (INMeT), Ministerio de Salud de la Nación, Puerto Iguazú, Misiones, Argentina

^2^Centro Nacional de Diagnóstico e Investigación en Endemo-epidemias (CeNDIE), ANLIS, Ministerio de Salud de la Nación, Buenos Aires, Argentina

^3^Consejo Nacional de Investigaciones Científicas y Técnicas (CONICET), Argentina

mariasoledadsantini@gmail.com

The location of the microhabitats where immature stages of phlebotomine sand flies develop is one of the least-known aspects of sand fly biology because these stages are difficult to detect in their terrestrial breeding sites. This project was developed in a rural area (Misiones, northeastern Argentina) where epidemic outbreaks of cutaneous leishmaniasis due to *Leishmania braziliensis* took place during 2004–2005, with *Nyssomyia whitmani* as the main vector. The houses in this zone are built typically on stilts, with floors elevated above the ground, providing a microhabitat with suitable and stable temperatures, high humidity and a resting place for domestic and even synanthropic animals. The objective of this work was to detect the presence of natural sand fly breeding sites both in chicken sheds and under the stilt houses, presumably the more suitable places for phlebotomine larvae. From October 2015 to April 2016, a total of 439 emergence traps were set to sample these two microhabitats (designated as “chicken shed” and “below house”) at each of eight farms. The area of each microhabitat was divided into squares of 40 × 40 cm, and an emergence trap was set inside each square. The traps were checked fortnightly and relocated every 40 days within the same square, considering the average lab-based time spent for development of the immature stages. Emergent adults were collected in 40 (9%) of the total traps placed, 21 from “chicken shed” sites and 19 from “below house” sites. We collected 79 sand flies: 77 *Ny. whitmani*, one *Pyntomyia monticola* and one *Expapillata firmantoi*. The proportion of *Ny. whitmani* positive emergence traps in chicken sheds was not significantly higher than the proportion from “below house” sites (χ^2^ = 0.82, *df* = 1, *p* > 0.05). For the chicken shed microhabitat, the total density was 2.7 individuals per square, and for the below house microhabitat only 1.1 individuals per square. However, we did not find significant differences between the average number of individuals per square in “chicken shed” sites (0.03, DS 0.04) and “below house” sites (0.02, DS 0.02) (*F* = 1.26, *df* = 1, *p* = 0.28). *Nyssomyia whitmani* was collected during each month of the study, with a peak in November–December 2015, consistent with previous adults captures. Along the northeastern border of Argentina, the chicken shed- and below house-microhabitats are suspected as potential natural breeding sites for phlebotomine sand flies, mainly *Ny. whitmani*, vector of *Leishmania braziliensis*, the causative agent of cutaneous leishmaniasis in this area.

**Update on Phlebotominae fauna from the Chaco region, Argentina**

Enrique Alejandro Szelag^1,3,4^, Jose Dilermando Andrade Filho, Juan Ramón Rosa^3^, María Gabriela Quintana^1,4,5^, Oscar Daniel Salomón^1,5^

^1^Instituto Nacional de Medicina Tropical, Misiones, Argentina

^2^Centro de Referência Nacional e Internacional para Flebotomíneos, Grupo de Estudos em Leishmaniose, Centro de Pesquisas René Rachou, Belo Horizonte, Brazil

^3^Instituto de Medina Regional, Chaco, Argentina

^4^Consejo Nacional de Investigaciones Científicas y Tecnológicas (CONICET). C.A.B.A, Argentina

^5^Instituto Superior de Entomología “Dr. Abraham Willink”, San Miguel de Tucumán. Argentina

odanielsalomon@gmail.com

Previous studies showed that the Phlebotominae fauna in Argentina consists of 31 species distributed in 14 of the 24 provinces. This work aims to provide an update on the Phlebotoinae fauna and species distribution in the Chacoan region. Biogeographically, the Chaco region is divided by its annual rainfall gradient into two clearly differentiated subregions: the Eastern region (Wet Chaco) and the Western region (Dry Chaco). The first presents a damp Atlantic rainfall pattern where biomes form parks and savanna with abundant rainfall in summer and fall. On the other hand, the Western region has a dry continental and semi-arid rainfall pattern. Captures were conducted monthly in the two biogeographic regions using CDC miniature light placed 1.5 m above the ground, and operating for 12 h (from 7 pm to 7 am). In Western Chaco region, captures were made during the period 2006–2008 for two consecutive nights each month in Nueva Población (S24°58′18″–W61°21′25″). In the Eastern Chaco region, captures were made monthly for overnight from 2009 to 2013 in the towns of Margarita Belen (S27°26′34″–W58°54′13″), Colonia Benitez (S27°19′16″–W58°59′53″), Resistencia (S27°16′48″–W59°1′11″), Tres Isletas (S26°20′24″–W60°25′52″). In the Transitional Area (Wet Chaco/Dry Chaco) of Pampa del Indio (S25°52′29.9″; W59°49′25.5″), monthly collections for two consecutive nights were made from 2013 to 2015. At each collection site, three light traps were set, one intradomicilary, one extradomicilary and one in a nearby peridomestic habitat. Species previously recorded for Argentina: *Brumptomyia avellari*, *Br. brumpti*, *Br. guimaraesi*, *Br. pintoi*, *Evandromyia evandroi*, *Ev. cortelezzii*, *Ev. sallesi*, *Expapillata firmatoi*, *Micropygomyia oswaldoi*, *Mi. peresi*, *Mi. quinquefer*, *Migonemyia migonei*, *Martinsmyia alphabetica*, *Nyssomyia neivai*, *Ny. whitmani*, *Lutzomyia longipalpis*, *Oligodontomyia* spp., *Pintomyia bianchigalatiae*, *Pi. fischeri*, *Pi. pessoai*, *Pi. misionensis*, *Pi. monticola*, *Pi. torresi*, *Pi. damascenoi*, *Psathyromyia pascalei*, *Pa. lanei*, *Pa. punctigeniculata*, *Pa. baratai*, *Pa. bigeniculata*, *Sciopemyia sordellii*, *Trichophoromyia auraensis.* New records for Argentina: – *Ev. aldafalcaoae* Margarita Belén: 1 male 06-II-2012; Resistencia: 1 male 04-XII-2013. Pampa del Indio: 1 male, 2 females 10-I-2013; 1 male, 1 female 11-III-2014; 4 males, 3 females 09-IV-2015. Caught in peridomestic site (associated with kennels and pigsty), intradomestic site (veranda) and edge of forest. – *Ev. corumbaensis*: Colonia Benítez: 1 male 19-III-2012; Resistencia: 2 males 09-IV-12 and 17-I-2013. Western biogeographic region – Nueva Población: 7 males 21-XI-2006, 09-I-2007, 21-III-2007, 01-X-2007 and 13-XII-2007; 8 females 25-IV-2007, 01-X-2007, 13-XII-2007 and 15-XII-2008, captured in peridomestic and extradomestic sites. *– Ev. termitophila*: Nueva Población: 1 male 10-X-2008; Tres Isletas: 1 female 04-IV-2011, captured in intradomestic site and peridomestic sites associated with a pigsty and a henhouse. New record for Chaco: – *Pa. bigeniculata*: Margarita Belen, 6 males 15-XII-2011, 2 males 27-III-2013 captured in peridomestic (pigsty and henhouse) and extradomestic sites. These new records extend the southern limit of known distribution for these species, including the southernmost record on the continent, and also extend the total number of known species in the country to 34.

Financial support: Alberto J. Roemmers Argentina Foundation and Bunge & Born Foundation.

**Phlebotominae: spatial-temporal distribution in Corrientes city, Argentina**

Pablo E. Berrozpe^1,2,5^, Maria Soledad Santini^2,3,5^, A.V. Araujo^4,5^, D. Lamattina^1^, Oscar Daniel Salomon^1,2,5^

^1^Instituto Nacional de Medicina Tropical (INMeT), Ministerio de Salud de la Nación, Puerto Iguazú, Misiones, Argentina

^2^Consejo Nacional de Investigaciones Científicas y Técnicas (CONICET)

^3^Centro Nacional de Diagnóstico e Investigación en Endemo-epidemias (CeNDIE), ANLIS, Ministerio de Salud de la Nación, Buenos Aires, Argentina

^4^Universidad Nacional del Nordeste, Corrientes

^5^Red de Investigación de las Leishmaniasis en Argentina

mariasoledadsantini@gmail.com

Reports of cases of visceral leishmaniasis (VL) in canine reservoirs in Corrientes city prompted a search for sand fly vectors in 2008. The presence of three species was confirmed: *Lutzomyia longipalpis*, vector of the causative agent of VL, *Nyssomia neivai* and the species complex *Evandromyia cortelezzi-sallesi*, vectors of the causative agents of Tegumentary Leishmaniasis (TL). Based on this information, this study was designed to study the spatio-temporal distribution of the Phlebotominae of Corrientes, in order to establish seasonal (temporal) and environmental (spatial) profiles sand fly relative abundance as indicators of risk of exposure to sand fly bites. Relying on this information, it is hoped that distribution models can be developed and validated in other endemic areas. Sociodemographic and environmental data (taken from satellite images) were used to define three strata: rural immersed in a peri-urban matrix, peri-urban and urban. The sampling period was from September 2014 to August 2015, two samplings per season, and consisted of placing REDILA-BL minilight traps in six peridomiciliary sites of each identified stratum for three consecutive, rainless nights. The sites were selected under “worst case scenario” criteria. Captured sand fly specimens were kept dry until diaphanized with lactophenol and identified using an optical microscope, following Galatti (2005). Spring: 662 Phlebotominae, 66% from rural stratum, 28% from peri-urban and 6% from urban stratum. Species: Rural stratum – *Lu. longipalpis* (91%), *Nyssomyia. neivai* (7.5%), *Migonemyia migonei* (1.4%) and a single female *Ev. cortelezzi-sallesi*. Peri-urban stratum – *Lu. longipalpis* (83.7%), *Ny. neivai* (15.7%) and a single female *Mg. migonei*. Urban stratum – *Lu. longipalpis* (100%). Summer: 2208 Phlebotominae, 77% from rural, 21% from peri-urban and 2% from urban stratum. Species: Rural stratum – *Lu. longipalpis* (30%), *Ny. neivai* (57%), *Mg. migonei* (10.5%) and *Ev. cortelezzi-sallesi* (2%) and 3 female *Micropigomyia quinquefer*. Peri-urban stratum – *Lu. longipalpis* (50%), *Ny. neivai* (40%), *Mg. migonei* (9%) and a single female *Ev. cortelezzi-sallessi*. Urban stratum – *Lu. longipalpis* (100%). Autumn: 723 Phlebotominae, 91% from rural stratum, 6% from peri-urban and 3% from urban stratum. Species: Rural stratum – *Lu. longipalpis* (11%), *Ny. neivai* (80%), *Mg. migonei* (8.5%) and three female *Ev. cortelezzi-sallesi*. Peri-urban stratum – *Lu. longipalpis* (68%), *Ny. neivai* (25%), *Mg. migonei* (7%). Urban stratum – *Lu. longipalpis* (100%). Winter: 80 Phlebotominae, 56% from rural stratum, 15% from peirurban and 19% from urban stratum. Species: Rural stratum – *Lu. longipalpis* (27%) and *Ny. neivai* (73%). Peri-urban stratum – *Lu. longipalpis* (80%), *Ny. neivai* (20%). Urban stratum – *Lu. longipalpis* (100%). The spatial distribution reveals that *Lu. longipalpis* is the only species present in all three environments. The remaining species were restricted to peri-urban and mostly to the rural stratum. The temporal distribution shows a bimodal abundance curve with a peak in late spring and the other in late summer. This study identifies risk strata and moments of higher probability of contact between humans and vectors for the study area. Differences in abundances between environmental strata could indicate that populations of phlebotomine species behave as metapopulations with recolonizations, in times of greater abundance, from the rural stratum to the other strata, and that *Lu. longipalpis* is the species with best colonization success.

**Distribution, abundance, and genetic variability of *Lutzomyia longipalpis* (Diptera: Psychodidae) in Tartagal city, Salta, Argentina**

María Gabriela Quintana^1,2,3,4^, María Soledad Santini^2,4,5^, Andrea Gómez Bravo^2,6^, Ana Denise Fuenzalida^1,2,3^, Mariana Manteca Acosta^1,2^, Angélica Pech-May^1,2,4,7^, Oscar Daniel Salomón^1,2,4^

^1^Instituto Nacional de Medicina Tropical-MSN, Argentina

^2^REDILA, Argentina

^3^Instituto Superior de Entomología-UNT, Argentina

^4^CONICET, Argentina

^5^Centro Nacional de Diagnóstico e Investigaciones en Endemoepidemias-ANLIS-MSN, Argentina

^6^Fundación Mundo Sano, Argentina

^7^Instituto Nacional de Salud Pública/Centro Regional de Investigación en Salud Pública, Tapachula, Chiapas, México

gabrieladealquintana@gmail.com

*Lutzomyia longipalpis* was first reported in northeastern Argentina in 2004, and from there began its spread southwards. Three human cases of visceral leishmaniasis (VL) were reported without the confirmed presence of *Lu. longipalpis* between 2008 and 2011 in the province of Salta, located in the northwest of the country. In the northeast, the first human cases were reported in 2006 and to date there have been 153 cases reported. In 2013, phlebotomine sand fly collections were made in the city of Tartagal, Salta, when the presence of *Lutzomia longipalpis* was first reported in northwestern Argentina. Subsequently, intensive sampling was done in this city in order to study the distribution pattern and stratify the potential transmission risk of *Lesihmania infantum*. The city was divided into 400 × 400-meter quadrants and 66 sites/households were selected according to “worst case scenario” criteria. Collections were made for three consecutive nights with CDC-type miniature light traps. In order to correlate and characterize those sites with high sand fly abundance, the following variables were explored: micro variables/macro-habitats taken in situ (10 m^2^), and environmental variables taken from high-resolution image. At the same time, samples of *Lu. longipalpis* males from two different sites approximately 1500 m apart were analyzed to study the genetic variability of the populations (ND4 gene). A total of 709 sand flies were collected comprising the following species: *Evandromyia cortelezzii-sallesi* complex (486), *Migonemyia migonei* (139), *Lutzomyia longipalpis* (82) and *Nyssomyia neivai* (2). Of the sampled sites, 54 yielded sand flies, and 16 sites were positive for *Lu. longipalpis* (24%). Of the *Lu. longipalpis*, the ratio of males to females was 1:1; 25% of the females were gravid, and 27% with blood in abdomen. Regarding the variables explored in relation to the abundance of *Lu. longipalpis*, correlation was found with the following: soil coverage and the number of dogs (micro-habitats); square meters of waterlogged areas (macro-habitats); and the environmental variables were: normalized difference vegetation index (NDVI) average of a 50 m^2^ buffer around the trap, and the normalized difference water index (NDWI) within 5 m^2^. Genetic variability between sand fly populations at the two analyzed sites was high, with 14 different haplotypes, with only one shared by both populations. These preliminary results reveal an incipient colonization and interesting population variability that demand intensified, especially in the border area with Bolivia to and elucidate the route of entry and/or dispersion of the main vector of *Lesihmania infantum* in northwestern Argentina.

Financial support: Bunge & Born Foundation, Ministry of Health of Argentina and Mundo Sano Foundation.

**Updated distribution records of phlebotomine sand flies (Diptera: Psychodidae) of Spain**

Javier Lucientes^1^, Rosa Estrada^1^, Vladimir Oropeza-Velasquez^1^, Sarah Delacour-Estrella^1^, Pedro María Alarcón-Elbal^1,2^, José Ignacio Ruiz-Arrondo^1^, Ricardo Molina^3^

^1^Departamento der Patología Animal, Facultad de Veterinaria, Instituto Agroalimentario de Aragón (IA2) (Universidad de Zaragoza-CITA), Spain

^2^Universidad Agroforestal Fernando Arturo de Meriño, Jarabacoa, Dominican Republic

^3^Laboratorio de Entomología Médica, Servicio de Parasitología, Centro Nacional de Microbiología, Instituto de Salud Carlos III. Madrid, Spain

jlucien@unizar.es

The Spanish Bluetongue Entomological Surveillance Programme was started in 2004 to monitor bluetongue vector populations at weekly intervals in the country. This viral disease is mainly transmitted among vertebrate hosts (principally domestic and wild ruminants) by several species of biting midges of the genus *Culicoides* (Diptera: Ceratopogonidae). The traps used were CDC miniature blacklight traps (Model 1212; John W. Hock Company, Gainesville, FL) were placed outside selected sheep or cattle farms and was run for one night each week from dusk until dawn. All collected arthropods were transported to the laboratory and preserved in 70% ethanol. Upon examination, species of medical and veterinary interest were separated from other insects. The captured phlebotomine sand flies were stored into separate tubes with 70% ethanol and taxonomic identification was made according to Lewis (1982) and Gállego et al (1992). Seven species were identified: *Sergentomyia minuta*, *Phlebotomus papatasi*, *Ph. perniciosus*, *Ph. ariasi*, *Ph. langeroni*, *Ph. sergenti* and *Ph. mascitti*. Of these, *Ph. perniciosus* is the most widespread vector of *Leishmania infantum* in the country. In the present work we present the updated distribution maps of sand flies species for the period 2004–2015 in peninsular Spain and Balearic Islands. No catches have been recorded in Canary Islands.

Financial support: Ministry of Agriculture, Food and Environment of Spain.

**Presence of *Phlebotomus* (*Transphlebotomus*) *mascittii* Grassi, 1908, in northern Spain: first record for the Cantabrian Region and second for the Iberian Peninsula**

Javier Lucientes^1^, Rosa Estrada^1^, Vladimir Oropeza-Velasquez^1^, Sarah Delacour-Estrella^1^, Pedro María Alarcón-Elbal^1,2^, José Ignacio Ruiz-Arrondo^1^, Ricardo Molina^3^

^1^Departamento de Patología Animal, Facultad de Veterinaria, Instituto Agroalimentario de Aragón (IA2) (Universidad de Zaragoza-CITA), Spain

^2^Universidad Agroforestal Fernando Arturo de Meriño, Jarabacoa, Dominican Republic

^3^Laboratorio de Entomología Médica, Servicio de Parasitología, Centro Nacional de Microbiología, Instituto de Salud Carlos III, Madrid, Spain

jlucien@unizar.es

Framed within the Spanish Bluetongue Entomological Surveillance Programme, CDC miniature black-light (UV) traps (Model 1212) were used in selected sheep and cattle farms nationwide. All collected arthropods were transported to the laboratory and preserved in 70% ethanol. Upon taxonomic identification, species of medical and veterinary interest were separated from other insects. The captured phlebotomine sand flies were identified according to Lewis (1982) and Gállego et al. (1992). During an entomological survey carried out in 2009, one male and five females of *Phlebotomus* (*Transphlebotomus*) *mascittii* were caught in the province of Cantabria, north-central Atlantic coast of Spain, specifically in the municipalities of Coo, Cobreces and San Juan de Soba. These are the first documented records of the occurrence of this species in the Cantabrian Region. *Phebotomus mascittii* was originally described from Italy and subsequently found in several Mediterranean regions from Spain in the west to Turkey in the east, and also in central European countries such as Switzerland, France, Belgium, Germany, and Austria. In the case of Spain, seven specimens of this species were collected in 1983 in the provinces of Barcelona and Gerona in the Autonomous Community of Catalonia, northeastern part of the Iberian Peninsula. Consequently, the presence of this phlebotomine sand fly is confirmed in Spain after been found for the second time in the Iberian Peninsula. Although the species has never been proven to be a vector of *Leishmania*, the scarcity of catches in the country makes this a relevant finding.

Financial support: Ministry of Agriculture, Food and Environment of Spain.

**Blood feeding behavior of *Phlebotomus perniciosus* collected in the human leishmaniasis focus of southwest Madrid, Spain, during the period 2012–2015**

Estela González^1^, Ricardo Molina^1^, Ana Tello^2^, Andrés Iriso^3^, Ángeles Vázquez^2^, Maribel Jiménez^1^

^1^Medical Entomology Unit, Parasitology Service, National Centre of Microbiology, Institute of Health Carlos III, Majadahonda, Madrid, Spain

^2^Zoology and Physical Anthropology Department, Faculty of Biological Science, Complutense University of Madrid, Madrid, Spain.

^3^Zoonosis and Biological Risk Section, General Directorate of Public Health, Madrid Regional Health Authority, Community of Madrid, Madrid, Spain

mjimenez@isciii.es

Seasonal entomological surveys performed monthly for three consecutive years from May to October at four stations neighboring an urban focus of human leishmaniasis in Madrid (Spain) have shown that *Phlebotomus perniciosus*, collected with both sticky and CDC light traps, is the only vector of *Leishmania infantum* in the area, with mean densities reaching 193.60 specimens/m^2^, as determined by sticky trap collections. In the same study the analysis of blood preferences of *Ph. perniciosus* revealed that they feed mainly on rabbits (*Oryctolagus cuniculus*) followed by hares (*Lepus granatensis*). Direct xenodiagnoses of leishmaniasis carried out on hares and wild rabbits from the focus proved that these lagomorphs are infective to colonized *Ph. perniciosus*, although in different proportions. The host preferences of sand flies collected in an entomological survey performed during the years 2012 and 2015 along the area of the aforementioned focus were studied. *Phlebotomus perniciosus* females collected by both sticky and CDC light traps in 29 sampling stations located in five municipalities (Fuenlabrada, Leganés, Getafe, Parla, and Humanes de Madrid) were analyzed. Blood meal identification was carried out by amplification of a fragment of 359 bp of vertebrate cytochrome *b* gene, sequencing and comparison with sequences deposited in the GenBank^®^. Moreover, RFLP-PCR of *cyt b* gene was applied in order to further discriminate between species and, especially, between mixed blood meals. A total of 535 blood-engorged *Ph. perniciosus* collected in Leganés (*n* = 401), Fuenlabrada (*n* = 126), Parla (*n* = 4), Getafe (*n* = 3), and Humanes de Madrid (*n* = 1) were studied. In 456 of them (85.24%) the blood meal was successfully identified. Blood meal identification shows that female *Ph. perniciosus* feed predominantly on hares (*n =* 341, 63.74%), most of them collected in Leganés. Rabbit blood meals were also found but in a lower proportion (*n =* 63, 11.77%). Such results could be related to the availability and abundance of these lagomorphs within the green area located close to this town. Also, dog (*n =* 12, 2.24%), human (*n =* 11, 2.05%), horse (*n =* 8, 1.5%), cat (*n =* 7, 1.31%), wild boar (*n =* 4, 0.74%), sheep (*n =* 3, 0.56%), rhea (*n =* 2, 0.38%), and partridge bird (*n =* 1, 0.19%) blood meals were identified among all the stations. Mixed blood meals were also detected: hare/rabbit (*n =* 2, 0.37%), rabbit/turkey (*n =* 1, 0.19%) and wild boar/chicken (*n =* 1, 0.19%). These behavioral observations show evidence of the opportunistic feeding behavior of *Ph. perniciosus* female sand flies. The diversity of host-feeding choices observed in this study could be explained by the availability of the different vertebrates. On one hand, high populations of hares and rabbits are described in the studied area. On the other hand, some colonies of feral cats are present in the zone, and other vertebrates such as horse, wild boar, rhea and chicken, blood of which was detected in sand flies collected in a farm school located in the urban area of Fuenlabrada town. These results can be explained as a consequence of different variables: the preference of sand flies for a specific host, the availability and abundance of the vertebrates, and the accessibility of sand flies to the different hosts. Feeding on various hosts species indicates an opportunistic behavior of *Ph. perniciosus* and can influence *Leishmania* transmission in the area. Such information is essential for planning and developing effective strategies to control vector-borne diseases with the features of leishmaniasis.

**Ecological aspects of phlebotomine sand flies in Gran Canaria (Canary Islands, Spain) and risk of *Leishmania* transmission**

Francisco Morillas Márquez^1^, Montserrat Gállego Culleré^2,3^, M.J. Morillas Mancilla^1^, V. Diaz Saez^1^, G. Merino Espinosa^1^, Bernard Pesson^4^, C. Muñoz Batet^5,6^, V. Corpas López^1^, Joaquina Martín Sánchez^1^

^1^Dpt. Parasitología, Fac. Farmacia, Univ. Granada, Spain

^2^Lab. Parasitologia, Fac. Farmacia, Univ. Barcelona, Barcelona, Spain

^3^ISGlobal, Barcelona Ctr. Int. Health Res. (CRESIB), Hospital Clínic – Univ. Barcelona, Barcelona, Spain

^4^Strasbourg, France

^5^Servei Microbiologia, Hosp. Santa Creu i San Pau, Barcelona, Spain

^6^Institut d’Investigació Biomèdica Sant Pau, Barcelona, Spain

fmorilla@ugr.es

There is a rising interest in phlebotomine sand flies due to their role as vectors of *Leishmania* and several phleboviruses, the emergence of said infectious agents and the spread of these Diptera to regions of Europe where their presence had not been reported before. The presence of phlebotomine sand flies was not reported on the Canary archipelago until 1982, when a new sand fly species was found on the island of Gran Canaria (*Phlebotomus fortunatarum*). Subsequent studies confirmed this endemism and reported the presence of other species in low numbers. On the other hand, canine leishmaniasis cases have been described on the Canary Islands for several decades, although always in dogs that had visited the Iberian Peninsula or other endemic regions, and a human Toscana virus infection has been reported as well. Moreover, in 2005 a visceral leishmaniasis case was diagnosed in a patient who had not left Gran Canaria for at least three years. This background strongly suggests the necessity of a survey of the phlebotomine sand flies of the Canary Islands, therefore the present study was conducted on the island of Gran Canaria. Sand fly captures were conducted in September 2013 using sticky-paper and CDC-light traps. Eight sampling stations with one or two light traps (10 traps/night), and 575 sticky traps (71.2 m^2^) were placed over three days in 30 sampling stations distributed over the main bioclimatic levels of the island. Using the sticky traps, 738 sand flies were captured and identified as (sorted by decreasing abundance): *Sergentomyia fallax* (50.5%), *Se. minuta* (30.4%), *Ph. fortunatarum* (11.4%), and *Ph. sergenti* (1.6%). Forty-four specimens belonging to genus *Sergentomyia* and one belonging to *Phlebotomus* could not be identified to species due to damage. *Ph. sergenti* is reported for the first time. Taking into account the bioclimatic levels, most captures were carried out in the Thermo-Canary (73.0%), followed by the Meso-Canary (25.2%). No sand flies were captured in CDC-light traps. In conclusions: 1. The only proven vector of *L. tropica* on the island is *Ph. sergenti*, which was captured in low densities (0.17 specimens/m^2^). 2. Captures were not achieved using CDC-light traps. 3. Other sand fly species with proven vectorial roles in the Mediterranean or Morocco, such as *Ph. perniciosus*, *Ph. ariasi*, or *Ph. papatasi*, were not found in this study. 4. As a consequence, risk for the transmission of *Leishmania* on the island is very low.

Financial support: Ministerio de Economía y competitividad (Madrid, Spain), project GL2010-22368-C02-02.

**First study of phlebotomine sand flies (Diptera, Psychodidae), vectors of *Leishmania* sp., in Castelo Branco District, Central East region, Portugal**

M.L. Vilela^1^, Daniela De Pita-Pereira^2^, Thais de Araujo-Pereira^2^, J.M. Cristovão^3^, Carla Maia^3^, Leana Campino^3^, M. Magalhães^4^, Elisabeth F. Rangel^1^, Maria Odete Afonso^3^

^1^Laboratório Interdisciplinar de Vigilância Entomológica em Diptera e Hemiptera, Instituto Oswaldo Cruz, FIOCRUZ, Brasil

^2^Laboratório de Biologia Molecular e Doenças Endêmicas, Instituto Oswaldo Cruz, FIOCRUZ, Brasil

^3^Unidade de Ensino e Investigação em Parasitologia Médica (UEI PM), Global Health and Tropical Medicine (GHTM), Instituto de Higiene e Medicina Tropical, Universidade de Nova Lisboa (IHMT, UNL), Portugal

^4^Laboratório de Informação em Saúde, Instituto de Comunicação e Informação Científica e Tecnológica em Saúde, LIS/ICICT/FIOCRUZ, Brasil

danypyta@gmail.com

Portugal is an endemic region of human and canine leishmaniasis due to *Leishmania infantum*. Dogs are the main reservoir, although other animals have been found infected with this parasite. *Phlebotomus perniciosus* and *Ph. ariasi* are the proven vectors in well studied leishmaniasis foci in Portugal. However, several regions of the country have never been studied with regard to phlebotomine sand fly species, as is the case with Castelo Branco District (CBD) (39°49′19″ N, 7°29′27″ W; 409 m – altitude), in the east central region, bordering Spain, where there is 12.5% prevalence of canine leishmaniasis. This study is the first phlebotomine survey in CBD, the main objectives of which were to characterize the phlebotomine sand fly fauna, including bioecological and vector aspects. Sand fly captures were performed monthly from 25 May to 1 November, 2015, with CDC light-traps, in domestic and peridomestic biotopes for three-four consecutive nights in seven of the 11 municipalities of the CBD, including 11 parishes and 24 localities. Of the 24 localities, 13 produced sand flies. The present and visible animals within 20 m of the traps, in descending order of quantity, were chickens, ducks, geese, dogs, cats, cattle and goats, rabbits, horses, pigs, pigeons, doves, swallows, turkeys and one pheasant. The total number of captured sand flies of both sexes was 151. The relative abundance of each species found was: *Ph. perniciosus* – 52.99% (80/151), *Ph. ariasi* – 25.16% (38/151), *Ph. sergenti* – 21.19% (32/151) and *Sergentomyia minuta* – 0.66% (1/151). For females, the numbers gravid and engorged were recorded as well as monthly densities, and for males, the degree of rotation of the external genitalia of males was noted. The bodies of females morphologically identified and preserved, minus genitalia, will be screened for *L. infantum* infection by kDNA PCR. Blood meal identification will be accomplished by cytochrome *b* PCR and sequencing. To characterize areas of greater risk due to the presence of *Leishmania* vectors, demographic, climatic and phlebotomine databases will be integrated in Geographic Information Systems (GIS) to produce risk maps. Preliminary results confirm that four of the five sand fly species known to occur in Portugal are present in CBD. Of these, *Ph. sergenti* appears to be spreading northward, and *Ph. perniciosus* and *Ph. ariasi* are the most abundant.

Financial support: Programa Ciências sem Fronteiras, CNPq, Brasil.

Acknowledgements: To IOC, FIOCRUZ, Brasil and UEI PM, IHMT, GHTM/FCT, UNL, Portugal, to the Presidents of the Parishes and the Staff (Sertã, Vila de Rei, and Fundada) and to the Population of the different Localities, without whom it would not have been possible to perform the phlebotomine captures.

**Phlebotomine sand fly species distribution in Croatia and implications in *Leishmania* transmission**

Sanja Bosnić^1^, Gioia Bongiorno^2^, Cristina Khoury^2^, Trentina Di Muccio^2^, Luigi Gradoni^2^, Marina Gramiccia^2^, Michele Maroli^2^

^1^Croatian Veterinary Institute, Laboratory of Parasitology, Zagreb, Croatia

^2^Unit of Vector-borne Diseases & International Health, Istituto Superiore di Sanità, Rome, Italy

gioia.bongiorno@iss.it

Leishmaniasis was reported as endemic in Croatia as early as 1930, but it is only since the early 2000s that human (both visceral and cutaneous) and canine leishmaniasis (CanL) foci have been well documented from coastal and insular territories of central and southern Dalmatia. *Leishmania* isolates from infected dogs were identified as belonging to the *Leishmania infantum* zymodeme (Z) MON-1, the widespread agent of Mediterranean zoonotic visceral leishmaniasis. We report on a 2005–2011 phlebotomine survey conducted to confirm species composition and seasonality in three central-southern counties of Dalmatia previously investigated (Šibenik-Knin, Split-Dalmatia and Dubrovnik–Neretva); to assess the current species distribution in the westernmost Istria county, including the major part of the Istrian peninsula, for which available information dates back 60 years; and to search for natural *Leishmania* infections. Further, we performed *Leishmania* sp. identification from CanL cases originating from the same counties and diagnosed during 2000–2005. Sand fly collections, carried out in the frame of bluetongue disease surveillance, used black-light suction traps employed for *Culicoides* monitoring. Fifteen localities in four Croatian counties were investigated. Specimens were preserved in ethanol pending morphological identification and DNA extraction. Canine *Leishmania* strains were identified by Multi Locus Enzyme Electrophoresis. Sand flies were trapped from late May through early December. One thousand specimens were collected and seven species identified. Among *Phlebotomus* sand flies, *Phlebotomus perfiliewi* was the most abundant species (54.6%), followed by *Ph. neglectus* (28.2%), *Ph. tobbi* (8.9%), *Ph. perniciosus* (5.4%), *Ph. papatasi* (0.6%) and *Ph. mascittii* (0.1%). *Sergentomyia minuta* accounted for 2.2%. A difference in prevalence distribution was detected, with *Ph. perniciosus* most prevalent in Istrian peninsula (49/56, 87.5%), *Ph. perfiliewi* in central counties (542/830, 65.3%) and *Ph. neglectus* in the southernmost county (79/114, 69.3%). A subset of 369 *Larroussius* females (76.2% *Ph. perfiliewi*) organized in pools (1–27 specimens/pool) according to species, site and date of collection, was analysed for *Leishmania* DNA presence. All pools were found negative. A total of 18 canine *Leishmania* strains was isolated and identified as belonging to three zymodemes of *L. infantum*: 15 strains belonged to ZMON-1, the commonest *L. infantum* zymodeme, two to ZMON-27 and one to ZMON-34, the latter two being uncommon viscerotropic and dermotropic zymodemes found in other Mediterranean countries. The long-lasting sand fly season (May-December) and the high *Ph. perniciosus* prevalence, the main *L. infantum* vector, in the westernmost Istriacounty may represent a warning signal of re-emerging leishmaniasis. *Leishmania infantum* was confirmed as the CanL agent in Croatia.

Financial support: Partially by the FP7-UE EDENext collaborative project, Contract Number: 261504.

**Sand flies (Diptera: Psychodidae) of Mediterranean Africa: inventory and distribution**

Adel Rhim^1^, Youmna M’Ghirbi^1^, Jacques Brunhes^2^, Ali Bouattour^1^

^1^Université Tunis El Manar, Institut Pasteur de Tunis, Service d’entomologie médicale, Tunisie

^2^IRD, Montpellier, France

adel.rhaim@pasteur.rns.tn

Sand flies are very small bloodsucking Diptera belonging to the suborder Nematocera and family Psychodidae. They are distributed worldwide but scarce in North America and Australia and absent in the Nordic countries. They are most common in tropical and subtropical areas of Africa, South America, Europe and Asia. *Phlebotomus* and *Sergentomyia* species are widely distributed in Mediterranean African countries where they may be involved in the transmission agents of several human and animal diseases. However, environmental changes may influence the composition of the sand fly fauna.in a particular area. For this reason it seemed useful to update the list of sand flies and their distribution in North African countries (Tunisia, Morocco, Algeria, Libya and Egypt). To develop software for the identification of sand flies of the Mediterranean Africa region, it was necessary to update the list of these insects and their distributions. We based our efforts on bibliographic data available in the international literature, with a focus on information pertaining to the presence sand fly species in the studied region, taking into account all recent publications including results of recent investigations made by our laboratory (Institute Pasteur of Tunis). The result is a list of 36 species of sand flies belonging to two genera: *Phlebotomus* (18 species comprising the five subgenra: *Paraphlebotomus*, *Adlerius*, *Larroussius*, *Phlebotomus*, and *Transphlebotomus*) and *Sergentomyia* (18 species comprising four subgenera: *Sintonius*, *Parrotomyia*, *Sergentomyia*, *Grassomyia*). Furthermore, the most recent work has reported for the first time the presence of a female of *Phlebotomus* (*Larroussius*) *chadlii* Rioux, Juminer and Gibily 1966 in Tunisia and Algeria, as well as a new record of *Phlebotomus* (*Paraphlebotomus*) *riouxi* Dépaquit, Léger, Killick-Kendrick, 1988 in Tunisia. The latter species is morphologically very close to *Ph.* (*Par.*) *chabaudi* Croset, Abonnenc, Rioux 1970 and, in fact, does not show phylogenetic or biological characteristics that would distinguish it from *Ph. chabaudi*, and therefore is considered synonymous. Finally, a confirmation of the presence in Tunisia of *Se.* (*Sin.*) *clydei* Sinton, 1928, was reported after being absent for several years. A female of *Ph.* (*Lar.*) *langeroni* Nitzeluscu, 1930 has been reported in Tunisia; which also occurs in Egypt. Similarly, after a recent inventory of sand flies in Libya, *Se. fallax* and *Se. antennata* were also reported.

**An entomological survey for sand flies in two counties of Taiwan**

Chizu Sanjoba^1^, Yusuf Ozbel^2^, Jiamei Sun^1^, Mehmet Karakus^2^, Kwang-Poo Chang^3^, Chi-Wei Tsai^4^, Tai-Chuan Wang^4^, Yoshitsugu Matsumoto^1^

^1^Department of Molecular Immunology School of Agricultural and Life Sciences, University of Tokyo, Tokyo, Japan

^2^Department of Parasitology Ege University of Faculty of Medicine, Bornova, Izmir, Turkey

^3^Department of Microbiology/Immunology, Chicago Medical School/Rosalind Franklin University of Medicine and Science, North Chicago, IL 60064, USA

^4^Department of Entomology, National Taiwan University Taipei, Taiwan

yusuf.ozbel@ege.edu.tr

The first occurrence of phlebotomine sand flies in Taiwan was noted in Tatung Township of I-lan County in 1940. In 1966, sand fly collections were carried out in 19 townships in six Counties of Taiwan. Among 1,558 specimens collected, eight species were detected but only four of them could identified as follows: *Phlebotomus kiangsuensis*, *Sergentomyia iyengari taiwanensis*, *Se. barraudi*, *Se. squamipleuris*. A morphological identification key for Taiwanese sand flies was also prepared and published in 1970. In another study completed in 1996, 979 sand fly specimens were collected from 16 townships of nine counties and the same *Sergentomyia* species were found while no *Phlebotomus* was caught. In the latest study carried out in Fushin Township of Taoyuan County, 102 specimens were collected and only *Se. iyengari taiwanensis* was found. Researchers also checked the females for the presence of *Leishmania* parasites but none of them were positive. Besides sand fly studies, three indigenous cutaneous leishmaniasis cases were reported from different counties in 2008 and 2009. In the present study, we aimed to carry out an entomological survey for morphological and molecular identification of sand fly species in the areas where leishmaniasis cases were reported previously. The sand fly collection was done in the Liquid area of Kaohsiung and Fushin county of Taoyuang between 11 and 16 September 2014 using CDC Light traps, mouth aspirators and sticky papers coated with castor oil. These areas were chosen because of their favorable topology and climate as habitats of sand flies and because of the report of patients at the site of collection in Taoyuang. The identification was done based on morphological characters, as well as molecular techniques using the cytochrome *b* gene region. The female specimens were first kept in Marc André solution overnight for clarification and after separating the head from the body, slides were prepared for morphological identification. Male specimens were directly mounted after separating the head from the body. The DNA isolation was done using three legs of the specimens. A total of 66 sand fly specimens (36 male & 30 female) was collected in five nights of trapping. The preliminary results of morphological and molecular identification showed that one *Phlebotomus* species, *Ph. kiangsuensis*, and three *Sergentomyia* species are present in the study areas. The identification studies are still ongoing. In conclusion, because Taiwan has a tropical climate and many places are suitable for breeding of sand flies, the studies related to sand fly fauna and natural *Leishmania* infection need to be continued in “endemic” areas of Taiwan

**An inventory of phlebotomine sand flies from Cambodia**

Thibault Vallecillo^1^, Eva Krupa^1^, Julian Gratiaux^1^, Idiyana Rahima Abdou el Aziz^1,2^, Kimsour Kang^2^, Kalian Ouk^3^, Mathieu Loyer^1^, Frédérick Gay^4^, Arezki Izri^2^, Jérôme Depaquit^1^

^1^Université de Reims Champagne Ardenne, ANSES, SFR Cap santé, EA 4688 – USC « Transmission Vectorielle et Épidémiosurveillance de Maladies Parasitaires (VECPAR) », Reims, France

^2^Parasitologie-Mycologie, CHU Avicenne, Université Paris 13, Bobigny, UMR 190, Unité des Virus Émergent, Marseille, France

^3^Université Chenla, Phnom Penh, Cambodia

^4^Université Pierre et Marie Curie-Paris 6, CHU Pitié-Salpêtrière, AP-HP, Groupe Hospitalier Pitié-Salpêtrière, Service Parasitologie-Mycologie, Paris, France

thibault.vallecillo@gmail.com

The sand fly fauna of Cambodia remains poorly documented. Because of the lack of local *Leishmania* transmission, very few studies related to Cambodian phlebotomine sand flies are available in the literature. Only six species of sand flies have previously been recorded: *Sergentomyia barraudi*, *Se. pertubans*, *Se. bailyi*, *Se. sylvatica*, *Se. khawi* and *Grassomyia indica*. None belong to the genus *Phlebotomus*. However, in connection with the emergence of the first autochthonous cases in Thailand and Vietnam, we carried out an inventory of the sand flies from the country. Sand flies were sampled using CDC miniature light traps in different places in the country including two main biotopes: villages with animals and caves. Specimens were identified according to their original descriptions. In addition, DNA barcoding based on cytb sequences was performed. The identification of males of the genus *Sergentomyia* has not been done yet due to difficulty in identifying them. We identified 10 species belonging to four genera: *Idiophlebotomus nicolegerae*, *Phlebotomus stantoni*, *Ph. kiangsuensis*, *Se. barraudi* s.l., *Se. bailyi*, *Se. sylvatica*, *Se. khawi*, *Se. anodontis*, *Se. hibernus* and *Gr. indica*. The molecular data are in agreement with the morphological identifications. The status of *Sergentomyia barraudi* s.l. and *Se. khawi* must be carefully examined taking into account both morphology and the division of each species in two molecular populations. Finally, we recorded five species new to the fauna of Cambodia.

## Laboratory studies (oral communications)

**Keynote – The unparalleled efficiency of *Leishmania* transmission by sand fly bites**

Ranadhir Dey^1^, Vanessa Atayde^2^, Amritanshu Joshi^1^, Hamide Aslan^3,4^, Lais da Silva^3^, Shannon Townsend^3^, Claudio Meneses^3^, Hira Nakhasi^1^, Martin Olivier^2^, Jesus Valenzuela^3^, Shaden Kamhawi^3^

^1^Laboratory of Emerging Pathogens, Center for Biologics Evaluation and Research, Food and Drug Administration, Silver Spring, MD 20993, USA

^2^Infectious Diseases and Immunity in Global Heath Program, Research Institute of the McGill University Health Centre, 1001 Boulevard Decarie, Montreal, QC H4A 3J1, Canada

^3^Vector Molecular Biology Section, Laboratory of Malaria and Vector Research, National Institute of Allergy and Infectious Diseases, National Institutes of Health, Rockville, Maryland 20852, USA

^4^Faculty of Health Science, Selahaddin Eyyubi University, Diyarbakir, Turkey

skamhawi@niaid.nih.gov

Transmission of *Leishmania* parasites by vector sand fly bites continues to fascinate us with its complexity. To date, various researchers have demonstrated that sand flies do not simply inject *Leishmania* parasites into the skin. Instead, the infectious inoculum includes various vector-derived factors that are equally vital for early parasite survival and establishment, enhancing virulence of leishmaniasis. This heightened virulence has been attributed to the immunomodulatory properties of vector-derived factors, the most renowned being saliva and the promastigote secretory gel (PSG). Here, I report on two newly identified vector-derived factors, *Leishmania* exosomes and sand fly gut microbiota, that contribute to the unique inflammatory environment favoring parasite survival at the bite site. Our studies show that exosomes are secreted in abundance by parasites developing in the midgut. These exosomes accumulate within the gut lumen and are egested into the mammalian host where they enhance the virulence of *Leishmania* parasites. Gut microbiota are also egested into the mammalian host triggering a distinctive inflammatory response dominated by activation of the inflammasome and IL1β production. The high level of IL1β at the bite site drives persistent recruitment of neutrophils and is essential for successful visceralization of *L. donovani* parasites. It is clear that the collective specific and shared effects of vector-derived factors continue to define the outcome of *Leishmania* transmission by vector bite rendering it unparalleled in efficiency and impossible to reproduce in its entirety.

**A comparison of vector competence in different sand fly species to transmit *Leishmania donovani***

Jovana Sadlova^1^, Jitka Myskova^1^, Katerina Pruzinova^1^, K. Homola^1^, M. Yeo^2^, Petr Volf^1^

^1^Department of Parasitology, Faculty of Science, Charles University, Prague, Czech Republic

^2^Department of Pathogen Molecular Biology, Faculty of Infectious and Tropical Diseases, London School of Hygiene and Tropical Medicine, London, UK

sadlovaj@natur.cuni.cz

Development of *Leishmania* infections in sand fly vectors is a complex, often species-specific process. Factors controlling vector competence during the early phase of infection are closely associated with bloodmeal digestion where *Leishmania* are exposed to midgut digestive enzymes and persistence of an acellular chitin-containing envelope (the peritrophic matrix, PM). After escape from the PM parasites attach to the midgut epithelium enabling them to avoid expulsion from the midgut with remnants of the digested bloodmeal during defecation. The aim of our study was to assess the influence of the PM and bloodmeal digestion on the course of *L. donovani* infections in both refractory and susceptible sand fly’s by measuring key developmental parameters of infection. We compared development of *L. donovani* in two susceptible sand fly species (*Phlebotomus orientalis*, *Ph. argentipes*) and two refractory ones (*Ph. papatasi*, *Sergentomyia schwetzi*). Amastigote-initiated infections were performed using a membrane feeding method; parasite numbers, their physical location *in situ*, presence and proportion of morphological forms were all examined by fluorescence microscopy at 2, 3, 6 and 10 days post bloodmeal ingestion. In addition, we studied various parameters of bloodmeal digestion. These included volume of ingested bloodmeal, time to defecation of bloodmeal remnants, time of formation and degradation of the peritrophic matrix (PM) and its morphology, and finally trypsin and chymotrypsin enzyme activity in the midgut. Parasites were also exposed to midgut lysates *in-vitro* to further investigate their effects on *Leishmania* differentiation from amastigote to promastigote morphological forms. Parasites produced massive infections with colonization of the stomodeal valve and presence of infective metacyclic stages in susceptible *Ph. orientalis* and *Ph. argentipes* vector species. These two natural vectors showed significantly lower trypsin activity and relatively slower formation of the PM than refractory species. In contrast, *L. donovani* infections were not maintained and did not progress past defecation of blood remnants in either refractory vector species (*Se. schwetzi* and *Ph. papatasi*). With regard to *Se. schwetzi*, parasites remained enclosed within the PM, whereas in *Ph. papatasi* parasites reached the abdominal midgut. The loss of infections in both refractory sand fly species is, therefore, caused by different mechanisms. It was apparent for *Se. schwetzi*, that the time period between degradation of the PM and defecation of bloodmeal remnants was extremely short, promastigotes were unable to escape from the PM before defecation. In contrast, in *Ph. papatasi*, infections survived high activities of midgut proteases and escaped from the PM, but failed to attach to the midgut epithelium and so were ejected during defecation. Procyclic promastigotes remained the prevailing morphological form in both refractory species. The findings provide insight into factors affecting *Leishmania* development and help predict vector competency of different sand fly species.

***Leishmania donovani* in *Phlebotomus argentipes*: comparison of development and transmission of amastigote- and promastigote-initiated infections**

Tereza Lestinova^1^, Jovana Sadlova^1^, Jitka Myskova^1^, Jan Votypka^1^, V. Yeo^2^, Petr Volf^1^

^1^Department of Parasitology, Faculty of Science, Charles University, Prague, Czech Republic

^2^Department of Pathogen Molecular Biology, Faculty of Infectious and Tropical Diseases, London School of Hygiene and Tropical Medicine, London, UK

terka.kratochvilova@seznam.cz

All *Leishmania* species share a digenetic life cycle characterized by motile promastigote stages that develop in the gut of phlebotomine sand flies, and by non-motile amastigotes residing inside mononuclear phagocytic cells of vertebrate hosts. Under laboratory conditions, both aforementioned *Leishmania* stages can initiate experimental infections of sand flies. While promastigotes can be simply cultivated *in vitro*, ingestion of promastigote-initiated forms by sand fly females is unnatural. In contrast, amastigote-initiated infections are natural, but associated with ethical concerns and some other disadvantages, which make their usage unfavorable. Assessing amastigote dose by direct feeding on infected hosts is difficult and those derived from organs of infected animals requires frequent animal sacrifice and are unavoidably contaminated with host material. Alternatively, cultivation of axenic amastigote-like forms is relatively easy, however, a large number of studies infer considerable differences when comparing axenic amastigotes with “real” macrophage- or lesion-derived amastigotes. For these reasons, the cultivation of amastigotes via macrophages, or macrophage-like cell lines remains the gold standard although it is relatively time-consuming and laborious. In our study, we address an important omission in the current knowledge of experimental *Leishmania* infections in sand flies, namely the extent to which promastigote-initiated experimental infections differ from those initiated with amastigotes. We compared the development of promastigote- against amastigote-initiated *L. donovani* infections in the natural vector *Phlebotomus argentipes* and, for the first time, the subsequent effect on transmission to BALB/c mice*.* Promastigotes from log-phase cultures and amastigotes grown *ex vivo* in macrophages, derived from bone marrow of mice, were mixed with heat-inactivated rabbit blood (10^6^ parasites per 1 mL of blood) and *Ph. argentipes* infected by feeding through chick-skin membranes. Infections initiated with promastigotes had a markedly quicker onset in *Ph. argentipes.* Ingested amastigotes underwent a substantial initial reduction in numbers before they start to multiply. As expected, in the early phase of infection (day 1-2 post bloodmeal) experimental groups differed in representation of morphological forms; procyclic promastigotes prevailed in amastigote-initiated infection while short promastigotes were dominant in promastigote-initiated infection. More importantly, in mature infections (day 8-9 post bloodmeal), no significant differences were observed either in intensity of infection, *in situ* distribution of parasites, or in numbers of metacyclic forms. The efficiency of *Leishmania* transmission, the most important marker of successful parasite development in the vector, was also comparable between both experimental groups. We conclude that use of promastigote stages for sand fly infections does not alter the final outcome of *L. donovani* development in *Ph. argentipes*. Nevertheless, for studies specific to early phase of *Leishmania* development in sand flies, i.e. before defecation of blood remnants, amastigotes grown in macrophages should be used.

**Establishing, expanding and certifying a closed working colony of *Phlebotomus argentipes* (Diptera: Psychodidae) for xenodiagnostic studies at the kala azar medical research center, Muzaffarpur, Bihar, India**

Puja Tiwary^1^, Shakti Kumar Singh^1^, O.P. Singh^1^, David Sacks^2^, Shyam Sundar^1^, Edgar Rowton^3^, Phillip Lawyer^2^

^1^Department of Medicine, Institute of Medical Sciences, Banaras Hindu University, Varanasi, Uttar Pradesh, India

^2^Laboratory of Parasitic Diseases, National Institute of Allergy and Infectious Diseases, National Institutes of Health, Bethesda, Maryland, USA

^3^Headquarters, Walter Reed Army Institute of Research, Silver Spring, Maryland, USA

plawyer349@verizon.net

The Kala Azar Medical Research Center (KAMRC), Muzaffarpur, Bihar, India is a field station of the Institute of Medical Sciences, Banaras Hindu University. This pilot project is preliminary and essential to a larger follow-on effort aimed at defining the ability of specific human-subject groups across the infection spectrum to serve as reservoirs of *Leishmania donovani* infection to sand flies in areas of anthroponotic transmission such as Bihar state. This is possible only via xenodiagnosis of well-defined subject groups using live vector sand flies. The objective was to establish, at the KAMRC, a robust, self-sustaining, working colony of *Phlebotomus argentipes* Annandale and Brunetti, closed to infusion with wild-caught material and certified safe for human xenodiagnostic use. Prior to this endeavor, no laboratory colony of this vector existed in India that met the stringent biosafety requirements of this human-use study. Requisite for initiating and establishing a permanent sand fly colony were: construction of a proper insectary facility, procurement of equipment and supplies, as well as training of personnel to perform the complex, labor-intensive procedures required to support the colony. All this was accomplished in seven months, March to September, 2014, under a pilot grant from the Bill and Melinda Gates Foundation. From September through mid-December, 2014, sand flies were collected from VL-endemic regions of Muzaffarpur district, Bihar (26.07uN 85.45uE) in rural villages selected previously based on reported VL cases. As a result of this first effort, a small colony was initiated and maintained for three generations but it did not achieve the critical mass necessary to be self-sustaining before the end of the collecting season. Then in March, 2015, a village was identified in which residual spraying had not been done recently and where sand fly density was consistently high enough to enable trapping large numbers of sand flies to build the colony. From March through mid-December, 2015, sand flies were collected in human dwellings and cattle sheds using 30 light traps over 254 nights (7,620 trap nights). A total of 68,601 flies were collected (37,397 males; 31,204 females). From 13,348 females set up in isoline vials for oviposition, 2,598 clutches averaging 28 eggs each were harvested, approximately 90% of which hatched). Progeny were reared according to standard methods, providing a continuous critical mass of F1 males and females to stimulate optimal social feeding behavior. With the construction of a large feeding cage and use of a unique, custom-made rabbit restrainer, the desired level of blood-feeding on un-anesthetized rabbits was achieved for the colony to be self-sustaining and expanded to working level. Presently in its 10th generation, the colony yields 1,500–2,500 blood-fed females per week for egg production. Because the colonized sand flies will be used for xenodiagnosis on humans, the colony was closed to further infusion with wild-caught material in December, 2015, and steps were taken via PCR to insure the purity of the colony as *Ph. argentipes* and to certify it free of pathogens of potential or actual concern to humans.

***Leishmania tropica* development in *Phlebotomus sergenti*: the effect of temperature, gregarines and geographic origin of sand flies**

Jana Hlavacova, Magdalena Jancarova, Jan Votypka, Petr Volf

Department of Parasitology, Faculty of Science, Charles University in Prague, Vinicna 7, 128 43 Prague 2, Czech Republic

janehlavac@seznam.cz

*Phlebotomus sergenti* Parrot, 1917 is a widely distributed Old World species recognized as the main vector of *Leishmania tropica*. It also serves as a host of gregarine *Psychodiella sergenti*, insect parasite specific for this sand fly species. In mosquitoes, it was shown that gregarines can affect development of other parasites, like microsporidia or arboviruses. Therefore, we studied the effect of co-infection of gregarine *Psychodiella sergenti* on *L. tropica* in *Ph. sergenti*. We found that *Leishmania* developed similarly well in both sand fly groups, causing the same rate of heavy late stage infections in gregarine-infected and non-infected sand flies. Next, we studied the impact of larval rearing temperature (27 and 32 °C) on *Ph. sergenti* susceptibility to *L. tropica* and gregarine *Psychodiella sergenti*. Conditions experienced during larval development are known to affect fitness of adults in many insect species. It was previously shown that rearing temperature of larvae can affect susceptibility of mosquitoes to arboviruses. However, no analogous studies have ever been performed on sand flies. Here, we demonstrated that temperature significantly affects development of the gregarine *Ps. sergenti* in *Ph. sergenti*. Fourth instar larvae maintained at 32 °C were significantly less infected by gregarines than the control group maintained at 27 °C. In adults, the difference between two groups tested was even more pronounced: All 117 adults emerged from larvae kept at 27 °C gregarine positive, but only three of 120 adults from the group kept at 32 °C were gregarine positive. In contrast, *L. tropica* thrived comparably well in females obtained from both temperatures tested; no differences were observed either in rates of infected sand flies or in intensity of *Leishmania* infection. Additionally, we observed that larvae kept at higher temperatures develop faster and produce smaller adults. We hypothesize that higher temperature tested may modulate immune response which, together with faster development of sand flies, could intensify loss of gregarines. Finally, as *Ph. sergenti* has a broad geographic distribution and molecular heterogeneity, which were previously suggested to affect vector competence to *L. tropica*, we infected laboratory-reared *Ph. sergenti* originating from Turkey and Israel to compare their susceptibility*.* In both tested groups, *L. tropica* developed equally well, causing heavy late-stage infections with the colonization of the stomodeal valve. This indicates that the different geographic origins of *Ph. sergenti* are not reflected by a different vector competence to *L. tropica*.

***Lutzomyia umbratilis* population captured in the south of the Negro River is refractory to interaction with *Leishmania guyanensis***

R.P. Soares^1^, P.M. Nogueira^1^, N.F.C. Secundino^1^, E.F. Santos^2^, C.M. Ríos-Velásquez^2^, F.A. Pessoa^2^

^1^Centro De Pesquisas René Rachou/Fundação Oswaldo Cruz, Belo Horizonte – MG, Brasil

^2^Centro de Pesquisas Leônidas e Maria Deane/Fiocruz, Manaus – AM, Brazil

rsoares@cpqrr.fiocruz.br

*Lutzomyia umbratilis* is the vector of *Leishmania guyanensis* in Northern South America. This vector has been found naturally infected with this parasite only east of the Negro River and north of the Amazonas River. However, populations of *Lu. umbratilis* are present also in areas south of these rivers, which are natural geographic barriers. An interesting aspect is that this sand fly species has never been found infected or to be transmitting *L. guyanensis* in these areas. Genetic differences among *Lu. umbratilis* populations suggest that they may be in the process of speciation. However, no studies of the parasite-host interactions are available. Here, the interaction of *L. guyanensis* (MHOM/BR/75/M4147) with *Lu. umbratilis* captured at two sites, Rio Preto da Eva and Manacapuru (north and south of the Negro River, respectively), was evaluated. Procyclic and metacyclic attachment was quantitated *in vitro* after interaction with the midguts of field-collected *Lu. umbratilis*. Midguts (11 per group) were incubated for 20 min with procyclic and metacyclic promastigotes (2 × 10^7^ cells/mL) and the number of attached parasites counted. No attachment of parasites was observed in the midguts of any of the insects from Manacapuru. On the other hand, a high binding of PNA+ parasites was observed in the midguts of flies from Rio Preto da Eva and this attachment was more pronounced than that observed for PNA- (*P* < 0.001). These data suggest that the population of *Lu. umbratilis* south of the Negro River is refractory to interaction with *L. guyanensis*, corroborating previous epidemiological information on *L. guyanensis* transmission in different regions of the Amazon.

Financial support: CNPq and FAPEMIG.

***Lutzomyia migonei* is a permissive vector competent for *Leishmania infantum***

Katerina Pruzinova^1^, Vanessa Cristina Fitipaldi Veloso Guimarães^2^, Jovana Sadlova^1^, Vera Volfova^1^, Sinval Pinto Brandão Filho^2^, Petr Volf^1^

^1^Department of Parasitology, Faculty of Science, Charles University, Prague, Czech Republic

^2^Department of Immunology, Centro de Pesquisas Aggeu Magalhães, Fundação Oswaldo Cruz (Fiocruz), Recife, Pernambuco, Brazil

katerina.pruzinova@gmail.com

In Latin America, *Leishmania infantum* is primarily transmitted by *Lutzomyia longipalpis*, but the role of *Lu. migonei* as a potential vector has been repeatedly discussed. *Lu. migonei* is well known for its opportunistic feeding habits and anthropophilic behaviour and repeatedly has been found to be naturally infected by *L. infantum*. The vectorial role of this sand fly species has been suggested especially in areas with a record of human and canine cases of visceral leishmaniasis but where the proven vector is absent. However, the vector competence of *L*. *migonei* has not yet been proven experimentally. In our study, we evaluated for the first time the susceptibility of *Lu*. *migonei* to *L*. *infantum* and used *Lu. longipalpis* as a positive control. Females of laboratory-reared *Lu. migonei* and *Lu. longipalpis* were fed through a chick-skin membrane on heat-inactivated rabbit blood containing 10^6^ promastigotes/mL. A viscerotropic *L. infantum* strain (MHOM/BR/76/M4192) and dermotropic *L. infantum* strain (ITOB/TR/2005/CUK3) were used in the experiments. Blood-fed females were dissected at one, five and eight days post-infection (PI) and checked microscopically for the presence, intensity and localization of *Leishmania* infections. In addition, morphological forms of promastigotes were analysed. Both *L. infantum* strains developed well in *Lu. migonei* with high infection rates and intensities of infections. *Leishmania* colonized the cardia region and stomodeal valve in most *Lu. migonei* females from day 5 PI and no significant differences were found in comparison with the development in *Lu. longipalpis.* Metacyclic forms were observed in both sand fly species. Our study clearly demonstrated that *L*. *infantum* develops late-stage infections in *Lu*. *migonei*, fully comparable to those found in *Lu. longipalpis.* This, together with its known anthropophily, abundance in VL foci and natural infection by *L*. *infantum*, constitute important evidence that *Lu*. *migonei* is a vector of this parasite in Latin America. Since it has been reported that *Lu. migonei* supports the development of other *Leishmania* species, namely *L. braziliensis* and *L. amazonensis*, we propose that this sand fly species is a permissive vector susceptible to various *Leishmania* and *Viannia* species. Our results contribute to a better understanding of the epidemiology of VL caused by *L. infantum* in South America.

***Leishmania* proteophosphoglycans regurgitated from infected sand flies accelerates dermal wound repair and exacerbates leishmaniasis via insulin-like growth factor 1-dependent signalling**

Emilie Giraud^1^, Tamsyn Derrick^1^, Oihane Martin^1^, Rod J. Dillon^2^, Tereza Leštinová^3^, Petr Volf^3^, Ingrid Műller^4^, Paul A. Bates^2^, Matthew E. Rogers^1^

^1^Faculty of Infectious and Tropical Diseases, London School of Hygiene and Tropical Medicine, WC1E 7HT, UK

^2^Division of Biomedical and Life Sciences, Lancaster University, LA1 4YB, UK

^3^Department of Parasitology, Charles University, Vinicna 7, Prague 2, 128 44 Czech Republic

^4^Department of Medicine, Section of Immunology, Imperial College London, W2 1PG, UK

emilie.Giraud@lshtm.ac.uk

The promastigote secretory gel (PSG) is a matrix of filamentous proteophosphoglycan secreted by *Leishmania* promastigotes inside the sand fly gut, which facilitates the transmission and infection of the mammalian host. The early host response to PSG has not been characterised. One thousand *Leishmania mexicana* metacyclic promastigotes were inoculated into BALB/c mouse ears, with or without PSG. The Affymetrix Mouse GeneChip revealed differential expression of 7,927 transcripts (FC > 1.5, 5% FDR) to PSG, i.e. 27% of the mouse genome. We found that PSG was associated with an early up-regulation of transcripts involved in inflammation, inflammatory cell recruitment, epithelial cell proliferation and fibrosis. *In vitro-* and *in vivo-*experiments revealed that PSG significantly accelerated wound healing. Insulin-like growth factor 1 (IGF1) is linked to macrophage alternative activation and wound repair. Dermal expression of IGF1 was enhanced following an infected sand fly bite and was acutely responsive to the PSG but not to parasites or sand fly saliva. Antibody blockade of IGF1 ablated the gel’s ability to promote wound closure in mice and significantly reduced the virulence of *L. mexicana* infection delivered by sand fly bite. These results show that PSG strongly influences multiple stages of the wound healing process in skin following *Leishmania* transmission; resulting in accelerated healing and, via IGF1-signalling, provides an environment that promotes parasite survival and growth.

**Attraction of *Lutzomyia* sp. (Diptera: Psychodidae: phlebotomine) to volatile organic compounds from the skin odour of individuals residing in an endemic area for tegumentary leishmaniasis**

D.S. Tavares^1^, P.R.R. Mesquita^3^, V.R. Salgado^2^, F.M. Rodrigues^3^, J.C. Miranda^2^, A. Barral^2^

^1^Post-graduation Program of Pathology, Faculty of Medicine, Federal University of Bahia, Oswaldo Cruz Foundation, Fiocruz, Salvador, Bahia, Brazil

^2^Laboratory of Immunoparasitology, Center of Researches Gonaçalo Moniz, Oswaldo Cruz Foundation – Fiocruz, Rua Waldemar Falcão, 121 – Candeal, 40296-710 Salvador, Bahia

^3^Chemistry Institute, Department of General and Inorganic Chemistry, Federal University of Bahia, Brazil

divast.bio@gmail.com

The olfactory sense of insects is the most important tool for their orientation. Hence, blood-feeding insects find their hosts by following olfactory cues. Female phlebotomine sand flies feed on vertebrate hosts to obtain nutrients from blood required to mature their eggs. Some species are competent vectors of *Leishmania* and infect humans via their bites. However, little is known about what attracts these insects to humans. Some workers have investigated this question for mosquitoes of medical importance, like *Anopheles* sp. and *Aedes* sp., but very few studies have been developed with phlebotomine sand flies. Volatile organic compounds (VOCs) present in human odours have been shown to represent important cues for insects, when seeking their sources for blood meal. Octenol, for instance, is an alcohol present in human sweat and it has been shown to enhance attraction of *Lutzomyia neivai*, *L. intermedia* and *Lu. longipalpis* under both field and laboratory conditions. Heptanol, octanol and nonanol are also present in human sweat and have been shown to attract *Lu. longipalpis* and *Lu. neivai* in wind tunnel assays. The main objective of this study was to evaluate phlebotomine sand flies’ attraction to VOCs from human skin odour of male individuals residing in an area endemic for tegumentary leishmaniasis. To accomplish this, 33 individuals, from Corte de Pedra, Southeast of Bahia, Brazil, between 18 and 60 years of age, were invited to participate by allowing collection of hair from their legs, after it had retained odours from the skin. Headspace Solid Phase Microextraction (HS-SPME) was applied associated with Gas Chromatography and Mass Spectrometry (GC-MS) for VOCs extraction and detection, respectively. Females of *Lutzomyia* sp. were captured in the field with automatic light traps, and subjected to attraction behaviour bioassays in a transparent acrylic wind tunnel. For each test, three female *Lutzomyia* sp. were placed in a releasing chamber for a 20 min-acclimatation, in the dark and with no food sources. The chamber was then placed 110 cm downwind from the odour source. Each tested VOC was delivered (200 μL) on a filter paper (4 × 4 cm). The trials were 2 min-long and activation and attraction behavior were recorded. At least 10 replicates were conducted for each VOC tested. Forty-two VOCs were identified and until now six of them (phenylacethaldehyde, tetradecane, hexadecane, eicosane, pentadecane, 6-methyl-5-hepten-2-one), plus hexane and octenol (negative and positive control, respectively) were tested through wind tunnel assays for sand fly attraction. As expected, octenol (positive control) significantly induced activation and attraction of female *Lutzomyia* sp. (*P* = 0.0237). Significant activation was also observed for phenylacethaldehyde (*P* = 0.0044), eicosane (*P* = 0.0257) and 6-methyl-5-hepten-2-one (*P* = 0.0352). Significant attraction was observed for phenylacethaldehyde (*P* = 0.0328) and eicosane (*P* = 0.0257). It has been reported that skin human odours are a product of resident bacterial metabolism. Except for eicosane, the tested VOCs are reported as products of the metabolism of bacteria from human skin. *Bacillus subtilis* is known to produce phenylacethaldehyde and hexadecane and *Streptomyces* sp. is produces phenylacethaldehyde and 6-methyl-5-hepten-2-one. Phenylacethaldehyde is also produced by *Staphylococcus* sp. The results so far are promising as a means of enhancing phlebotomine sand fly captures in the field, which may be very helpful for monitoring sand fly activity. In addition, the enhanced capture of these insects can also provide material for laboratory research on prevention and control of leishmaniasis.

**Blood derived haem as a potential elicitor of anti-leishmanial activity in the gut of the female sand fly *Lutzomyia longipalpis***

José R. Silva^1^, Emma Shawcross^2^, Rod J. Dillon^2^

^1^Lab Integrado de Bioquímica Hatisaburo Masuda, Núcleo de Pesquisas em Ecologia e Desenvolvimento Sócio-Ambiental, Universidade Federal do Rio de Janeiro, Campus Macaé, CEP: 27971-550 Macaé, Brazil

^2^Faculty of Health and Medicine, Division of Biomedical and Life Sciences, Lancaster University, LA1 4YQ Lancaster, UK

r.dillon@lancaster.ac.uk

Establishment and development of a transmissible *Leishmania* infection in the gut of the female sand fly requires that the parasite either is resistant to the anti-parasitic immune molecules present or that the molecules are not expressed at sufficient levels in the gut to cause suppression of the *Leishmania*. Our previous studies showed that reactive oxygen species (ROS) are a component of the innate immune response in the gut of phlebotomine sand flies. We suggested that sand flies tolerate the presence of *Leishmania* by differential response of the ROS system. The aim of the present study was to examine the role of blood-derived haem and the influence of the peritrophic matrix on ROS production in the gut. Feeding chitinase-supplemented blood to the sand flies resulted in increased hydrogen peroxide concentration compared to blood-fed controls. Chitinase destroys peritrophic matrix (PM) integrity and this result suggests that the PM limits ROS production by the blood during blood digestion. Initiating a *L. mexicana* infection with chitinase supplemented blood resulted in a significant decline of parasite population in the gut. In order to check the role of ROS during this infection, a group of sand flies was fed *ad libitum* following emergence on uric acid-supplemented sucrose solution prior to infection. The supplement “rescued” (restored) the *Leishmania* infection to populations similar to controls. We will describe further experiments regarding the role of haem during *Leishmania* growth within the digesting bloodmeal. Results will be discussed in the light of previous studies showing that the presence of the PM also protects *Leishmania* from proteolysis due to digestive enzymes.

Financial support: CNPq Brazil and BBSRC UK.

**Bacterial communities associated with the digestive tract of wild populations of *Lutzomyia evansi*: a vector of *Leishmania* in Colombia**

Rafael José Vivero^1,+^ ^,2,3,4^, Gloria Ester Cadavid-Restrepo^4^, Sandra I. Uribe Soto^2,3^, Claudia Ximena Moreno Herrera^4^, Ivan D. Velez^3,+^

^1^PhD Student in Biotechnology, Universidad Nacional de Colombia, Sede Medellín, Colombia

^2^Grupo de Investigación en Sistematica Molecular, Universidad Nacional de Colombia, Sede Medellín, Colombia

^3^PECET (Programa de Estudio y Control de Enfermedades Tropicales), Universidad de Antioquia, Medellín, Colombia

^4^Grupo de Microbiodiversidad y Bioprospección, Laboratorio de Biología Celular y Molecular, Universidad Nacional de Colombia, Sede Medellín, Colombia, Colombia

idvelez@pecet-colombia.org

*Lutzomyia* (*Lutzomyia*) *evansi* is a phlebotomine sand fly endemic to the Caribbean coast of Colombia with epidemiological significance as the main *Leishmania* vector in that region. Although the intestinal microbiota of these insects may include pathogenic and non-pathogenic microorganisms, there is little knowledge of the bacterial diversity present in the digestive tract of wild populations. In this study, conventional microbiological methods and molecular tools, were used to assess the composition of bacterial communities within the digestive tracts of immature and adult stages of wild *Lu. evansi* from the department of Sucre (Caribbean coast of Colombia). Different molecular techniques for the identification of bacteria were used, such as, ribosomal intergenic spacer analysis (RISA) and analysis of the 16S rRNA gene. We also detected the presence of *Wolbachia* endosymbiont bacteria and *Leishmania* parasites by PCR and DNA sequence analysis in *Lu. evansi* and other species. The culture-dependent technique showed that the intestinal bacteria belonging to *Acinetobacter*, *Enterobacter*, *Pseudomonas*, *Ochrobactrum*, *Shinella* and *Paenibacillus* were the dominant bacteria isolated in larvae; *Lysobacter*, *Microbacterium*, *Streptomyces*, *Bacillus* and *Rummeliibacillus* in pupae; and *Staphylococcus*, *Streptomyces*, *Brevibacterium*, *Acinetobacter*, *Enterobacter* and *Pantoea* in adult stages. Statistical analysis reveals that the fingerprint pattern of the PCR – TGGE bands varies significant between bacterial communities of immature and adult *Lu. evansi*. Results show 20% infection by *Wolbachia* in samples of *Lutzomyia* tested*.* Endosymbiotic *Wolbachia* were found in three species: *Lu. cayennensis* and *Lu. dubitans*, with positive pools = 3; 8.5% for both species, and *Lutzomyia evansi*, with one positive pool = 1; 2.8%. Two distinct *Wolbachia* genotypes (strains) were found, *wLev* in *Lu. dubitans*, *Lu. cayennensis*, and *Lu. evansi*; while *wLcay* was found only in *Lu. cayennensis.* The analyses by microbiological and molecular approaches revealed significant variation in the bacterial communities associated with the digestive tract of *Lu. evansi*, depending on the developmental stage and the blood intake of females. Evidence of *Wolbachia* infections in natural *Lutzomyia* populations warrant further investigation on the possible effects of this bacteria in *Leishmania* transmission.

## Systematics and phylogeny (posters)

**What we know of the classification, evolution, and dispersion of *Leishmania* parasites and sand flies?**

Mohammad Akhoundi^1^, Katrin Kuhls^2^, Arnaud Cannet^3^, Jan Votýpka^4,5^, Pierre Marty^1,3^, Pascal Delaunay^1,3^, Denis Sereno^6,7^

^1^Service de Parasitologie-Mycologie, Hôpital de l’Archet, Centre Hospitalier Universitaire de Nice, Nice, France

^2^Division of Molecular Biotechnology and Functional Genetics, Technical University of Applied Sciences Wildau, Wildau, Germany

^3^Inserm U1065, Centre Méditerranéen de Médecine Moléculaire, Université de Nice-Sophia Antipolis, Nice, France

^4^Biology Centre, Institute of Parasitology, Czech Academy of Sciences, Prague, Czech Republic

^5^Department of Parasitology, Faculty of Science, Charles University in Prague, Prague, Czech Republic

^6^MIVEGEC, UMR CNRS-IRD-Université de Montpellier Centre IRD, Montpellier, France

^7^UMR177, Centre IRD de Montpellier, Montpellier, France

m.akhoundi@yahoo.com

Leishmaniases are vector-borne diseases caused by obligate protozoan parasites of the genus *Leishmania* (Trypanosomatida: Trypanosomatidae). Leishmaniasis is transmitted by bites of infected female sand flies, whose hosts are animals such as canids, rodents, marsupials, hyraxes, or humans. The aim of our study is to describe the major evolutionary historical events among *Leishmania*, sand flies, and the associated animal reservoirs in detail, in accordance with the geographical evolution of the Earth, which has not been previously discussed on a large scale. Classification of *Leishmania* and sand flies has always been a controversial matter, and the increasing number of species currently described further complicates this issue. Despite several hypotheses on the origin, evolution, and distribution of *Leishmania* and sand flies in the Old and New Worlds, no consistent agreement exists regarding dissemination of the actors that play roles in leishmaniasis. For this purpose, we summarize here three centuries of research on sand fly and *Leishmania* descriptions, as well as a complete description of *Leishmania* and sand fly fossils and the emergence date of each *Leishmania* and sand fly group during different geographical periods, from 550 million years ago until now. We discuss critically the different approaches that were used for *Leishmana* and sand fly classification and their synonymies, proposing an updated classification for each species of *Leishmania* and sand fly. We update information on the current distribution and dispersion of different species of *Leishmania* (53), sand flies (more than 800 at genus or subgenus level), and animal reservoirs in each of the following geographical ecozones: Palearctic, Nearctic, Neotropic, Afrotropical, Oriental, Malagasy and Australian. We propose an updated list of the potential and proven sand fly vectors for each *Leishmania* species in the Old and New World. Finally, we address a classical question about digenetic *Leishmania* evolution: which was the first host, a vertebrate or an invertebrate?

**Illustrated identification key to females of Phlebotominae recorded in the Central-West Region of Brazil using only head and spermathecae**

Douglas de Almeida Rocha^1^, Eunice Aparecida Bianchi Galati^2^, Andrey José de Andrade^3,4^

^1^Núcleo de Medicina Tropical, Universidade de Brasília, Brasil

^2^Departamento de Epidemiologia, Faculdade de Saúde Pública, Universidade de São Paulo, Brasil

^3^Laboratório de Parasitologia Médica e Biologia de Vetores, Faculdade de Medicina, Universidade de Brasília, Brasil

^4^Departamento de Patologia Básica, Setor de Ciências Biológicas, Universidade Federal do Paraná, Brasil

dougalmeidarocha@gmail.com

Phlebotomine sand flies (Diptera: Phlebotominae) present wide distribution and may be found in distinct ecosystems. Worldwide there are 966 species, 273 of which are recorded from Brazil and 125 of those are from the Central-West Region (CWR). However, only 20 species have been incriminated or are suspected in *Leishmania* transmission in the country; seven of which occur in the CWR. The proposed taxonomic classification of American sand flies based on phylogenetic analysis separates the tribe Phlebotomini into four subtribes: Brumptomyiina, Sergentomyiina, Lutzomyiina, and Psychodopygina, elevating subgenera to genera. With the use of molecular methods, mainly in *Leishmania* detection, thoracic structures and a major portion of the abdomen are removed for DNA extraction, thus important morphological characters used in species identification are lost. To compensate for this, we present here an identification key to females of sand flies using only characteres of the head and spermathecae, using Galati’s proposed taxonomic classification. A total of 125 species included in 16 genera recorded in the CWR were grouped by subtribe and later separated by species, without the relationship between them. Based on the subtribes, all morphological characters were included in a data base for each species. Twenty photos of diagnostic structures of some species were taken and included. The sand fly fauna of the CWR was chosen because it had been reviewed and used recently, first, in four dichotomies to distinguish between subtribes of Phlebotomini resulting in four entrances to Brumptomyiina, 10 for Sergentomyiina, 37 for Psychodopygina, and 40 for Lutzomyiina, total in 94 dichotomies. Thoracic characters such as coloration, setal distribution and number, legs and wing venation were excluded, in consequence of which some species of the genera *Evandromyia* (2 spp.), *Psathyromyia* (4 spp.), *Pintomyia* (3 spp.) *Psychodopygus* (3 spp.) and *Lutzomyia* (5 spp.) may be indistinguishable. Thus giving other characters specific to genera, like spermatheca form and sclerotization, posterior spurs of the ascoids, antennal papilla higher priority in separating and disingquishing genera (e.g. *Psychodopygus*, *Psathyromyia* and *Lutzomyia*) and including the majority of species to minimize entrances in the key. This proposal may be used by only researchers and secretary health of the CWR of Brazil. Sixteen genera were tested and the key was validated.

**First record of *Psychodopygus francoisleponti* Zapata, Depaquit & Léon 2012 (Diptera: Psychodidae) in Acre State, Brazil**

Andreia Fernandes Brilhante^1^, Márcia Moreira de Ávila^2^, Rodrigo Espíndola Godoy^1^, Jailson Ferreira de Souza^3^, Cristiane de Oliveira Cardoso^4^, Eunice Aparecida Bianchi Galati^1^

^1^Faculdade de Saúde Pública da Universidade de São Paulo, São Paulo, Brazil

^2^Instituto Federal do Acre, Rio Branco, Brazil

^3^Gerência de Endemias, Prefeitura Municipal de Xapuri, Xapuri, Acre, Brazil

^4^Centro de Ciências da Saúde e do Desporto, Universidade Federal do Acre, Rio Branco, Brazil

brilhanteaf@usp.br

The Guyanensis series Barretto, 1962 of the *Psychodopygus* genus (Psychodidae, Phlebotominae) comprises seven species, *Psychodopygus guyanensis* (Floch and Abonnenc, 1941), *Ps. geniculatus* (Mangabeira, 1941), *Ps. lainsoni* (Fraiha and Ward, 1974), *Ps. corossoniensis* (Le Pont and Pajot, 1978), *Ps. dorlinsis* (Le Pont and Desjeux, 1982), *Ps. luisleoni* (León et al., 2009) and *Ps. francoisleponti* Zapata, Depaquit and Léon 2012. Both sexes of this group are characterized by the absence of anepimeral setae; in the males the gonostyle has one large terminal spine and three atrophied subapical spines and in the females the individual spermathecal ducts are longer than the spermathecae. The males may be distinguished by morphological differences in their parameres and the females are morphologically very similar, except *Ps. lainsoni*, which has a pale mesonotum. *Ps. francoisleponti*, described from Ecuador, is reported here for the first time in Acre State, Brazil. In entomological surveys carried out in Amazonian forested areas of Xapuri municipality, Acre state, Brazil, using CDC light traps, from August 2013 to July 2015, two male specimens of *Ps. francoisleponti* were collected in February 2014. The specimens of Acre present morphometry of FI, Labrum-epipharynx, gonocoxite, gonostyle, paramere, parameral sheath and epandrial lobe similar to that of the type-specimens, however, their aedeagal ducts (635 μm; 639 μm) are shorter than those of the type-specimens (715–815 μm). Previously, *Ps. francoisleponti* was registered only in Ecuador. This new record for the state of Acre, significantly extends the geographical distribution of this species.

**Morphological and morphometric characters to distinguish females of three sympatric species of the genus *Trichophoromyia* (Diptera: Psychodidae: Phlebotominae) in a Brazilian Amazonian area**

Andreia Fernandes Brilhante, Priscila Bassan Sábio, Eunice Aparecida Bianchi Galati

Faculdade de Saúde Pública da Universidade de São Paulo, São Paulo, Brazil

brilhanteaf@usp.br

Members of the genus *Trichophoromyia* Barretto, 1962, comprised of 40 species, are abundant through Amazonian region. Both sexes of this genus are characterized by the absence of ventro-cervical sensillae, and presence of Newstead’s sensilla in palp II; the males present large terminalia, equal to or greater than the length of the thorax, the gonostyle with four strong, short spines, with the apical commonly shorter than the length of the gonostyle; the females present maxillary laciniae with two rows of external teeth and spermathecae with more than 20 rings, with the terminal knob being sessile or detached from the spermatheca. This latter characteristic is present in most species of the genus and the females are indistinguishable. In a study undertaken to investigate the sand fly fauna in several sites in an endemic focus of cutaneous leishmaniasis in a Brazilian Amazonian area (Xapuri municipality, Acre state), three species *Trichophoromyia octavioi* (Vargas, 1949), *Th. auraensis* (Mangabeira, 1942) and *Th. ruifreitasi* Oliveira et al., 2015 were collected, but their females were not distinguishable based on information in the literature. At some sites, captured males of *Th. octavioi* were predominantly collected together with a few specimens of *Th. ruifreitasi* and it was noted the *Th. octavioi* labrum-epipharynx (LE) is longer than that of *Th. ruifreitasi*. For females, this separation into two classes of LE values was observed also. At several other sites where the three species were collected, a gradient of LE values without a clear separation was observed. Subsequently, specimens of *Th. auraensis* from other areas (deposited in the laboratory collection), where the other two species are absent, were evaluated. With this strategy it was possible to analyse the LE length as a diagnostic character besides others for the females. Ten specimens of both sexes of each species were cleared and mounted between slide and coverslip and measurements (in μm) of head, thorax and genitalia characters of each were evaluated by analysis of variance. Multiple comparisons of the morphometric characters were performed using the Gabriel test (*F* test, *p* < 0.05). The males can be distinguished by the number of bristles in the clusters situated at the middle region of the gonocoxite. *Trichophorpmyia octavioi* has *ca*. 14 long and thick bristles; *Th. auraensis* has 26 long bristles, thinner than those of *Th. octavioi*. *Trichophoromyia ruifreitasi* bears over 35 long bristles similar to those of *Th. auraensi*, and ventrally to this group of bristles, another set of *ca*.12 spiniform setae. Among the morphometric characters of the males, only the gonostyle length distinguished the three species: *Th. octavioi*, *Th. auraensis* and *Th. ruifreitasi* measure 216 μm, 195 μm and 185 μm, respectively (*F* = 46; *p* < 0.05). For females, only the length of the LE distinguish the three species: *Th. octavioi*, *Th. auraensis* and *Th ruifreitasi* with values of 376 μm, 331 μm and 302 μm, respectively (*F* = 98; *p* < 0.05). Other male characters such as ratio between the length of head/LE, LE/F1, head/F1; gonocoxite/gonostyle distinguish only between two of the species. For females the length of head, clypeus and palpus III also may be used to distinguish between two species. Although these results suggest the possibility to distinguish the three species, an analysis with more robust samples may reveal other diagnostic characters.

**Metaphase karyotyping organization of *Lutzomyia cruzi* – preliminary result**

Mirella Ferreira da Cunha Santos^1^, Natália Camargo Braga^2^, Douglas Araújo^3^, Lucas Osti de Freitas^2^, Wagner Fernandes^4^, Elisa Teruya Oshiro^4^, Alessandra Gutierrez de Oliveira^4^

^1^State University of Mato Grosso do Sul (UEMS), School of Medicine, Campo Grande, Brazil

^2^Federal University of Mato Grosso do Sul (UFMS), Center of Biological and Health Sciences, Campo Grande, Brazil

^3^Federal University of Mato Grosso do Sul (UFMS), Biology laboratory, Campo Grande, Brazil

^4^Federal University of Mato Grosso do Sul (UFMS), Parasitology laboratory, Campo Grande, Brazil

mirella.santos@uems.br

Visceral Leishmaniasis (VL) is a disease caused by a flagellate protozoan, *Leishmania infantum* (family Trypanosomatidae). In the Americas, the main vector of this disease is the female sand fly of the species *Lutzomyia longipalpis.* A closely related species, *Lutzomyia cruzi* is implicated as the vector of *L. infantum* in some regions of Brazil, including the state of Mato Grosso do Sul. Because the females of these two species are morphologically indistinguishable, the aim of this study was to describe the organization of the metaphase karyotyping of *Lu. cruzi*. The cerebral ganglia of 44 fourth-instar larvae were dissected, fixed, mounted on slides, stained by conventional Giemsa and photographed for karyotype and chromosome analysis. In the slide images it was possible to count the chromosomes of *Lu. cruzi*, *n* = 10, whereas *Lu. longipalpis* is *n* = 8. The establishment of proper cytologic preparations to confirm this preliminary finding is needed, since this taxonomic tool could help entomologists differentiate and identify females of both species. Moreover, due to frequent VL epidemics in the state of Mato Grosso do Sul, the study of these vectors may help public authorities promote more effective preventive measures.

**Phylogeography and genetic variability of populations of *Lutzomyia longipalpis* (Diptera: Psychodidae) inferred from ND4 gene**

Angélica Pech-May^1,2^, Janine Ramsey^2^, Domingo Liotta^3^, Magali Giuliani^1^, Pablo Berrozpe^1^, María Gabriela Quintana^1,4^, Oscar Daniel Salomón^1^

^1^Instituto Nacional de Medicina Tropical, Ministerio de Salud de la Nación, Consejo Nacional de Investigaciones Científicas y Técnicas (CONICET), Puerto Iguazú, Misiones, Argentina

^2^Instituto Nacional de Salud Pública / Centro Regional de Investigación en Salud Pública, Tapachula, Chiapas, México

^3^Universidad Nacional de Misiones, Posadas, Argentina

^4^Universidad Nacional de Tucumán-CONICET, Instituto Superior de Entomología, San Miguel de Tucumán, Argentina

apechmay@gmail.com

*Lutzomyia longipalpis* is the most important vector in the transmission of *Leishmania infantum* in the Americas; it is widely distributed from southern Mexico to northern Argentina. This species is actually a complex of at least four sister species. In Argentina, it is distributed in the provinces of Misiones, Formosa, Corrientes, Entre Ríos and Salta. We report here the preliminary analysis of phylogeography and genetic variability of Argentinian populations of *Lutzomyia longipalpis* inferred from the ND4 gene. For the phylogeographic analysis, we used Genbank sequences of the mitochondrial ND4 gene from Brazil, Colombia, Venezuela, Honduras, Costa Rica, and Guatemala, in addition to our 73 sequences from Argentina: Clorinda (Clo), Corrientes (Corr), Puerto Iguazú (Ig), San Ignacio (SI), Santo Tome (ST) and Tartagal (Tar). Phylogenetic analysis indicates that *Lu. longipalpis* populations from Argentina divide into two clades, (1) Corr, ST, Clo, Ig, Tar and SI; (2) Tar, ST and SI. These two clades were separated from Colombian, Venezuelan, Honduran, Costa Rican, and Guatemalan populations. The second Argentinian clade was grouped with populations from Jacobina and Lapinha in Brazil. In terms of Inter-population genetic diversity, 68 polymorphic sites were determined and 35 haplotypes were identified, with a range of 4–14 haplotypes per population. Haplotype diversity was high *Hd* = 0.858 ± 0.039, while nucleotide diversity and nucleotide polymorphism were low (*π* = 0.014 ± 0.001 and *θ* = 0.022 ± 0.002, respectively). Neutrality tests were not significant for population expansion. ST and Tar had the highest genetic diversity among the six populations, while Ig and Corr had the lowest. Population expansion was found in Corr and Ig, while ST and Tar had population subdivisions. The population structure of *Lu. longipalpis* with the ND4 fragment showed high genetic differentiation among Argentina populations (*Fst* = 0.452, *p* < 0.0001). High genetic differentiation was observed between SI and Corr populations (*Fst* = 0.86, *p* < 0.0001), with a genetic distance of 0.017 ± 0.004. According to these preliminary results, divergence patterns may be associated with climatic and physiographic discontinuities. In order to understand better dispersal routes and clades involved in Argentinian populations, samples from the neighboring countries Bolivia, Paraguay, Uruguay and more samples of Brazil will be analyzed.

**It is time to use a non destructive method for DNA extraction from phlebotomine sand flies**

Julian Gratiaux^1^, Eva Krupa^1^, Thibault Valecillo^1^, Denis Augot^1^, Véronique Lehrter^1^, Jean-Charles Gantier^2^, Jean-Yves Rasplus^3^, Jérôme Depaquit^1^

^1^Université de Reims Champagne Ardenne, ANSES, SFR Cap santé, EA 4688 – USC « Transmission Vectorielle et Épidémiosurveillance de Maladies Parasitaires (VECPAR) », Reims, France

^2^Laboratoire des Identifications Fongiques et Entomologiques (LIFE), Mennecy, France

^3^INRA, UMR 1062 CBGP Montferrier-sur-Lez, France

julian_51@hotmail.fr

Before the first application of molecular biology to phlebotomine sand fly systematics in the late 80’s, all the specimens were mounted *in toto.* Depending of the goals of the studies (epidemiology, systematics, etc.), the mounting media differed. In epidemiological studies carried out in the field just to identify the specimens and determine if their guts contain *Leishmania*, a very quick mounting in Marc-André medium was sufficient, thus precliding any possible storage of the specimen without remounting. For systematics applications, the first step every time is lysis of the soft tissues in KOH in order to observe internal characters such as spermathecae, cibarium or pharynx. Then, the specimens are washed in an acid solution like Marc-André in order to clear the tissues without destroying of the characters used for the identification. Then, it is possible to mount the specimen in an aqueous medium like chloral gum or CMCP-9 low viscosity mountant, correctly exhibiting spermathecae. However, these are non-permanent media due not enable long-term storage. For long-term storage of mounted specimens, the classical protocol is to dehydrate the specimens through an ethanol series of increasing concentrations, then beech creosote, and finally mount them in Canada balsam. The use of molecular techniques for systematics requires DNA extraction from a single sand fly. On the one hand, it is necessary to obtain enough DNA to carry out the molecular studies. On the other hand, it is necessary to preserve enough parts of each specimen to identify it. The process of isolating organs (head, genitalia, wings) is difficult, time-consuming and there is always a possibility of damaging or losing some of them. We propose a non-destructive method of DNA extraction coupled with easy processing and mounting of the whole specimen. Briefly, we use Qiagen reagents. The sand fly is placed in ATL buffer and proteinase K at 56 °C overnight. Then the buffer AL and ethanol are added after a step at 70 °C. Then the whole sand fly is removed and the DNA extraction is done on a column according to a home-made protocol. The sand fly is then cleared and dehydrated in a 1.5 mL vial in successive reagents as follows: 10% KOH, distilled water, Marc-André solution including acid fuchsin, distilled water, 70% ethanol, then 95% ethanol. Once cleared and dehydrated, the specimen is mounted in Euparal for long-term storage.

## Systematics and phylogeny (oral communications)

**Keynote – Fossil contribution in the classification of Psychodidae**

Dany Azar

Nanjing Institute of Geology and Palaeontology, Chinese Academy of Sciences, Nanjing 210008, People’s Republic of China; Lebanese University, Faculty of Science II, Fanar, Natural Sciences Department, Fanar – Matn, PO Box 26110217, Lebanon

azar@mnhn.fr

The family Psychodidae comprises nematoceran flies of more than 2,700 described species. At present it is largely accepted that this family comprises six recent subfamilies, namely Horaiellinae, Bruchomyiinae, Phlebotominae, Psychodinae, Sycoracinae and Trichomyiinae. Phlebotomine sand flies (Phlebotominae) are a group of Diptera with blood-sucking adaptations. They are either considered as belonging to the family Psychodidae, or to separately distinct family Phlebotomidae forming with the Psychodidae the superfamily of Psychodoidea. Nevertheless the latter opinion is not supported by any phylogeny. The fossil record of Psychodidae is relatively rich (around 150 described species), and representatives of this group were found in Cretaceous Lebanese, Spanish, Taimyr, New Jersey, French Charente Maritime and Burmese amber, and in Cenozoic Baltic, Saxonian, Ukrainian, Oise, Mexican, Saint Domingo and Indian ambers. The earliest definite phlebotomine record is from the Lower Cretaceous Lebanese amber, but this group could be older as Phlebotominae are well diversified in this material. Moreover several fossil psychodids that could be assigned to Psychodinae (especially the Cretaceous ones) have developed mouthparts and phlebotomine-type genitalia to the point that their exact subfamilial assignment is rather difficult and can lead to confusion. Recently several fossil flies that could be assigned to Tanyderidae if only the wings are taken into account, could be attributed to psychodids as well when considering the remaining parts of the body especially head, mouthparts and genitalia structures. One can observe all states of gradual change of the characters from Tanyderidae to Psychodidae when taking into consideration the fossils. It is noteworthy that Hennig (1973) placed Tanyderids and Psychodidae as a sister groups within the Psychodomorpha, while Wood & Borkent (1989) and Oosterbroek & Courtney (1995) placed Tanyderidae within the Ptychopteromorpha. Support for a relationship between the Tanyderidae and Psychodidae is not completely unexpected – wing venation characters, including five radial veins reaching the wing margin, even have been used to unite these taxa in the past considered that fossil Tanyderidae and Psychodidae are very similar, so much so that confusion of fossil taxa has occurred. Recent molecular analyses support a tight relationship between these two families. Based on the fossil record and molecular data, it appears likely that sooner or later the Tanyderidae and Psychodidae will be united in a single family.

**Geometric and linear morphometry as a tool for discriminating cryptic female specimens of *Psychodopygus* genus Chagasi series**

Rodrigo Espíndola Godoy^1,2^, Elizabeth Ferreira Rangel^2^, Eunice Aparecida Bianchi Galati^1^

^1^Departamento de Epidemiologia, Faculdade de Saúde Pública, Universidade de São Paulo, Av. Doutor Arnaldo, 715, Pinheiros, São Paulo, SP – 01255-00, Brasil

^2^Laboratório Interdisciplinar em Vigilância Entomológica em Diptera e Hemíptera, Instituto Oswaldo Cruz, Fundação Oswaldo Cruz, Av. Brasil, 4365, Manguinhos, Rio de Janeiro 21040-360, Brazil

rodrigoeg@usp.br

*Psychodopygus* is a genus of great medical-veterinary importance as some species have been incriminated as vectors of American cutaneous leishmaniasis agents. Among these species, *Psychodopygus complexus* and *Ps. wellcomei* of the Chagasi series are vectors of *Leishmania* (*Viannia*) *braziliensis* in Northern Brazil. However, the great morphological similarity between Chagasi series females has been a problem for their species identification. Thus our study aimed to use geometric and linear morphometric analyses to distinguish the species of the Chagasi series based on both sexes. Five species from different Brazilian states: *Psychodopygus chagasi* (Amazonas and Rondônia), *Ps. complexus* (Tocantins and Pará), *Ps. squamiventris maripaensis* (Amapá), *Ps. squamiventris squamiventris* (Roraima) and *Ps. wellcomei* (Pará) were investigated. For the geometric morphometry, thirteen landmarks on the wings of 239 females and 108 males were evaluated through the principal components, canonical variables and centroid size analyses. The linear morphometry was evaluated by 58 characters of 419 specimens (155 males and 264 females). The differences in the characters’ means were assessed using ANOVA with Tukey’s post-test (parametric data) and Kruskal-Wallis with Dunn’s test (non-parametric). We also performed canonical variables and discriminant function analyses to cluster the female specimens through linear morphometry data using 16 characters of 182 specimens. The results of geometric morphometry analyses showed: the centroid size was significantly different (*p* < 0.05) when comparing all species except between *Ps. complexus* and *Ps. maripaensis* (both males and females). The discriminant analysis showed correct classification for all pairs of species except in female *Ps. chagasi* and *Ps. wellcomei* (89.6%). In males, 100% correct classification was obtained between *Ps. mariapensis* and *Ps. complexus*, *Ps. wellcomei* and *Ps. chagasi*, and also between *Ps. complexus* and *Ps. wellcomei*. For linear morphometry the characters that differ significantly between the five species were: in males, labrum length, gonocoxite width, aedeagal duct length, and in females, head and clypeus length. The common spermathecal duct and sternite 9 width also differentiated all the species (*Ps. wellcomei* was not evaluated for these characters). Discriminant function of linear data showed that all species had fully correct classification, except for *Ps. maripaensis* that had 95.5%. As for the cross-validation test, only *Ps. complexus* (98.8%) and *Ps. maripaensis* (90.9%) did not show completely correct classification. In conclusion, the discriminant analysis using both geometric and linear morphometric data can be used as a tool to help in the correct species identification of the Chagasi series, including their females.

Financial support: Grant 2015/02282-9, São Paulo Research Foundation (FAPESP); Instituto Oswaldo Cruz/FIOCRUZ.

**Lutzodex** ^**TM**^ **– a digital key for sand flies (Diptera: Phlebotominae) using Android App**

Douglas de Almeida Rocha^1^, Maxwell Ramos de Almeida^2^, Andrey José de Andrade^3,4^

^1^Núcleo de Medicina Tropical, Universidade de Brasília, Brasil

^2^Real Comércio e Indústria de Alumínio Ltda, Brasil

^3^Laboratório de Parasitologia Médica e Biologia de Vetores, Faculdade de Medicina, Universidade de Brasília, Brasil

^4^Departamento de Patologia Básica, Setor de Ciências Biológicas, Universidade Federal do Paraná, Brasil

dougalmeidarocha@gmail.com

Phlebotomine sand flies are small insects included in the subfamily Phlebotominae in which nowadays there are about 966 species described in worldwide. Of these, 515 have been recorded in the Neotropical region, of which 273 (53%) occur in Brazil. The Central-West region (CWR) of Brazil comprises the states of Mato Grosso, Mato Grosso do Sul, Goiás, and the Federal District, and the combined sand fly fauna of the region includes 125 species. In the Americas, sand fly taxonomy is based on the classifications and keys proposed by Young & Duncan (1994) and Galati (2003). Recently, the Brazilian Leishmaniasis Control Program of the Ministry of Health adopted the latter work, which is reviewed annually, in the routine of secretaries of health. Some identification keys, using what is called cybertaxonomy, have been proposed due to advantages they afford, especially when working with large taxa such as sand flies, thus reducing the laborious identification procedures. The data’s choice is easy and the identification may be directed to a specific location, and the generated keys may be reviewed if errors are found; new taxa and/or characters may also be added. The present study applied the development of an Android App to further phlebotomine sand fly identification. Then, an identification key for male and female sand flies recorded in the CWR was made using the above-mentioned classifications and assaying slides recorded in the region. Based on the key, a database was prepared in tabular format using the Excel program. The App, named Lutzodex^TM^, received information included in the database with morphological characters. This tool does not use conventional dichotomies, since up to seven options of response to a question may be found. In practically all the responses, the images of structures can be observed for easy identification and at any time during the process one can check the possible species, ie, species that have the same synapomorphies. Also, it is possible to verify the geographic distribution in Brazil and if the species is incriminated in *Leishmania* transmission based on the literature. The question answered can be analysed at any time. The answers may be deleted and the correct identification follows based on the last question answered. Every answer of the remaining number of species is presented. Digital keys decrease the number of questions, facilitating the identification of species. Lutzodex^TM^ is being registered by the National Industrial Property Institute and will be available to researchers, students and institutions, free of charge, and will be available in the Google Play Store.

***Phlebotomus* (*Paraphlebotomus*) *chabaudi* Croset, Abonnenc & Rioux, 1970 and *Phlebotomus riouxi* Depaquit, Killick-Kendrick & Léger, 1998: synonyms or closely related species?**

Véronique Lehrter, Jérôme Depaquit

EA 4688 – USC Anses « VECPAR », SFR Cap Santé, UFR de Pharmacie, Université de Reims Champagne-Ardenne, 51 rue Cognacq-Jay, 51096 Reims, France

veronique.lehrter@univ-reims.fr

*Phlebotomus riouxi* Depaquit, Killick-Kendrick & Léger, 1998, was described as a species closely related to *Phlebotomus chabaudi* Croset, Abonnenc & Rioux, 1970. They differ mainly by the size and number of setae on the basal lobe of the coxite. Moreover, molecular studies carried out on several populations in Algeria and Tunisia based on mitochondrial genes cytochrome *b* (cytb) and cytochrome oxidase I (COI) supported the typological validity of these two species. However, specimens recently studied by Tabbabi et al. (Med. Vet. Entomol. 2014; 28 Suppl. 1:51–59), were morphologically identified as *Ph. riouxi*, *Ph. chabaudi*, and intermediate specimens. Their cytb and nuclear gene elongation factor-1alpha (EF-1*α*) sequences showed that they belong to the same clade and the authors suggested that *Ph. riouxi* could be synonymized with *Ph. chabaudi.* In order to know if *Ph. riouxi* really is a junior synonym of *Ph. chabaudi*, we carried out a molecular study using the same molecular markers as those used by Tabbabi et al. and a part of 28S rDNA more (D1 and D2 domains). Surprisingly, we highlighted many new haplotypes very different from those obtained by Tabbabi et al. We didn’t have access to “intermediate” specimens so are not yet able to make a definite conclusion. However, there is no relation between morphotypes and haplotypes. The only observable link was between the haplotypes and the geographical origin of the populations (northern or southern) without highlighting any introgression. Consequently, as we await further studies, it seems better to consider *Ph. riouxi* and *Ph. chabaudi* as valid typological species.

**Beware of *Sergentomyia* from Southeastern Asia due to untimely synonymies and a need to describe new species**

Jérôme Depaquit

Université de Reims Champagne Ardenne, ANSES, SFR Cap santé, EA 4688 – USC « Transmission Vectorielle et Épidémiosurveillance de Maladies Parasitaires (VECPAR) », Reims, France

jerome.depaquit@univ-reims.fr

Southeast Asia has long been considered free of autochthonous transmission of *Leishmania*. Consequently, there are few data related to phlebotomine sand flies from this part of the World. However, the first autochthonous South-East Asian cases of leishmaniasis recorded in Vietnam, then in Thailand in 1996, due to *Leishmania* “*siamensis*” and, surprisingly, to *L. martiniquensis* explain a growing interest in the sand fly fauna of Southeast Asia, which remains poorly documented. Only a few studies were carried in the 1960s to the 1980s and more recently in Thailand. Except for the species described from the Southeast Asia by Raynal, all other species recorded in this area were described from India by Sinton. The paleobiogeography of India differs from that of SE Asia. Moreover, some surprising synonymizations were proposed by both Lewis and Quate. *Sergentomyia hivernus* is a valid species very curiously synonymized with *Se. iyengari*. The latter species has never been found in South-Eastern Asia and surprisingly, *Se. gemmea*, proposed as a possible vector of leishmaniasis in Thailand, is a very uncommon species, possibly confused with those previously mentioned.

## Epidemiology, laboratory studies & modern tools (posters)

**The aminosugar galactosamine reduces the trypsinolytic activity of *Lutzomyia longipalpis* (Diptera: Psychodidae) and promotes *Leishmania mexicana* and *Leishmania infantum* development within the sand fly gut**

T. Lima-Silva, L.K. Castro, A. Bortolini, Marcos H. Pereira, R.N. Araújo, N.F. Gontijo, Mauricio R.V. Sant’ Anna

Laboratório de Fisiologia de Insetos Hematófagos, Departamento de Parasitologia, Instituto de Ciências Biológicas, UFMG, Belo Horizonte, MG, Brasil

mellima9@yahoo.es

Leishmaniasis is a complex of diseases caused by protozoan parasites belonging to the genus *Leishmania* and these haemoflagellates are transmitted to their vertebrate hosts by minute insects called sand flies. The etiological agent of lethal visceral leishmaniasis in Brazil (*Leishmania infantum*) is transmitted to the vertebrate hosts by the bites of *Lutzomyia longipalpis* (Diptera, Psychodidae). Sand fly males and females are plant feeders but females must obtain a blood meal from vertebrate hosts in order to produce eggs. *Leishmania* develop within the sand fly gut and during blood digestion, ingested parasites must survive the onslaught of sand fly digestive enzymes. Trypsin is the key enzyme involved in blood digestion in arthropod vectors. During their life cycle inside their sand fly vector, *Leishmania* are most susceptible to trypsin attack during their transition from the amastigote form present in newly digested macrophages to procyclic promastigotes, which are responsible for commencing parasite replication inside the insect vector. This study aimed to manipulate the digestive physiology of *Lu. longipalpis* to increase their susceptibility and favour the development of *L. mexicana* and *L. infantum* inside the sand fly gut. Galactosamine (an amino sugar) was capable of reducing *Lu. longipalpis* tripsinolytic activity in a dose-dependent manner. This activity was specific to galactosamine as other similar molecules such as galactose, glicosamine and N-acetylgalactosamine were not able to reduce the sand fly tripsinolytic activity in the same way observed for galactosamine. An excess of amino acids supplemented with the bloodmeal and 15 mM galactosamine was able to partially abrogate the reduction of the tripsinolytic activity caused by galactosamine, suggesting that this phenomenon might be related to an impairment of amino acid detection by sand fly enterocytes when galactosamine is mixed with blood during an artificial bloodmeal. In mosquitoes, TOR signaling is the upstream activator of early trypsin synthesis in the midgut and amino acids function as stimulus for TOR signaling (see: TOR signaling is required for amino acid stimulation of early trypsin protein synthesis in the midgut of *Aedes aegypti* mosquitoes by Brandon et al., for details). Galactosamine reduces the trypsinolytic activity in the gut of *Aedes aegypti* and other nematoceran flies. In an attempt to increase *Lu. longipalpis* midgut infection by two *Leishmania* species (*L. mexicana* strain M379 and *L. infantum* strain BH401), we infected 7-day-old sand flies through chick skin membranes on a Hemotek artificial feeding device. Two groups of *Lu. longipalpis* ingested different amounts of galactosamine (15 and 30 mM final concentration) added to heparinized human blood and the guts of infected sand flies were dissected five and eight days after the infection. Parasite count was carried out using a haemocytometer and the parasite load from insects infected with *Leishmania* seeded in human blood supplemented with galactosamine was compared with control insects infected with *Leishmania* without galactosamine. The results showed that administration of 15 and 30 mM galactosamine was sufficient to increase the number of promastigote forms of *L. mexicana* and *L. infantum* in galactosamine-treated insects in comparison to their controls. The reduction of trypsin production within *Lu. longipalpis* gut and the delay in blood digestion generated by galactosamine ingestion benefited *Leishmania* growth within the sand fly gut. The inhibition of the trypsinolytic activity is part of a protocol that has been developed in our lab to generate heavily infected sand flies to be used as an ideal challenge in vaccine trials against *L. infantum*.

**Evaluation of different diets for feeding larvae of *Nyssomyia neivai* (Diptera: Psychodidae: Phlebotominae)**

Antonio Carlos Ferrari Júnior^1^, Kleiton Maciel dos Santos^1^, Magda Freitas Fernandes^1^, Wedson Desidério Fernandes^1^, Herintha Coeto Neitzke-Abreu^2^, Maria Elizabeth Moraes Cavalheiros Dorval^3^, Alessandra Gutierrez de Oliveira^3^, Eunice Aparecida Bianchi Galati^4^

^1^Faculdade de Ciências Biológicas e Ambientais, Universidade Federal da Grande Dourados (UFGD), Brasil

^2^Faculdade de Ciências da Saúde, Universidade Federal da Grande Dourados (UFGD), Brasil

^3^Centro de Ciências Biológicas e da Saúde, Universidade Federal de Mato Grosso do Sul (UFMS), Brasil

^4^Faculdade de Saúde Pública, Universidade de São Paulo (USP), Brasil

magdamattosfer@gmail.com

Studies of vector competence of sand fly species for *Leishmania* spp. require sufficient numbers of adult live females for experimental infection. In this study we aimed to establish a laboratory colony of *Nyssomyia neivai* using different larval diets to assess which diet produces the most synchronous emergence of adults in the greatest numbers for vector competence studies. Collections of wild sand flies were conducted with white Shannon traps in a forest fragment in Rio Brilhante municipality, Mato Grosso do Sul, Brazil, in September 2014. To obtain F1 adults, wild-caught males and females were kept together in breeding cages (metal frame and voile fabric), packed in polystyrene boxes coated internally with stone plaster cream and supplied with chilled towels for humidity. Apple slices were offered as a source of carbohydrate to enhance survival of the flies. For blood feeding, anesthetized hamsters (*Mesocricetus auratus*), were exposed for two hours inside the mating cage. The engorged females were placed individually in oviposition vials until they laid eggs. Then the eggs were transferred to culture plates for rearing the immature forms. Cohorts of larvae were fed on five different diets as follows: Diet 1 = vegetable soil + quail faeces; Diet 2 = yeast − *Saccharomyces cerevisiae* + B vitamins; Diet 3 = flake food for ornamental fish; Diet 4 = combination of diets 1 and 3; Diet 5 = combination of diet 1 and 2. The diets were prepared in equal parts, triturated and sieved. Each diet was tested in 10 replicates, making a total of 50 tests. The mean number of eggs per replicate was 70.3 for Diet 1; 70.5 for Diet 2; 70.6 for Diet 3; 70.2 for Diet 4 and 70.1 eggs for D 5. The gonotrophic cycle *Ny. neivai* was four days. A total of 173 adults emerged with an overall average generation time of 51 days. Time to adult emergence on Diet 1 was 25 days with 64 adults emerging, (35 males and 29 females); Diet 2 produced only one male; Diet 3 produced 52 adults (18 males, 25 females and nine not identified as to sex); Diet 4 produced 46 specimens (14 males and 32 females); and Diet 5 produced 10 adults (one male and nine females). Diet 4, although in 3rd place in the total of adults that emerged, produced the largest number of F1 females compared to other diets. There was a significant difference between Diet 1 and Diet 3, 4 and 5 (*p* < 0.05 Tukey Test). Larvae fed on Diet 2 developed to 4th instars faster than those fed on the other diets, however, all but one larva died before pupation and only one adult emerged. Thus, we concluded that Diet 1 (vegetable soil + quail faeces) was the best for the adult emergence of *Ny. neivai*, producing sufficient F1 females in 25 days for use in *Leishmania* transmission experiments.

Financial support: This work was supported by Fundect, and CAPES, the Brazilian Government for the qualification of human resources.

**Is there oviposition pheromone in *Nyssomyia neivai* (Diptera: Psychodidae)?**

Thais Marchi Goulart^1^, Camila Feitosa de Castro^2^, Wanderson Henrique Cruz Oliveira^2^, Flávia Benini da Rocha Silva^2^, Vicente Estevam Machado^2^, Dennys Ghenry Samillan Ortiz^1^, Christiann Davis Tosta^3^, Mara Cristina Pinto^2^

^1^Departamento de Biologia Animal, Instituto de Biologia, UNICAMP/Brasil

^2^Departamento de Ciências Biológicas, Faculdade de Ciências Farmacêuticas, Câmpus Araraquara, UNESP/Brasil

^3^Instituto Federal de São Paulo – IFSP, campus Matão/Brasil

marap@fcfar.unesp.br

Oviposition pheromone has already been described in *Lutzomyia longipalpis*. However, except for a biological evidence in *Lu. renei* there are no study related to this issue to other Latin America sand fly species. Oviposition pheromone increases the number of eggs laid by females. This fact is important to improve sand fly colonies of sand flies at laboratory conditions and also for possible baits to collect gravid females in field. The aim of this study was to search for biological evidences of an ovipositon pheromone in *Nyssomyia neivai*. Oviposition chambers used in the tests were made of 250 mL plastic pots (9 cm diameter and 5 cm height) with a thin layer of plaster of Paris on the bottom. In the first trial the area of the plaster of Paris was divided into six equal parts. A Falcon tube of 50 mL was cut off in the middle and fixed vertically over one part of the plaster of Paris. Three engorged females and three males were placed into the tube. After oviposition another new five engorged females and five males were placed into the oviposition chamber and five days later the number of eggs was recorded in each part of the pot. Experiment was replicated five times and the number of the initial eggs for each pot was respectively: 245, 246, 144, 155 and 114. In all five pots there was clearly a highest number of eggs in the place of the previous oviposition (*p* < 0.01). After this positive biological clue we decided to compare the attraction of gravid females of *Ny. neivai* to conspecific eggs and *Aedes aegypti* eggs. Almost the same described methodology was used, but at this time the plaster of Paris was divided into four quadrants. A total of 20 *A. aegypti* eggs were placed in an opposite quadrant to 50 *Ny. neivai* eggs. Three sand fly couples were introduced and the eggs were recorded after five days. This experiment was replicated five times. There was no statistical difference among the number of laid eggs into the quadrants (*p* > 0.05). Other experiments will be performed to investigate if volatiles of *A. aegypti* could have disrupted the attraction of a possible oviposition pheromone of *Ny. neivai* or if a visual and/or tactile response of females could be the real stimulus.

**Experimental infection of *Phlebotomus perniciosus* by bioluminescent *Leishmania infantum* using a murine model and artificial feeder**

Arnaud Cannet^1^, Mohammad Akhoundi^2,*^, Michel Gregory^1,*^, Pierre Marty^1,2^, Pascal Delaunay^1,2^

^1^Inserm U1065, Centre Méditerranéen de Médecine Moléculaire, Université de Nice-Sophia Antipolis, France

^2^Service de Parasitologie-Mycologie, Hôpital de l’Archet, Centre Hospitalier Universitaire de Nice, France

^*^Equal contribution in the present study

delaunay.p@chu-nice.fr

Leishmaniasis is protozoan disease caused by digenetic *Leishmania* parasites that circulate between vector sand flies harboring the extracellular-stage promastigotes and the mammalian hosts harboring the intracellular amastigotes. The goal of the present study was to screen experimental infections of *Phlebotomus perniciosus* with bioluminescent *Leishmania infantum* using a murine model and artificial feeder. We developed a real-time PCR-based method to determine the number of *Leishmania* promastigotes ingested by individual infected flies. Of 1,840 newly emerged female sand flies, 428 fed on mice infected with bioluminescent *L. infantum.* After death, each fly was individually analyzed by RT-PCR. Only a single female was *Leishmania* positive at six days post bloodmeal. A total of 1,070 female sand flies offered blood meals on an artificial feeder containing the human blood infected with *Leishmania* parasites as follows: A blood meal containing 1.10^7^/mL LUC-promastigotes was offered to 270 females, 75 (28%) of which engorged engorged. Of the engorged flies, 44 (59%) were positive by RT-PCR analysis, with a relative average of 50,551 *Leishmania* parasites. A second group of 800 female sand flies was offered a blood meal infected with 2.10^6^/mL promastigotes, of which 57 (7%) female flies successfully fed. Of the blood-fed flies in the second group, 22 (39%) were positive with a relative average of 6,487 parasites.

**Exploring the migration of kinetoplastid parasites in sand flies; why are hypopylarian parasites backward in coming forward?**

Raquel J. Vionette-Amaral, C.T. Nogueira, M. Ginger, Rod J. Dillon

Faculty of Health and Medicine, Division of Biomedical and Life Science, Lancaster University, UK

r.vionettedoamaral@lancaster.ac.uk

The development of human pathogenic *Leishmania* in the gut of sand flies is split between suprapylarian and peripylarian states. Suprapylarians such as *L. infantum* and *L. mexicana* of subgenus *Leishmania* usually confine their developmental cycle to the midgut and foregut. *Leishmania braziliensis* of subgenus *Viannia* are peripylarian, developing first in the hindgut and migrating forward to the midgut and then foregut. In contrast, members of the subgenus *Sauroleishmania* are thought to be hypopylarian. Hence they are mainly confined to the hindgut and transmission is either by the faecal route or ingestion of the infected sand fly by the vertebrate. We are exploring the hypopylarian state using *L. tarentolae* (subgenus *Sauroleishmania*) and *Crithidia fasciculata* with the permissive sand fly *Lutzomyia longipalpis. L. tarentolae* is an intracellular parasite of reptiles that uses phlebotomine sand fly vectors. It is not clear whether infection in lizards occurs through cutaneous transmission, via the bite or faeces of the vector, or through direct ingestion of the sand fly. Once ingested by the vector, the parasites multiply as promastigotes in the gut. It is currently unknown what developmental forms of *L. tarentolae* exist in the vector. *C. fasciculata* are extracellular monogenic flagellates that naturally infect members of the Culicidae. Female *Lu. longipalpis* (Jacobina strain) were infected with the two species and observed during a period of 10 days post infection. We analysed the percentage of infection, mortality rate, morphology, and localization of the parasites in the gut. We also investigated the effect of sand fly feeding mode on *C. fasciculata* infection. For infection with *C. fasciculata*, the flies were fed with culture medium or with sucrose solution 10% v/v 0.5% bromophenol blue containing the parasites either through a chicken-skin membrane or cotton wool. The infection with *L. tarentolae* was done by feeding sand flies on sheep blood containing the parasites. Gut infections for both kinetoplastids were observed including parasite adhesion to the hindgut cuticle. The results of these infection studies will be used to explore hyopylarian vs. peripylrian and suprapylarian development in this permissive sand fly and to understand why these parasites may be backward in coming forward.

**Molecular and serological methods for evaluating blood meal sources in phlebotomines sand flies (Diptera: Psychodidae)**

Mauricio Baum^1^, Edilene Alcântara de Castro^1^, Elias Seixas Lorosa^2^, Mara Cristina Pinto^3^, Thais Marchi Goulart^3^, Walter Baura^1^, Magda Clara Vieira da Costa-Ribeiro^1^

^1^Federal University of Paraná, Biological Sciences Sector, Polytechnic Center, Curitiba, Paraná, Brazil

^2^Oswaldo Cruz Foundation, Rio de Janeiro, Brazil

^3^State University of Campinas, Institute of Biology, Department of Animal Biology, Campinas, São Paulo, Brazil

magdacostaribeiro@gmail.com

American cutaneous leishmaniasis (ACL) is a noncontagious infectious disease caused by different species of protozoa of the genus *Leishmania*, which infect skin and mucosa. In Brazil, ACL is widely distributed in all regions. It is very difficult to determine the reservoirs of this parasite in several areas in which it occurs. The blood meal source of sand flies provides valuable information about vector/host interactions and allows better understanding of *Leishmania* transmission mechanisms. The precipitin test based on Ag-Ab reactions is used largely for blood meal source identification and allows the identification of a wide variety of hosts as long as the specimens are tested with several antisera. This is an easily performed, inexpensive method with relatively good sensitivity and easily interpreted results. The identification of multiple blood meal sources through precipitin tests permits verification of whether or not a species exhibits opportunistic feeding behavior, but does not allow reservoir identification at the species level. Other limitations include the requirement to produce specific antibodies to a wide range of potential hosts, and the inability to discover unpredicted reservoirs. Recently, molecular approaches have been developed in order to identify vector blood meal sources, such as those based on the amplification and sequencing of the preponociceptin (PNOC) gene. Successful blood meal identification via molecular means depends on the amount of blood ingested and length of time it has been in the in the insect midgut expose to digestive enzymes. Our data show that it is possible to identify the blood meal source using the PNOC gene up to 24 h after a blood meal. Finally, the current method based on PNOC gene amplification revealed a promising tool for identify in the blood-meal sources of female sand flies in an endemic area of American cutaneous leishmaniasis. Investigations of the blood-meal source of sand flies have great ecological and epidemiological significance because they enable correct identification of the mammalian reservoirs and vector feeding preferences.

**Host feeding preference and molecular screening of *Leishmania* infection in wild-caught sand flies in an endemic focus Aydın, Turkey**

Mehmet Karakuş^1^, Metin Pekağırbaş^2^, Samiye Demir^3^, Hasan Eren^2^, Seray Töz^1^, Yusuf Özbel^1^

^1^Ege University, Faculty of Medicine, Department of Parasitology, Izmir, Turkey

^2^Adnan Menderes University, Faculty of Veterinary, Department of Parasitology, Aydın, Turkey

^3^Ege University, Faculty of Science, Department of Biology, Izmir, Turkey

metinpekagirbas@gmail.com

Leishmaniasis is an arthropod-borne disease, which affects more than two million people worldwide annually. There are three main clinical forms of leishmaniasis (mucocutaneous, cutaneous and visceral) and both cutaneous and visceral forms exist endemically in Turkey. The aims of the study were to detect natural *Leishmania* infection and feeding preferences of probable vector species by DNA-based techniques for a better understanding of the epidemiology of the disease in this area. Entomological sampling was done between 28–31 August and 01–03 October 2015 in five villages of Aydın province, where leishmaniasis cases, both visceral and cutaneous, were previously reported. Besides species level identification of the sand fly specimens, molecular screening using kinetoplast DNA and the ITS1 region of nuclear DNA was carried out using pooled groups of sand flies to determine natural *Leishmania* infection and individual blood meal analysis was conducted in order to determine probable host species. A total of 1,059 sand fly specimens belonging to 2 genera (*Phlebotomus* and *Sergentomyia*) and 5 subgenera (*Phlebotomus*, *Paraphlebotomus*, *Larroussius*, *Adlerius* and *Transphlebotomus*) were collected in five villages. *Ph. neglectus* (39%) was the most abundant species in the study area followed by *Ph. tobbi* (18%) among all *Phlebotomus* specimens. *Leishmania* infection was detected in *Ph. neglectus* (3 pools), *Ph. tobbi* (3 pools) and *Se. dentata* (1 pool). The *Leishmania* species found in *Phlebotomus* specimens was identified as *L. infantum* by species-specific real time ITS1 PCR. A total of 33 specimens were used for blood meal analysis and mostly human (8/33) and dog (10/33) DNA was found in *Ph. neglectus* and *Ph. tobbi*. The detection of natural *Leishmania* infection in wild caught *Ph. neglectus* and a high percentage (24.2%) of that species with of human DNA in engorged specimens suggest that *Ph. neglectus* is probably an important vector in Aydın. The data obtained by this study could help public health authorities take necessary and appropriate precautions.

Financial support: Partially by The Scientific and Technological Research Council of Turkey (TÜBİTAK) Project No: 114S999.

**Anthropophilic behaviour and detection of *Leishmania* spp. in *Sergentomyia minuta* collected in the human leishmaniasis focus of Madrid, Spain**

Estela González^1^, Ana Tello^2^, Ricardo Molina^1^, Andrés Iriso^3^, Ángeles Vázquez^2^, Maribel Jiménez^1^

^1^Medical Entomology Unit, Parasitology Service, National Centre of Microbiology, Institute of Health Carlos III, Majadahonda, Madrid, Spain

^2^Zoology and Physical Anthropology Department, Faculty of Biological Science, Complutense University of Madrid, Madrid, Spain

^3^Zoonosis and Biological Risk Section, General Directorate of Public Health, Madrid Regional Health Authority, Community of Madrid, Madrid, Spain

mjimenez@isciii.es

Anthropophilic behaviour as well as detection of DNA from *Leishmania major* and *L. tarentolae*-like has recently been reported in *Sergentomyia minuta* from Mediterranean countries. As a result of such findings questions on the role of the *Sergentomyia* genus in *Leishmania* spp. transmission needs to be clarified. Since 2010 an unusual increase of human leishmaniasis cases due to *L. infantum* has taken place in urban areas of the southwest Madrid region, Spain, mainly in the town of Fuenlabrada. *Phlebotomus perniciosus* is the only proven vector in this focus however *Se. minuta* is the second most abundant species in the area with a mean average density of 106.81 specimens/m^2^. With the aim of providing more information about the potential role of *Se. minuta* in the transmission of *Leishmania* spp. in the focus, we carried out an analysis of blood-meal preferences and the detection of *Leishmania* spp. in this sand fly species*.* A total of 107 *Se. minuta* females (*n =* 84 blood-fed and *n =* 23 unfed) collected throughout the transmission seasons of 2012–2015 with both sticky (*n =* 81) and CDC light traps (*n =* 26) were studied. Traps were placed in different stations located in four municipalities of the southwestern Madrid region. Blood-meal identification was conducted in 84 blood-fed *Se. minuta* females collected with both sticky (*n =* 81) and light (*n =* 3) traps. Amplification of a fragment of 359 bp of vertebrate cytochrome-*b* gene was carried out. Afterwards, the samples were sequenced and comparison with sequences deposited in GenBank^®^ was performed by BLAST. In 16 (19%) out of 84 specimens amplification was successful and the analysis revealed that *Se. minuta* collected with sticky traps had fed mainly on gecko (*Tarentola mauritanica*, *n =* 8) and lizard (*Podarcis hispanica*, *n =* 2), followed by rabbit (*Oryctolagus cuniculus*, *n =* 1). Surprisingly, in the remaining *n =* 5 (31.3%) specimens from sticky (*n =* 3) and light traps (*n =* 2), human (*Homo sapiens*) blood was found. Detection of *Leishmania* spp*.* was carried out by amplification of a 120 bp fragment of the conserved region of kDNA. Samples were further analysed following a specific *L. infantum* PCR based on the amplification of a fragment of 702 bp of *cpb* gene. *Leishmania* spp. was detected in 10 out of 84 blood-fed *Se. minuta* using kDNA-PCR: 8 were from the group of 68 *Se. minuta* with an unidentified blood meal source and the other 2 were from the group of 16 specimens with an identified blood meal (one had fed on gecko and one on human). In addition, 1 out of 23 unfed *Se. minuta* successfully studied was positive for *Leishmania* spp*.* by kDNA-PCR. Further analysis by PCR of the *cpb* gene excluded *L. infantum* infection in all the samples. Moreover, amplification of the internal transcribed spacer regions 1 (ITS1) and ITS1-RFLP was performed in kDNA-PCR positive samples (*n =* 11). Amplification of ITS1 region and further sequencing was successfully achieved in four DNA samples named CM145, CM146, CM148, and CDC290. Sequences obtained were analyzed and homologies with the available sequences data in GenBank^®^ were carried out by BLAST. The four sequences presented between 93 and 99% identity with sequences of *Leishmania* spp. (LC028233.1 and LC028235.1) from Portugal, *Leishmania* spp. (LC031456.1) from Spain and *L. tarentolae*-like (LC086296.1). With the present work we describe for the first time the anthropophilic behavior of a *L. tarentolae*-like positive *Se. minuta* collected in the focus of human leishmaniasis in Madrid and conclude that there is no evidence that *Se. minuta* is the vector of *L. infantum* in the Madrid focus.

**Molecular detection of *Leishmania tropica* parasites kDNA from naturally infected sand flies in a new foothill endemic area, southeast Iran**

M.D. Moemenbellah-Fard^1^, K. Azizi^1^, M.R. Fakoorziba^1^, T. Dabaghmanesh^2^, M. Ahmadyousefi-Sarhadi^2^

^1^Research Centre for Health Sciences, Department of Medical Entomology and Vector Control, School of Health, Shiraz University of Medical Sciences, Shiraz, Iran

^2^Department of Medical Entomology, School of Health, Shiraz University of Medical Sciences, Shiraz, Iran

dabaghmanesh@gmail.com

Human dermal leishmaniasis caused by *Leishmania tropica* is a serious public health concern in Iran and the adjacent countries of the Middle East and Central Asia. It is usually known as the urban oriental sore. The sand fly *Phlebotomus sergenti*, which transmits the protozoan parasites to man, is the principal vector. Little is known about the sand fly fauna or their distribution in southeast Iran and no research has been conducted on natural infection of sand flies in Jiroft, Kerman province, Iran. The main aim of this study was to determine the sand fly faunal composition, its’ frequency distribution and the occurrence of natural infection with *Leishmania* parasites to incriminate the likely vectors of dermal leishmaniasis in Jiroft. Sand flies were caught with sticky traps during a six month period in 2013. They were identified to species level using taxonomic keys. They were then subjected to nested polymerase chain reaction method and the results were analyzed to confirm *Leishmania* infection. A total of 3,751 sand flies, belonging to 21 species in two genera (8 spp. in the *Phlebotomus* genus, and 13 spp. in the *Sergentomyia* genus) were identified, of which males were 65.5% and 63.8% were exophilic. The two species most frequently captured were *Ph. papatasi* (39.4%) and *Ph. sergenti* (17.1%). The latter was confirmed as being naturally infected with *L. tropica* (3.33%). It was thus concluded that *Ph. sergenti* which was found by PCR to be infected with *L. tropica* is the principal vector of human dermal leishmaniasis in this endemic focus.

**Epidemiology of cutaneous leishmaniasis in the municipality of Brasiléia, Acre State: Study on the sandy fly fauna**

Thais De Araujo-Pereira^1^, Daniela De Pita-Pereira^1^, Mariana Boité^2^, Daniella Alves Martins^1^, Taina A.N. Da Costa-Rego^1^, Israel De Souza Pinto^3^, Regina Barbosa Moreira^4^, Andressa A. Fuzari^5^, José Dilermano Andrade-Filho^3^, Marcia Oliveira^4^, Reginaldo Brazil^5^, Constança Britto^1^

^1^Laboratório de Biologia Molecular e Doenças Endêmicas/IOC, Brasil

^2^Laboratório de Pesquisa em Leishmanioses/IOC, Brasil

^3^Centro de Referência Nacional e Internacional para Flebotomíneos/Centro de Pesquisa René Rachou/Fiocruz, Brasil

^4^Laboratório Interdisciplinar de Doenças Medicas/IOC, Brasil

^5^Laboratório de Doenças Parasitárias/IOC, Brasil

pereirathata@gmail.com

Despite an increase in cases in recent years and 1,009 notifications of cutaneous leishmaniasis in 2013, there are few studies concerning the situation with cutaneous leishmaniasis (CL) in Acre State, Brazil. In this study we investigated the heterogeneity of sand flies captured in the municipality of Brasiléia to bring insights into the epidemiology of CL in the Acre State. Following the species identification we evaluated *Leishmania* infection rates, the blood meal sources and correlated the parasite species found in the sand flies with the parasite species detected in the lesions of patients from the same region. Sand fly collections were made from September 2013, using light traps and manual capture in Shannon trap and taxonomically identified. In parallel, biopsy imprints were obtained from patients with CL lesions living in Brasiléia. Parasite detection in insects was performed individually, in non-blood-fed females, using a multiplex PCR with primers for *Leishmania* genus (kDNA) and sand fly gene (*cacophony*). For the accurate identification of *Leishmania* species, PCR targeting the parasite *hsp70* gene followed by sequencing was done. A total of 5,969 sand flies (2,170 males; 3,705 non-blood-fed females (889 used for molecular diagnosis); 94 blood-fed females (for the blood meal research) were collected at seven trapping sites on four separate occasions. So far, we have identified 14 sand fly genera and 63 species by morphological identification. To determine natural infection rates, 489 females were processed individually and *Leishmania* DNA was identified in 22 specimens (4.5%). Clinical samples were obtained from 23 CL patients. From these, 8 (34.8%) were infected by *L.* (*V.*) *braziliensis*, one (4.3%) corresponded to *L.* (*V.*) *guyanensis*. Infections in 10 patients (43.5%) were not identified to species level, and 4 (17.4%) were found not infected. With the conclusion of this study, we expected to provide new elements for better understanding the CL transmission cycle in the study area.

Financial support: CNPq, FAPERJ, CAPES, Fiocruz.

**Seasonal dynamics, evolution of *Leishmania infantum* infection rates, and host-feeding preferences of *Phlebotomus perniciosus* in the focus of human leishmaniasis in the Madrid region, Spain (2012–2014)**

Ricardo Molina, Estela González, Sonia Hernández, Inés Martín-Martín, Maribel Jiménez

Medical Entomology Unit, Parasitology Service, National Centre of Microbiology, Institute of Health Carlos III, Majadahonda, Madrid, Spain

rmolina@isciii.es

Since 2010 the number of cases of human leishmaniasis in south western Madrid, Spain has increased while the prevalence of canine leishmaniasis remains unchanged or lower compared nearby areas. *Phlebotomus perniciosus* is the only vector identified in the area in high numbers. The objective of this work was to carry out a detailed and intensive entomological survey to obtain information on *Ph. perniciosus* seasonal trends; sand fly densities, monthly *Leishmania infantum* infection rates and blood-meal preferences in this exceptional human focus of leishmaniasis that is affecting several urban areas and most particularly, Fuenlabrada town. The studies were carried out during the transmission seasons of 2012, 2013 and 2014. The entomological surveys were performed monthly from May to October at 4 sites located beside the urban area of the focus. Sticky traps (20 × 20 cm) (*n =* 20) and CDC light traps (*n =* 2) were placed at each of the 4 sites station each month during two consecutive nights (CDC traps were replaced every day). Temperature and relative humidity (RH) data were registered every 10 min using data loggers hanging on each light trap. For phenology studies data recorded from dusk to dawn were used. In the seasonal study 45,127 *Ph. perniciosus* (75.34%), the predominant sand fly species in the area, were collected. *Ph. perniciosus* average density obtained with sticky traps in the three surveys was 193.6 specimens/m^2^. The density showed two annual peaks one in June and one in August 2012, while in 2013 a small peak in July and a bigger one in September were observed. Only one annual peak was observed in September 2014. Average relative abundance of *Ph. perniciosus* obtained from light trap collections was 94.24%. Statistical analysis showed that there was a negative correlation between numbers of sand flies captured (by either light or sticky traps) and RH (mean, maximum and minimum). Sand fly captures were positively correlated with temperature (mean, maximum and minimum) in both light and sticky traps. *Ph. perniciosus* females (*n =* 3,203) were dissected and 117 (3.7%) of them were found infected with *L. infantum* during the three years of the study. In 2012 we dissected 735 females and 19 (2.6%) of them were found infected. In 2013 we dissected 864 females and 57 (6.6%) of them were infected. In 2014 we dissected 1,604 females and 41 (2.6%) of them were infected. All isolates were characterized by PCR of ITS regions as *L. infantum*. Furthermore, 13.31% and 7.78% of blood-fed and unfed females respectively were found to be infected with *L. infantum* by PCR examination. Blood-meal identifications were conducted with females collected with both sticky and CDC traps. Amplification of a fragment of 359 bp of vertebrate cytochrome-*b* gene, sequencing and comparison with sequences deposited in GenBank was performed. The analysis of blood preferences revealed that sand flies feed mainly on rabbits (*Oryctolagus cuniculus*) follow by hares (*Lepus granatensis*). Characterization of promastigotes isolated from sand flies fed on both lagomorphs proved that were infected by *L. infantum*. These data are in concordance with results obtained from xenodiagnostic studies in hares and wild rabbits from the focus. In conclusion, the present entomological study highlights the exceptional nature of the *Leishmania* outbreak occurring in southwest Madrid region. It is confirmed that *Ph. perniciosus* is the only vector in the area and that it is present in high densities with high rates of infection. Rabbits and hares were the main blood sources for this sand fly species. These results reinforce the need for an extensive and permanent surveillance in this region, and others of similar characteristics, in order to control vectors and wild reservoirs.

Financial support: In part by FP7-UE EDENext, grant 261504.

**Molecular tools for the identification of phlebotomine sand flies and detection of *Leishmania* spp. parasites in Misiones province, Argentina**

Sofía L. Moya^1,2^, Magalí G. Giuliani^1^, Mariana Manteca Acosta^1^, Oscar D. Salomón^1,2^, Domingo J. Liotta^1,3^

^1^Instituto Nacional de Medicina Tropical, Ministerio de Salud de la Nación (INMeT-MSAL), Puerto Iguazú, Misiones, Argentina

^2^Consejo Nacional de Investigaciones Científicas y Técnicas (CONICET), Argentina

^3^Laboratorio de Biología Molecular Aplicada (LaBiMAp-FCEQyN-UNaM), Posadas-Misiones-Argentina

sofialorian@gmail.com; odanielsalomon@gmail.com

In Argentina, Tegumentary Leishmaniasis (TL) is endemic in nine provinces, while the number of Visceral Leishmaniasis (VL) cases have increased sharply in the last years and expanded to new areas, Misiones province having the highest incidence. Because the disease spread depends largely on the geographical distribution of the vectors, simultaneous characterization of vectors and parasites using molecular tools will contribute to the eco-epidemiological knowledge of this disease through a simplified experimental approach. Procedures for sand fly identification based on morphology often alter the nature of the sample and inhibit subsequent analysis by molecular methods, such as those required for detection and identification of parasites. In this context, the aim of this work was evaluate two protocols: PCR-Sequencing of cacophony-IVS6, as a complementary method in the taxonomic identification of sand flies, and PCR-RFLP of *ITS-1* for detection and identification of *Leishmania* spp. Female sand flies were captured with light traps and identified to species level by dissection and examination of the morphology of the spermathecae according to available taxonomic keys. Sand fly identification also was carried out through DNA extraction of the remaining sand fly body, followed by amplification and sequencing of cac-IVS6. After the evaluation and alignment of sequences, datasets were analyzed by Neighbor-Joining (NJ) using the Kimura 2 parameters model. Detection and identification of *Leishmania* parasites was done according to the *ITS-1* PCR-RFLP protocol in 39 of the blood-fed captured females. Amplification and sequencing of cac-IVS6 was achieved in 107 female sand flies. The multiple alignment revealed a conserved exon and a polymorphic intragenic region. NJ consensus tree showed that the intron polymorphism is sufficient for differentiation of sequences belonging to sand flies of different genera (*Lutzomyia*, *Nyssomyia*, *Migonemyia* and *Evandromyia*). Sequences belonging to the same genus (*Ny. whitmani* and *Ny. neivai*, both proven to be vectors of *L. braziliensis*) clustered together making differentiation between species impossible. Further, the protocol of cac-IVS6 allowed the identification of four sand flies whose identities could not be achieved by morphological means due to the condition of the specimens. Detection of *Leishmania* parasites was possible in 15% of the samples (6/39), but the results of the RFLP assay for species identification was different than expected. Because of this, ITS-1 products were sequenced and analyzed for identity with BLASTn, resulting in six sequences of *L. infantum* (detected in *Ny. whitmani* and *Mg. migonei* sand flies). In conclusion, even though a sequencing protocol was required for *Leishmania* species identification, both protocols were applied in the same biological sample with positive results, allowing simultaneous identification of sand fly and the parasite present. It is important to note that the detection of *Leishmania* DNA itself is not enough to declare a sand fly species a vector of *Leishmania*, although these findings may serve as a first step to define the direction of future eco-epidemiological studies in Misiones province.

## Epidemiology and control (oral communications)

**Keynote – Can *Sergentomyia* spp. play a role in the transmission of human and animal leishmaniases?**

Carla Maia

Global Health and Tropical Medicine, Medical Parasitology Unit, Instituto de Higiene e Medicina Tropical, Universidade de Nova Lisboa, Portugal

carlamaia@ihmt.unl.pt

Leishmaniases are parasitic diseases caused by protozoa of the genus *Leishmania*. The parasites, which infect various wild and domestic mammals, are usually transmitted by the bite of phlebotomine sand flies belonging to the genus *Phlebotomus* (in the Old World) or *Lutzomyia* (in the New World) genera. On the other hand, *Sergentomyia* sand flies, which are widely distributed throughout the Old World, have been proven as vectors of reptile *Leishmania*. Since it is generally accepted that most species of *Sergentomyia* are not anthropophilic, they are not usually regarded as vectors of infectious agents (including *Leishmania*) to humans. However, based upon literature reviews, a consideration of the role of *Sergentomyia* in the circulation of mammalian *Leishmania* becomes apparent as *Leishmania* DNA has been identified in several species. These include the molecular detection of *L. major* in *Se. sintoni* in Iran, *Se. garnhami* in Kenya, *Se. darlingi* in Mali, and *Se. minuta* in Portugal. Furthermore *L. donovani* has been detected in *Se. babu* in India, *L. infantum* in *Se. dubia*, *Se. magna* and *Se. schewtzi* in Senegal, and *L. siamensis* in *Se. gemmea* in Thailand. Finally, more recently, *L. tropica* has been found in *Se. ingrami* and *Se. hamoni* in Ghana and *Leishmania* sp. related to Chinese *Leishmania* sp. previously isolated from human and canine leishmaniases have been detected in *Se. minuta* in Portugal. Nevertheless, PCR positivity alone should not be used for incrimination of *Sergentomyia* sand flies as *Leishmania* vectors since the detection of DNA does not give any information about the parasites’ viability or the presence as virulent metacyclic promastigotes in these invertebrate vectors. In fact, and although *L. infantum* DNA was detected in *Se. schwetzi* from Senegal, the refractoriness of this African species to some species of *Leishmania* that infect humans (including *L. donovani*, *L. infantum* and *L. major*) has also been recently demonstrated. In any case, the refractoriness of this particular *Sergentomyia* species does not necessarily extend to the whole of the genus. In this line of reasoning, the competence and permissiveness of the different species of *Phlebotomus* to different Old World *Leishmania* has also been observed. Future work must be done to unravel whether any *Sergentomyia* spp. fulfills all the criteria that will undoubtedly incriminate it as a vector for Old World *Leishmania* with medical and veterinarian importance. These include, (i) the isolation and/or genetic typing of parasites from several unambiguously identified wild female flies not containing blood meals; (ii) the detection of infective forms of *Leishmania* on naturally infected females and/or on colonized sand flies infected experimentally; (iii) demonstration of any biting activity on human reservoirs and (iv) experimental demonstration of vectorial competence for transmission of parasites as a result of blood-feeding on a mammal.

**Molecular analysis of parasite, vector and blood meal DNA from field-caught sand flies in a Moroccan focus of cutaneous leishmaniasis: Genetically heterogenous *Leishmania tropica* in *Phlebotomus sergenti* as a mono-specific and multi-host feeding vector**

Malika Ajaoud^1,2^, Nargys Es-Sette^1^, Rémi N. Charrel^3^, Abderahmane Laamrani-Idrissi^4^, Myriam Riyad^2,5^, Meryem Lemrani^1^

^1^Laboratoire de Parasitologie et Maladies Vectorielles, Institut Pasteur du Maroc, Casablanca, Morocco

^2^Centre d’Études Doctorales des Sciences de la Santé, Faculté de Médecine et Pharmacie, Casablanca Morocco

^3^Aix Marseille University, IRD French Institute of Research for Development, EHESP French School of Public Health, EPV UMR_D 190 ^“^Émergence des Pathologies Virales”, & IHU Méditerranée Infection, APHM Public Hospitals of Marseille, Marseille, France

^4^Service de Parasitologie, Direction d’Épidémiologie et de Lutte contre les Maladies, Ministère de la Santé, Rabat, Morocco

^5^Équipe de Recherche sur les Leishmanioses Cutanées, Faculté de Médecine et Pharmacie, Casablanca, Morocco

meryem.lemrani@pasteur.ma

*Phlebotomus* (*Paraphlebotomus*) *sergenti* is at least one of the confirmed vectors for the transmission of cutaneous leishmaniasis caused by *Leishmania tropica* and is distributed widely in Morocco. This form of leishmaniasis is considered largely to be anthroponotic, although dogs found infected with *L. tropica*, suggest that it is zoonotic in some rural areas. This survey aimed at (i) studying the presence of *Leishmania* in field caught *Ph. sergenti*, (ii) investigating genetic diversity within *L. tropica* and (iii) identifying the host-blood feeding preferences of *Ph. sergenti*. A total of 4,407 sand flies were collected in three rural areas of Azilal province, using CDC miniature light traps. Samples collected were found to consist of 13 species: *Phlebotomus* spp. and 3 *Sergentomyia* spp. The most abundant species was *Ph. sergenti*, accounting for 45.75% of the total. 965 female *Ph. sergenti* were screened for the presence of *Leishmania* sp. by ITS1-PCR-RFLP, giving a positive rate of 5.7% (55/965), all being identified as *L. tropica*. Nucleotide heterogeneity of PCR-amplified ITS1-5.8S rRNA gene-ITS2 was noted. Analyses of 31 sequences obtained segregated them into 16 haplotypes, of which 7 contained superimposed peaks at certain nucleotide positions, suggestive of heterozygosity. *Ph. sergenti* was found to have fed on a large variety of vertebrate hosts, as determined by cytochrome-B sequencing of the DNA from the blood meals of 64 engorged females. Our findings supported the notion that *Ph. sergenti* is the primary vector of *L. tropica* in this focus, and that the latter is genetically very heterogeneous. Furthermore, our results might be suggestive of a certain level of heterozygosity in the *L. tropica* population. This finding, as well as the feeding of the vectors on different animals are of interest for further investigation.

**Sand flies abundance, ecology and oviposition preferences in Bihar, India**

Rajesh B. Garlapati^1^, Shanta Mukherjee^1^, Rahul Chaubey^1^, Tahfizur Rahaman^1^, Piyoosh Babele^1^, Akanksha Chowdhury^1^, Suman Prakash^1^, Vinod Kumar^1^, Mukesh Kumar^1^, Gregory Franckowiak^2^, Dan Somers^2^, Lindsay Briley^2^, Katelyn Wagner^2^, Jenna Hulke^2^, McCall Calvert^2^, Larisa Polyakova^2^, David Poche^2^, Richard Poche^2^

^1^Genesis Laboratories India Private Limited, Patna, Bihar, India

^2^Genesis Laboratories Inc. Wellington, Colorado, USA

rajesh@genesislabs.com

Visceral leishmaniasis (VL) also known as Kala-azar in India, is transmitted to man by *Phlebotomus argentipes*. VL is endemic in the Indian State of Bihar with an estimated death rate of 20,000–40,000 per year. Little is known about sand fly behavior, ecology and oviposition, thus a study into sand fly behaviour and to identify potential oviposition sites was initiated in two villages in Saran district of Bihar state. CDC light traps were installed to monitor sand flies in vegetation and in palm trees and emergence traps were set over assumed oviposition sites. Another study was initiated in twenty four villages, in two districts of Bihar, to study the abundance of sand flies. In each village twelve traps were installed in three types of locations; houses, cattle dwellings and vegetation. In each location type 4 traps were installed. Traps were activated every two weeks. Trapped sand flies were transferred to the lab in Patna and species identity recorded. Results from both studies indicated that *Ph. argentipes* are prevalent in peridomestic habitats and are exophilic in behavior. These studies also indicated a different dimension to sand fly dispersal in India and suggest that new control measures should be explored in addition to current indoor residual spraying to control VL cases in India.

**Keynote – Phlebotomine flies vectors of arbovirus: review and recent data**

Rémi N. Charrel

UMR “Émergence des Pathologies Virales” (EPV: Aix-Marseille Universite – IRD 190 – Inserm 1207 – EHESP), Marseille, France & Institut hospitalo-universitaire Méditerranée infection, APHM Public Hospitals of Marseille, Marseille, France

remi.charrel@univ-amu.fr

Seventy three years ago, the causative agents of sand fly fevers were documented by Albert Sabin from blood samples collected in acutely ill US soldiers after landing in Sicily, Italy, 1943. Naples and Sicilian viruses were causing the same disease although belonging to distinct antigenic complexes. From WWII to 1970’s several viruses were isolated from sand flies in the Old World. All these viruses were distributed into 3 serocomplexes. During the last decade, new technologies as NGS has allowed full genome sequencing for all sand fly-borne phleboviruses and genetic characterisation has prevailed over serology; however, recent studies plead for resurrection of serology as a pivotal tool for integrative and translational research activities. Multidisciplinary research projects are the inescapable way to study viruses transmitted by sand flies. Success rates of isolation of viral strains have placed field work as a priority when investigating new territories far beyond the classic seroepidemiological approaches. Recent results described by several teams will be presented and the points of interest for entomologists will be presented and discussed. The last decade has provided exciting results and innovative scientific approaches that demonstrate that collaborations between entomologists, ecologists, veterinarians, infectious diseases specialists, virologists, and parasitologists is the best manner to unravel the importance of microbes transmitted by sand flies.

**Sand fly fever in Iran: from the past up to the isolation of Dashli virus (a new Sicilian like virus)**

Vahideh Moin-Vaziri^1^, Cigdem Alkan^2^, M. Badakhshan^3^, N. Rahbarian^1,4^, Xavier de Lamballerie^2^, Rémi N. Charrel^2^

^1^Department of Medical Parasitology and Mycology, School of Medicine, Shahid Beheshti University of Medical Sciences, Tehran, Iran

^2^UMR “Émergence des Pathologies Virales” (EPV: Aix-Marseille University – IRD 190 – Inserm 1207 – EHESP) & Fondation IHU Méditerranée Infection, APHM Public Hospitals of Marseille, Marseille, France

^3^Department of Medical Entomology and Vector Control, School of Public Health and Institute of Public Health Research, Tehran University of Medical Sciences, Tehran, Iran

^4^Cellular and Molecular Biology Research Center, Shahid Beheshti University of Medical Sciences, Tehran, Iran

vmvaziri@gmail.com; v.vaziri@sbmu.ac.ir

Phlebotominae sand flies are the vectors of phleboviruses such as Toscana, sand fly fever Sicilian and sand fly fever Naples, which circulate at high rates in Mediterranean countries and can cause human disease. Recent studies have shown a massive increase of novel phleboviruses combined with an expanding geographic distribution of existing phleboviruses in the Mediterranean area and in the Middle-East. In this context, there is an urgent need to extend our knowledge of sand fly fever in Iran. Different sand fly fever viruses such as; Sicilian virus, Karimabad, Salehabad, Tehran were reported by pioneer researchers such as Professors Tesh, Javadian and Saidi since 1977. They declared that *Phlebotomus papatasi* is the main vector in Iran. We conducted this study to update the information that we have about circulation of viruses among sand flies in Iran. Sand flies were collected using CDC light traps and by aspiration from different cities and villages of 10 different provinces of Iran. Each specimen was identified at species level by using reliable morphology keys. Sand flies were pooled based on sex, trapping methods and species, then were sent to the French EPV laboratory in Marseille for virus detection and isolation. A total of 4,770 sand flies (3,158 females and 1,162 males) were collected during 2009–2011 and pooled in 315 groups. The most prevalent species collected was *Ph. papatasi* (57.57%) followed by *Sergentomyia* spp. (31.05%). Also collected were *Ph. alexandri*, *Ph. tobbi*, *Ph. mongolensis*, *Ph. sergenti*, *Ph caucasicus*, *Ph. major* s.l., *Ph. bergeroti* and *Ph. kandelakii*. RT-PCR revealed that a pool which consisted of 30 *Sergentomyia* spp., collected from Dashliboroun village, Golestan province was positive with the primer pair N-Phlebo 1^+^/1^−^. Sequence results showed proximity with sand fly fever Turkey virus (Accession number: GQ847513). Real-time PCR also showed a positive result for another pool of 30 *Ph. papatas*i that were trapped at the same location and on the same day. The novel isolate was named Dashli virus. Further studies are needed to clarify the circulation of Dashli in nature and its potential pathogenicity for human and animals.

**Sand fly fauna of Palmas, state of Tocantins, Brazil: occurrence in different environments and natural infection by trypanosomatids**

Tâmara Dias Oliveira Machado^1,3^, Tauana de Sousa Ferreira^1^, Alcinei de Souza Santos Junior^3^, Nathyla Morgana Cunha Sales^3^, Renata Velôzo Timbó^1^, Tamires Emanuele Vital^2^, Thaís Tâmara Castro Minuzzi-Sousa^1^, Andrey José de Andrade^1,5^, Marcos Takashi Obara^4^, Rodrigo Gurgel-Gonçalves^1^

^1^Laboratório de Parasitologia Médica e Biologia de Vetores, Área de Patologia, Faculdade de Medicina, Universidade de Brasília, Brasil

^2^Laboratório Interdisciplinar de Biociências, Área de Patologia da Faculdade de Medicina, Universidade de Brasília, Brasil

^3^Coordenação de Ciências Matemáticas e Naturais, Instituto Federal de Educação, Ciência e Tecnologia do Tocantins, Palmas, Brasil

^4^Faculdade de Ceilândia da Universidade de Brasília, Ceilândia, Distrito Federal, Brasil

^5^Departamento de Patologia Básica, Setor de Ciências Biológicas, Universidade Federal do Paraná, Brasil

machadoto@ifto.edu.br

The present study aimed to analyze the occurrence of sand flies in gallery forests and housing units (HUs) in Palmas municipality, state of Tocantins, Brazil, and to evaluate their natural infection by trypanosomatids. Four gallery forests and four adjacent domiciliary areas (10 HUs in each) were sampled. In each area 20 HP light traps were placed for three consecutive days. Captures with Shannon traps (3 h) were also performed in forests. Captures were carried out in July (dry season) and November (rainy season) of 2014. The total sampling effort was 960 HP-trap nights and eight Shannon trap sessions. After identification, females captured in the dry season were grouped for DNA extraction. Integrity of the samples was checked by a PCR designed to amplify the cacophony gene IVS6 region in sand flies. Trypanosomatid detection was performed by amplifying the SSU rDNA region. Positive samples were purified, sequenced and compared with sequences deposited at GenBank. A total of 1,527 sand flies representing 30 species was captured: 949 (30 spp.) and 578 (22 spp.) specimens in July and November, respectively. *Nyssomyia whitmani* was the most common species captured (*n =* 743), followed by *Evandromyia carmelinoi* (*n =* 111), *Bichromomyia flaviscutellata* (*n =* 111), and *Psathyromyia hermanlenti* (*n =* 102). In July a greater number of specimens was captured in gallery forests (*n =* 762, 80%), mostly *Ny. whitmani*. In November, most specimens were found in HUs (*n =* 551, 95%). *Ny. whitmani* was the most captured species in HUs (*n =* 336), followed by *Lutzomyia longipalpis* (*n =* 84) and *Ev. carmelinoi* (*n =* 77). *Lu. longipalpis* was found mainly in domiciliary areas (84/86) and *Bi. flaviscutellata* in gallery forests (108/111). DNA was extracted from 569 females and grouped into 78 pools, ranging from 1 to 10 specimens. The nested SSU rDNA PCR identified five positive samples including four *Ny. whitmani* and one *Pintomyia christenseni*, resulting in infection rates of 1.03% and 10%, respectively. Among the four sequences obtained from *Ny. whitmani*, two forest specimens revealed 100% identity with *Leishmania braziliensis*, one HU specimen revealed 100% identity with *Trypanosoma* sp. and the other forest specimen revealed 100% identity with *Crithidia fasciculata* sequence. This is the first record of *C. fasciculata* in *Ny. whitmani*, showing its circulation in Tocantins, and *L. braziliensis* was found in sand flies for the first time in Palmas. The sequence obtained from *Pi. christenseni* from forest revealed 100% identity with *L. braziliensis*. In both periods of the year, sand fly species were collected that have been incriminated as vectors in gallery forests or in the domiciliary areas. However, the highest occurrence of those species occurred during the dry season in gallery forests. Sand flies were more frequent in houses in the rainy season. This was probably due to the moisture from forests that can promote the retention of sand fly populations during the dry season and provide the colonization of adjacent houses in the rainy season.

**First detection of an unknown *Trypanosoma* DNA in a phlebotomine sand fly collected from southern Thailand**

Atchara Phumee^1^, Apiwat Tawatsin^2^, Usavadee Thavara^2^, Theerakamol Pengsakul^3^, Suwich Thammapalo^4^, Jérôme Depaquit^5^, Frédérick Gay^6^, Padet Siriyasatien^1,7^

^1^Department of Parasitology, Faculty of Medicine, Chulalongkorn University, Bangkok, Thailand

^2^National Institute of Health, Department of Medical Sciences, Ministry of Public Health, Nonthaburi, Thailand

^3^Faculty of Medical Technology, Prince of Songkla University, Songkhla, Thailand

^4^The Office of Disease Prevention and Control 12, Songkhla, Thailand

^5^Université de Reims Champagne Ardenne, ANSES, SFR Cap santé, EA 4688 – USC « Transmission Vectorielle et Épidémiosurveillance de Maladies Parasitaires (VECPAR) », Reims, France

^6^Université Pierre et Marie Curie-Paris 6, CHU Pitié-Salpêtrière, AP-HP, Groupe Hospitalier Pitié-Salpêtrière, Service Parasitologie-Mycologie, Paris, France

^7^Excellence Center for Emerging Infectious Diseases, King Chulalongkorn Memorial Hospital, Thai Red Cross Society, Bangkok, Thailand

padet.s@chula.ac.th

Female sand flies are known to be the vectors of *Leishmania* parasites and viruses. Several reports have also shown that these insects can also act as vectors for trypanosomes of bats, lizards and snakes. We report here on the collection of sand fly specimens from southern Thailand and our analysis to determine which species of sand fly were present and whether or not trypanosomes were present. To demonstrate the presence of the trypanosomes in the sand fly we used PCR sets annealed specifically to the internal transcribed spacer 1 (ITS1) and small subunit ribosomal RNA (SSU rDNA) gene of trypanosomatids; in addition, the cytochrome *b* gene was used to identify the species of sand fly. Among 45 samples of sand fly collected, 7 samples were shown to be *Phlebotomus stantoni*, and one of these specimens also amplified positively in the PCR for *Trypanosoma* sp. The study is the first detection of *Trypanosoma* sp. in a sand fly from Thailand. Sequence of the amplified ITS1 and SSU rDNA gene indicated that this Trypanosome is a suspected novel species. Further studies of this suspected new *Trypanosoma* species including identification of the vertebrate host and the potential of this parasite to be pathogenic are necessary.

**Overview and an update of the current knowledge and perspectives on sand fly research in Mexico**

Eduardo A. Rebollar-Téllez^1^, Sergio I. Ibáñez-Bernal^2^, Jorge J. Rodríguez-Rojas^1^, David A. Moo-Llanes^3^, Angélica Pech-May^3^, Ana C. Montes de Oca-Aguilar^2^, Oscar Mikeri-Pacheco^9^, Miriam Berzunza-Cruz^4^, Ingeborg Becker-Fauser^4^, Janine Ramsey^3^, Carlos Ibarra-Cerdeña^5^, Ángel Rodríguez-Moreno^6^, Christopher Stephens^7,8^, Victor Sánchez-Cordero^6^, Alfredo Castillo-Vera^9^, Camila González^10^, Wilfredo Arque-Chunga^1^, Javier Escobedo-Ortegón^11^, Silvia Pasos-Pinto^4^, Laura Sánchez-García^4^

^1^Universidad Autónoma de Nuevo León, Facultad de Ciencias Biológicas, Departamento de Zoología de Invertebrados, Laboratorio de Entomología Médica, Avenida Universidad S/N, Ciudad Universitaria, CP. 66451, San Nicolás de los Garza, Nuevo León, México

^2^Instituto de Ecología, A.C., Red Ambiente y Sustentabilidad, Laboratorio de Sistemática y Ecología de Insectos con Interés Médico y Veterinario, Veracruz, México

^3^Centro Regional de Investigación en Salud Pública (CRISP), Instituto Nacional de Salud Pública (INSP), Tapachula, Chiapas, México

^4^Unidad de Investigación en Medicina Experimental, Facultad de Medicina, Universidad Nacional Autónoma de México (UNAM), Ciudad de México, México

^5^Centro de Investigación y Estudios Avanzados, Instituto Politécnico Nacional, Mérida, Yucatán, México

^6^Instituto de Biología, Universidad Nacional Autónoma de México (UNAM), Ciudad de México, México

^7^Centro de Ciencias de la Complejidad, Universidad Nacional Autónoma de México (UNAM), Ciudad de México, México

^8^Instituto de Ciencias Nucleares, Universidad Nacional Autónoma de México (UNAM), Ciudad de México, México

^9^El Colegio de la Frontera Sur (ECOSUR), Tapachula, Chiapas, México

^10^Universidad de los Andes (UNIANDES), Ciencias Biológicas, Colombia

^11^Centro de Investigaciones Regionales “Dr. Hideyo Noguchi”, Universidad Autónoma de Yucatán, Mérida, Yucatán, Mexico

eduardo.rebollartl@uanl.edu.mx

Cutaneous leishmaniasis is highly endemic in southern Mexico. The first clinical reports were made by Danish physician Harald Seidelin in 1912 and other epidemiological work by Beltran and Bustamante (1942) and a decade later, the outstanding work of Dr. Francisco Biagi, identified the causative parasite as *Leishmania mexicana* (Biagi, 1953). During field studies conducted in Quintana Roo, México, Biagi and his team, were able to show that the sand fly *Bichromomyia olmeca olmeca* was the main vector. During the following years; the knowledge of species distribution and taxonomy increased substantially. To-date a total of 50 phlebotomine sand fly species have been recorded; which include 48 extant species and 2 extinct species. Of these 48 extant species, it is considered that in southern Mexico only four sand fly species may play a significant role as vectors of *L. mexicana*. Over the last years our research group has shown that geographical distribution of leishmaniasis does not correlate with the distribution of the sand fly *Bi. olmeca olmeca*. Furthermore, evidence gathered in the Yucatan Peninsula over the last years, has shown that other sand fly species may also act as vectors. These suspected vectors are *Lutzomyia cruciata*, *Psathryromyia shannoni*, and *Psychodopygus panamensis*. Thus, based on infection and biting rates; it has been hypothesized that transmission of *L. mexicana* in southern Mexico may be because of these four sand fly species. Vector species in Central, Pacific coast and Northern Mexico, may vary as well although these foci are less studied. In northern Mexico we have recorded a total of 11 sand fly species, of which *Lu. diabolica* and *Dampfomyia anthophora* are the most likely vectors. Even though natural infection of sand flies in northern Mexico has not yet been demonstrated, the presence of *L. mexicana* in wild rodents has been detected. Currently alpha diversity studies are underway with the aim of understanding sand fly assemblages and during these studies it has been observed that taxonomical work is still required to describe new species. Quantitative models of alpha and beta diversity in particular sites, has shown that these methods are useful tools to describe richness, abundances, heterogeneities, dominances and evenness. In addition; ecological niche models (ENM) have also been employed to assess how sand fly species distribution may shift under different scenarios of global warming. Overall; the main objective of this work is to revise and present an updated status of sand fly vectors in México.

**Abundance of *Lutzomyia longipalpis* (Diptera, Psychodidae) in a kennel and its surroundings on a highly endemic visceral leishmaniosis area in São Paulo State, Brazil**

Andre A. Cutolo^1^, K.B.S. Briguente^1^, G. Motoie^1^, C.E.J. Pigozzi^2^, B.L. Neves^3^, I. Menz^4^, V.L. Pereira-Chioccola^1^

^1^Instituto Adolfo Lutz, Av. Dr Arnaldo, 351, 8° andar. São Paulo (SP), Brazil

^2^Faculdade de Ciências Agrárias de Andradina, Andradina (SP), Brazil

^3^Universidade do Oeste Paulista, Presidente Prudente (SP), Brazil

^4^Ingrid Menz Micro Empresa, Campinas (SP), Brazil

cutoloandre@yahoo.com

*Lutzomyia longipalpis* is the main vector of *Leishmania infantum*, the etiologic agent of human and canine visceral leishmaniasis (cVL) in the Americas. From 1999 to 2013 there were 192 deaths among 2,204 human VL diagnosed cases, within 76 municipalities while *Lu. longipalpis* was detected in 108 out of 645 cities of São Paulo state, in southeastern Brazil. VL has been expanding its range and number of human and canine cases has been increasing in several parts of South America. Dogs are the main reservoir of the VL causing agent in the urban environment. In order to evaluate aspects of the dynamics and behavior of *Lu. longipalpis* in a highly endemic VL area, sand flies were captured and monitored from January 2012 to September 2013 at a kennel with approximately 100 dogs in the city of Dracena, SP. Automatic light traps were set up in at least eight different points around the kennel area and its’ surroundings during 70 nights on an average of 3.34 nights per month. Automatic aspirators were also used for sand fly capture inside dog-houses from March to September 2013. During the evaluation period a total of 4,439 sand flies were captured with *Lu. longipalpis* representing 4,425 (99.68%) of the captured specimens. *Evandromyia cortellezzii* and *Ev. lenti* were also captured; 13 (0.29%) and 1 (0.02%) individuals respectively. Of the captured sandflies, 2,714 (61.14%), originated from the automatic light traps with the following distribution in decreasing order: 1,265 (46.51%) chicken coop and 968 (35.67%) from the domicile porch of west neighbour farmstead, 133 (4.90%) from the kennel pen, 99 (3.65%) front orchard of neighbouring farmstead, 61 (2.25%) east neighbour backyard, 60 (2.21%) back of kennel main house, 55 (2.03%) front of kennel main house and others 73 (2.69%). Direct aspiration of kennel houses used as shelters by the dogs produced 1,725 (38.86%) of the total number of captured sand flies. Regarding seasonality, the total number of sand flies captured monthly by automatic light traps respectively from January to November 2012 were 302 (17.84%), 282 (16.66%), 160 (9.45%), 427 (25.22%), 102 (6.02%), 59 (3.08%), 42 (2.48%), 106 (6.26%), 70 (4.13%), 101 (5.97%) and 42 (2.48%), and from January to September 2013 were 199 (19.49%), 271 (26.54%), 158 (15.48%), 162 (15.87%), 119 (11.66%), 12 (1.18%), 73 (7.15%), 26 (2.55%) and 1 (0.10%). The higher abundance of sand flies seems to be related to the most hot and humid months and also related to the sites with constant presence of hosts for blood meals. *Lu. longipalpis* was present during all sampled months and trap sites, confirming its high adaptability to the urban and human environment.

**Canine visceral leishmaniasis in the São Paulo metropotian area dissociated of *Lutzomyia longipalpis: Pintomyia fischeri* as potential vector of *Leishmania infantum chagasi***

Fredy Galvis Ovallos, Eunice A.B. Galati

Public Health Faculty, Sao Paulo University, Av Dr. Arnaldo 715, CEP 01246904, São Paulo, SP, Brazil

galvisfregao@gmail.com

Zoonotic visceral leishmaniasis (ZVL) is an important public health problem in Brazil. This disease is caused by infection with *Leishmania infantum* transmitted mainly by the sand fly *Lutzomyia longipalpis*. After the first detection of autochthonous canine visceral leishmaniasis (cVL) cases in São Paulo State in 1998, the enzootic has spread in association with the geographical expansion of *Lu. longipalpis*. In Cotia and Embu das Artes municipalities, in the São Paulo Metropolitan region (São Paulo State), autochthonous canine cases have been identified since 2003 in the absence of *Lu. longipalpis*. However, ecological studies in this area showed that *Pintomyia fischeri* is the predominant species, representing about 90% of the specimens among 13 species captured. This result has led to the suspicion that *Pi. fischeri* could be acting as vector of the VL agent. Other evidence such as the attractiveness of dogs to this species and its blood feeding habits support this hypothesis. In addition, specimens of *Pi. fischeri* captured in another focus of cVL have been found to have *L. infantum* DNA, suggesting that they are naturally infected with this parasite. In xenodiagnoses experiments *Pi. fischeri* females were fed on dogs infected with *L. infantum*, their susceptibility to this parasite and the development of potentially infective promastigotes forms was observed. However, the vectorial competence of *Pi. fischeri* has not yet been demonstrated. These data suggest a potential role for *Pi. fischeri* as a vector *of L. infantum*, however, further studies demonstrating vectorial competence, blood feeding habits in a cVL focus and survival in natural conditions are required.

**The emergence and spread of leishmaniases in the borders of Argentina, Brazil, Paraguay and Uruguay**

Oscar Daniel Salomón^1^, María Gabriela Quintana^1,2^, María Soledad Santini^3^, Nilsa González-Britez^4^, Nidia Martínez^5^, Antonieta Rojas de Arias^6^, Vanete Thomaz-Soccol^7^, André Luiz Gonçalves^7^, Alceu Bisetto Júnior^8^, Gabriela Willat^9^, Luis Calegari^10^, Yester Basmadjian^10^, Zaida E. Yadon^11^, and the IDRC Project #107577 team

Argentina, CONICET: ^1^Inst Nac de Med Tropical-MSN

^2^Inst Sup Entomología-UNT

^3^Centro Nac Diag e Inv Endemoepidemias-ANLIS-MSN, Paraguay

^4^Facultad de Cs de la Salud-Univ Nac del Este

^5^Ministerio de Salud Pública, SENEPA

^6^Centro Desarrollo Inv Científica, Brazil

^7^Dep Engen de Biopr e Biotecnologia, Univ Federal do Parana

^8^Secretaria de Saúde do Estado do Paraná, Uruguay

^9^Ministerio de Salud

^10^Facultad de Medicina, Univ de la República, USA

^11^Comm Dis and Health Anal Department, Pan American Health Organization

odanielsalomon@gmail.com; dsalomon@msal.gov.ar

Since 2014 in the border region of Argentina (Ar), Brazil (Br) and Paraguay (Py), a multi-country project has been carried out to identify the main biological, environmental and social drivers associated with the risk of transmission of leishmaniases, within the theoretical frame of Eco-Health. Uruguay (Uy) joined the project since the emergence of cVL in 2015. The entomological component of the project involves three-night sampling with mini light traps in urban-periurban domestic units, rural-forest and rural-periurban transects by; (a) transverse survey on grids of 400 × 400 m (censal or stratified according to the size of the locality), (b) longitudinal, seasonal/monthly surveys (10% of the original sites). More than 500 sites were sampled among the four countries. The main results so far have shown: (a) the dominance of *Lutzomyia longipalpis* (VL vector) in the urban stratum, both indoors and in peridomestic environments. It is more frequent/abundant in the warmer seasons and places with a tendency to spread to the rural non-sylvatic areas. The dominance of *Nyssomyia whitmani* (CL vector) in the sylvatic-rural stratum, and in “green patches” within urban areas, persistent in colder seasons and places. This spatial segregation was associated with variables that could allow the modelling of the spatial distribution of vectors in time and space at different scales. (b) The evidence-based delimitation of an area for environmental interventions at macrohabitat scale of 400 × 400 m. (c) The genotipification of *Leishmania infantum* and *L. braziliensis* as the main parasites circulating in the region, infecting indoor and outdoor sand flies. In order to avoid averaging very diverse results the actual figures of relative abundance will be shown in tables. However, related to the current spread *Lu. longipalpis* we could distinguish different scenarios: (a) the broad urban distribution of the vector, with cVL prevalence rates of 22–26%, and increasing prevalence during the longitudinal survey: Foz do Iguaçu-Br and Puerto Iguazú-Ar. (b) incipient colonization by *Lu. longipalpis* and 2–4% of cVL: Santa Terezinha de Itaipu-Br and Puerto Libertad-Ar. (c) vectors and cVL restricted to small foci: Presidente Franco-Py (rural-urban) and Ciudad del Este-Py (urban) with 2% cVL prevalence, and three foci in Salto-Uy with cVL rates from 1 to 12% in the main focus. (d) Vectors of CL (*Ny. whitmani* and a lower proportion *Ny. neivai*) without *Lu. longipalpis*, and cVL only from imported cases in transects. (e) In Paysandú-Uy, no vectors were found, so the southernmost border up to now of *Lu. longipalpis*-VL spread is Salto-Uy. The analysis of the entomological component and the integration with the results from other disciplinary components are currently in progress.

Financial support: IDRC-Canada Project #107577, Ministry of Health of Argentina, Brazil, Paraguay and Uruguay; PAHO.

**Evaluation of the synthetic sex pheromone, (*S*)-9-methylgermacrene-B, for recruitment and monitoring of *Lutzomyia longipalpis* (Diptera: Psychodidae) in an environmental reserve in Rio de Janeiro, Brazil**

Vanessa De Araujo Barbosa^1^, Andressa Alencastre Fuzari Rodrigues^1^, James Gordon Campbell Hamilton^2^, Reginaldo Peçanha Brazil^1^

^1^Laboratório Doenças Parasitárias – Instituto Oswaldo Cruz – Fiocruz, Av. Brasil, 4365, Pavilhão Arthur Neiva, 21040-900, Rio De Janeiro, RJ, Brasil

^2^Infectious Disease Transmission and Biology Group, Department of Biomedical and Life Sciences, Faculty of Health and Medicine, Lancaster University, Lancaster, LA1 4YG, UK

vanessabarbosa_bio@hotmail.com

The Serra da Tiririca State Park, located between the Municipalities of Niterói and Marica in Rio de Janeiro State was created by a community movement to protect remnants of Atlantic Forest that were threatened by large scale real estate speculation and intense tourism in the region. Around the park, cases of cutaneous leishmaniasis occur sporadically near residential areas and *Lutzomyia longipalpis*, the main vector of *Leishmania infantum*, is found in the park. The vector is considered a species complex because of the different sex pheromones that are produced by the members of the complex. As pheromones are considered to be non-toxic substances, they can have an important role in monitoring and vector control. *Lu. longipalpis* populations are frequently sampled by light trap, Shannon trap and others, but collections may represent only a small proportion of the actual population because of inefficiencies in the sampling method. In forested areas, such as the Serra da Tiririca, the number of sand flies captured with CDC light traps is very low, in 8 years of trapping using miniature CDC light traps 8 male and 4 female *Lu. longipalpis* were captured suggesting that in this park the numbers of *Lu. longipalpis* are low. The main species trapped were *Evandromyia tupynambai* (451), *Brumptomyia cunhai* (172), *Micropygomyia schreiberi* (128) and *Br. nitzulescui* (95). However sampling methodolgy could mask the real situation with sand fly abundance and thus the transmission cycle in this region. Thus our objective was to evaluate the synthetic pheromone (*S*)-9-methylgermacrene-B as a component of a strategy for monitoring *Lu. longipalpis* in a wild environment, where their presence is currently considered to be rare. In this study male and female *Lu. longipalpis* were collected with miniature CDC type traps fitted with either lights and synthetic pheromone (test) or light only (control). Test and control traps were placed 5 m apart. GC/MS analysis confirmed that the population of *Lu. longipalpis* in the area was (*S*)-9-methylgermacrene-B. In six trapping nights over 3 weeks, where the position of test and control traps was alternated on each subsequent night, we observed a significant difference between the number of *Lu longipalpis* captured in the test traps (synthetic pheromone present) (mean = 127.2 ± 37.6) compared to those captured using only light attraction (mean = 17.7 ± 4.6), (Paired *T*-test; *P* = 0.022). We concluded that synthetic sex pheromone (*S*)-9-methylgermacrene-B is a valuable tool for monitoring *Lu. longipalpis* and possibly as a component of a new control strategy for this species.

**Synthetic pheromone and long lasting insecticidal nets (LLINs) as a new control strategy for *Lutzomyia longipalpis* (Diptera: Psychodidae), the vector of *Leishmania* (*Leishmania*) *infantum***

Vanessa De Araujo Barbosa^1^, Cristian Ferreira De Souza^1^, James Gordon Campbell Hamilton^2^, Reginaldo Peçanha Brazil^1^

^1^Laboratório Doenças Parasitárias – Instituto Oswaldo Cruz – Fiocruz, Av. Brasil, 4365, Pavilhão Arthur Neiva, 21040-900, Rio De Janeiro, RJ, Brasil

^2^Infectious Disease Transmission and Biology Group, Department of Biomedical and Life Sciences, Faculty of Health and Medicine, Lancaster University, Lancaster, LA1 4YG, UK

vanessabarbosa_bio@hotmail.com

*Lutzomyia longipalpis*, the main vector of *Leishmania infantum*, is considered to be a species complex and different members of the complex produce different pheromones. Current strategies for controlling sand flies using residual spraying have been unable to prevent the spread of the American visceral leishmaniasis (AVL) across Brazil. Because of this, new cost-effective approaches are urgently needed to manage populations of the sand fly vector. Long lasting insecticidal nets (LLINs) offer a new approach to vector control because they could be an alternative to residual spraying at *Lu. longipalpis* aggregation sites, such as chicken sheds. The synthetic sand fly pheromone, (*S*)-9-methylgermacrene-B, can attract *Lu. longipalpis* in natural environments. Here, we tested an “attract-and-kill” strategy for improving *Lu. longipalpis* control by using the synthetic pheromone in conjunction with LLINs. This study was conducted in Governador Valadares, Minas Gerais, an area considered endemic for AVL in Brazil. Field experiments were performed with experimental chicken sheds baited with synthetic sex pheromone to compare the use of LLIN with conventional insecticide spraying on *Lu. longipalpis* mortality. Our results demonstrated that the addition of synthetic pheromone resulted in greater numbers of male and female sand flies being caught at experimental chicken sheds. This study showed that both treatments (residual insecticide and impregnated netting) killed close to 100% of sand flies in 24 h following exposure. After 2 months the lethal effect of both treatments was maintained at close to 100%. After 4 months of exposure treatments, the lethal effect of netting diminished to approximately 69% and residual insecticide to 89%. We concluded that insecticide impregnated netting has potential as a tool for killing sand flies at aggregation sites, and synthetic pheromone can increase their effectiveness attracting more sand flies to be killed by the netting. More long-term field trials are needed to improve the effectiveness of the netting and identify the feasibility of treating surfaces with impregnated netting as part of visceral leishmaniasis control program.

**Identifying the yeast community in the sand fly *Phlebotomus perniciosus:* towards a strategy for yeast-mediated biological control of vector-borne diseases**

Elena Martin^1^, Ilaria Varotto Boccazzi^1^, Gioia Bongiorno^2^, Leone De Marco^3^, Luigi Gradoni^2^, Nicoletta Basilico^4^, Stefano Comazzi^1^, Irene Ricci^3^, Sara Epis^1^

^1^University of Milan – Department of Veterinary Medicine, Italy

^2^Istituto Superiore di Sanità – Unit of Vector-Borne Diseases and International Health, Italy

^3^University of Camerino – School of Biosciences and Veterinary Medicine, Italy

^4^University of Milan – Department of Biomedical Sciences, Surgical and Dental, Italy

sara.epis@unimi.it

Vector-borne diseases (VBDs) are one of the greatest public health problems worldwide and their control represents a key global public health challenge for the 21st century. In particular leishmaniases are considered a worldwide re-emerging health problem because they are spreading and this may be caused by climate and environmental changes. In this context, the increased awareness of the environmental and the public health problems caused by the excessive and uncontrolled use of insecticides to combat VBDs is leading to the development of alternative control strategies. While bacteria associated with arthropods are broadly studied and are the focus of several research programs aimed at developing strategies to control VBDs, such as malaria, dengue, and trypanosomiasis, arthropod-associated yeasts and their possible application in biocontrol have not yet been intensely investigated. The objective of this work was to study the yeast community associated with the sand fly *Phlebotomus perniciosus*, the main vector of leishmaniasis in the western Mediterranean area, with the aim of investigating their potential to interfere with *Leishmania* development in the insect. To reach this goal we associated culture-based methodology with culture independent methods: we performed yeast isolation and identification, 454 pyrosequencing, PCR screening and whole mount FISH with specific probes to identify the yeast species. We isolated the yeast *Wickerhamomyces anomalus*, a species known for its potential in biocontrol, from both male and female *Ph. perniciosus*. This yeast was phylogenetically characterized and then tested against toxin-susceptible yeast strains, demonstrating its’ killer phenotype. Finally, in order to explore the possibility that this yeast could exert inhibitory/killing activity against pathogens, we tested the *in vitro* activity of *W. anomalus* strains against the pathogen *Leishmania infantum.* This study offers the basis for the development of an environmentally-friendly and human health safe method for VBD control that could be included in an integrated leishmaniasis control program.

**Targeting sand fly control by the use of systemic insecticides presented to mammalian reservoir hosts of ZCL and VL: A review of recent studies**

Richard M. Poché^1^, Daniel Hartman^1^, Larisa Polyakova^1^, Rajesh Babu Garlapati^2^, David Poché^1^

^1^Genesis Laboratories, Inc., Wellington, Colorado – 80549, USA

^2^Genesis Laboratories India Private Limited, Patna, India

richard@genesislabs.com

Zoonotic cutaneous leishmaniasis (zCL) and visceral leishmaniasis (VL) are diseases transmitted by phlebotomine sand fly vectors throughout much of the world. Control of the diseases is most practical by attempting to reduce the sand fly numbers using available methods. Studies over the past 20 years have demonstrated good control of sand flies by treating the reservoir hosts of zCL and VL with systemic insecticides. Both larval and adult sand fly numbers may be greatly reduced through the use of such products. Studies from India, Kazakhstan, Tunisia, and the USA conclude that the oral treatment of animals with insecticides such as imidacloprid and fipronil can greatly reduce these vector numbers. We present a review of data collected, along with recent research, that show efficacy against the vector as high as 98% by treating animals such as Norway rats, great gerbils, bandicoot rats, fat sand rats and cattle to control sand fly numbers. Additional data have been compiled to show similar efficacy results with other vectors, including mosquitoes and ticks.

**Systemic insecticides used in dogs: potential candidates for sand fly control?**

Sonia Ares Gomez, Albert Picado

Barcelona Institute for Global Health, Hospital Clinic – Universitat de Barcelona Roselló 132, Barcelona, Spain

sonia.ares@isglobal.org

Phlebotomine sand flies are vectors of zoonotic visceral leishmaniasis (ZVL), a disease in humans and domestic dogs caused by the protozoan, *Leishmania infantum*. Dogs, the main reservoir, are required to maintain transmission. Interest in sand fly control has been increasing in the past few decades because ZVL is adapting to new environments and spreading to new geographical areas. New strategies to prevent human cases include the protection of dogs with insecticides. Insecticide impregnated collars, for instance, have shown to protect dogs from sand fly bites and also to reduce the risk of human leishmaniasis. But the use of collars poses logistic challenges and may not be cost-effective in ZVL endemic areas. Thus new sand fly control strategies should be evaluated. The use of systemic insecticides in dogs has been postulated as an alternative or complement to current vector control methods. The objective of the study is to find systemic insecticides in dogs that could be used in sand fly control. We have reviewed the systemic insecticides currently used in dogs and we have assessed their potential use as sand fly control tools. The search was made in 4 steps. First, we listed the systemic drugs used in dogs with endo- and ectoparasiticide activity from the following sources: small animal formulary of the British small animal veterinary association (BSAVA), Vademecum veterinario (Guíavet), and the web page parasitipidia.net. Second, using the European Medicines Agency and U.S. Food and Drug Administration web pages we looked for information such as product indications, clinical pharmacology, mechanism of action, duration of action, and efficacy of the systemic drugs selected in the first step. Third, using PubMed.gov we reviewed the research published about the active ingredients and their potential use in sand fly control. Fourth, we ranked these systemic drugs based on following criteria: (1) evidence about the product capability of killing sand flies, (2) optimal plasma concentration levels in dogs to kill sand flies and (3) at least one month of expected efficacy. A total of 13 commercial products and 10 systemic drugs were identified in the first three steps. Four of the commercial products were a combination of two systemic drugs. After ranking the products only five drugs (spinosad, fluralaner, afoxolaner, sarolaner, and moxidectin) were included in the list of potential systemic insecticides in dogs to control sand flies. The efficacy of those drugs as sand fly control measures should be evaluated in laboratory and field studies. In order to have an impact on *L. infantum* transmission, thus on the risk of ZVL in humans, these drugs should have a long-lasting efficacy against sand flies and should be easy to administer (e.g. ideally per os). Mathematical modeling can help estimate the efficacy of this potential new vector control strategy in ZVL endemic areas.

**Repellent efficacy of a new combination of fipronil and permethrin against the main vector of canine visceral leishmaniasis in the Americas (*Lutzomyia longipalpis*)**

Andre A. Cutolo^1^, Fredy Galvis Ovallos^2^, E.S. Neves^3^, S. Sossai^3^, M.M.F. Vieira^3^, F.O. Silva^1^, S.T. Chester^4^, B. Fankhauser^4^, M.D. Soll^4^

^1^Merial Saúde Animal, Fazenda S. Francisco s/n°, Paulínia (SP) – 13140-970, Brazil

^2^Universidade de São Paulo, Faculdade de Saúde Pública, Av. Dr Arnaldo, 715, São Paulo (SP) – 01246-904, Brazil

^3^Nowavet. R. Joaquim Lopes de Faria, 630, Viçosa (MG) – 36570-000, Brazil

^4^Merial Limited, 3239 Satellite Blvd. Duluth, GA – 30096, USA

cutoloandre@yahoo.com

A study was conducted to evaluate the efficacy of a new fipronil and permethrin combination to repel *Lutzomyia longipalpis* sand flies when applied once topically on dogs. The study was conducted using 16 beagle dogs and laboratory reared *Lu. longipalpis* sand flies. Eight non-treated dogs were compared to eight dogs treated with a combination containing 67.6 mg/mL fipronil + 504.8 mg/mL permethrin (Frontect^®^/Frontline Tri-Act^®^, Merial). The treatments were applied topically once on Day 0. Each dog was exposed to approximately 80 *Lu. longipalpis* female sand flies on Days 1, 14, 21 and 30. Percent sand fly repellency on treated dogs was 95.7%, 94.3%, 81.7% and 72.2%, for Days 1, 14, 21 and 30, respectively. There was a significant difference (*P* ≤ 0.016) between the treated and control groups on every assessment day. A single topical administration of a new combination of fipronil and permethrin demonstrated a significant repellent effect (i.e. >80%) for 21 days against *Lu. longipalpis*. The results suggest that treatment with the fipronil-permethrin combination could be integrated into canine visceral leishmaniasis prevention and control programs in endemic countries in the Americas.

**Molecular and biochemical characterization of insecticide resistance in *Phlebotomus* and *Lutzomyia* sand flies**

Scott A. Bernhardt, David S. Denlinger, Zachariah Gompert, Joseph S. Creswell

Utah State University, Department of Biology, 5305 Old Main Hill, Logan, Utah 84341, USA

scott.bernhardt@usu.edu

Identification of phenotypic traits associated with insecticide resistance in phlebotomine sand flies is an essential component of vector control programs. Chemical insecticides continue to be a critical tool of control programs in an effort to mitigate the spread of leishmaniasis. Sand flies have been exposed for decades to a variety of insecticide classes, which has increased evolutionary selective pressures of resistance. In many insects, resistance arises either from target-site insensitivity and/or metabolic detoxification. Target-site insensitivity involves single nucleotide polymorphisms (SNPs) in the paralytic (*para*) and acetylcholinesterase-1 (*ace-1*) genes, which have been implicated in conferring insensitivity to pyrethroids and organophosphates. Metabolic resistance involves changes in the expression of carboxylesterases, cytochrome P450’s, and glutathione S-transferases that are capable of binding, sequestering, metabolizing, and conjugating insecticides. The primary objective of this research was to augment the understanding of target-site insensitivity and metabolic detoxification at the molecular level for pyrethroid and organophosphate resistance in *Phlebotomus papatasi* and *Lutzomyia longipalpis*. We are in the process of characterizing *para*, *ace-1*, and protein profiles in insecticide-susceptible and -resistant *Ph. papatasi* and *Lu. longipalpis* laboratory colonies. To do so, we initially determined the diagnostic doses and times for *Ph. papatasi* and *Lu. longipalpis* sand flies using the CDC bottle bioassay with 10 different insecticides covering four insecticide classes. Both species were determined to be highly susceptible to DDT and carbamates, whereas with other insecticides, these species varied substantially in their diagnostic dose and times. In addition, we have exposed separate *Ph. papatasi* and *Lu. longipalpis* colonies to permethrin (pyrethroid) and malathion (organophosphate) over multiple generations to induce resistance. At a 60% malathion lethal concentration exposure, F_7_ generation *Ph. papatasi* are demonstrating 87.8% survival compared to a 58.7% survival in F_5_ generation *Lu. longipalpis*. At a 60% permethrin lethal concentration exposure, F_16_ *Ph. papatasi* demonstrate 79.6% survival compared to a 77.1% survival in F_9_ generation *Lu. longipalpis*. Baseline molecular and biochemical characterization have also been completed in the susceptible colonies. DNA sequence has been recovered surrounding *para* 1,014th (*kdr*), 918th super-*kdr* region, *ace-1* 119th codons, and upstream (5′) and downstream (3′) regions from these codons in the permethrin- and malathion-resistant-selected *Lu. longipalpis* and *Ph. papatasi* colonies. Initial sequence data from the F_3_ generation of the *Lu. longipalpis* permethrin-resistant-selected colony and from the F_3_ generation of the *Lu. longipalpis* malathion-selected colony has revealed no SNPs in the *para* and *ace-1* genes. Initial sequence data from the F_4_ generation of the *Ph. papatasi* permethrin-resistant-selected colony and from the F_3_ generation of the *Ph. papatasi* malathion-resistant-selected colony has revealed no SNPs in the *para* and *ace-1* genes. We have begun to conduct genotyping-by-sequencing, utilizing an Illumina sequencing platform, to identify genome-wide SNP markers associated with insecticide resistance in *Ph. papatasi* and *Lu. longipalpis*. Our working hypothesis is that insecticide resistance can potentially be more diverse than a single SNP or enzyme class and that resistance may be polygenic with many genes contributing to the resistance phenotype. Results from these analyses will provide significant insights into understanding the mechanisms of insecticide resistance in field-collected sand flies where leishmaniasis is a serious public health threat.

**Evaluation of the spatial relationship between area of insecticide treatment and location of Leishmaniasis cases using geographical information systems in Adana, Turkey**

Hakan Kavur^1^, Ozan Artun^1^, Kenan Koca^2^

^1^Cukurova University, Karaisalı Vocational School, Adana, Turkey

^2^Dicle University, Faculty of Agriculture, Department of Soil and Plant Nutrition, Diyarbakır, Turkey

hakankavur@yahoo.com

Insecticide spraying was conducted to control arthropod vector populations, especially sand flies and mosquitoes, in 103 districts in downtown Adana, after people had reported their mosquito and sand fly problems to the metropolitan municipality. People, from 103 locations telephoned the municipality 5,656 times in 2014. Leishmaniases are present in Turkey in two clinical forms; visceral and cutaneous, and show a tendency for spreading throughout the country. Numbers and distribution of cutaneous leishmaniasis cases in Adana province were obtained from the Turkish Ministry of Health. The aim of the present study was to compare the relationships between the locations of leishmaniasis patients and insecticide spraying area by using the data in selected study sites in Adana province. In this regard, city borders were drawn using ARCMAP10.0. Additionally the map was separated into different layers for querying each province independently. Altitude, leishmaniasis case numbers and insecticide application numbers of the selected locations were entered into the database and relationships between them were evaluated. Identification of the correlation levels of these data may provide useful information to better guide control program interventions.

**Manipulation of sand fly distributions within the peridomestic environment, and implications for the control of vector borne disease**

Erin Dilger^1^, Graziella Borges-Alves^2^, Vicky Carter^2^, M.G. Herededia^2^, C.M. Nunes^3^, L.M. Garcez^4^, Reginaldo Peçanha Brazil^5^, James Gordon C. Hamilton^6^, Orin Courtenay^1^

^1^School of Life Sciences, University of Warwick, Coventry, CV4 7AL, UK

^2^School of Life Sciences, Keele University, Staffordshire, ST5 5BG, UK

^3^UNESP, R. Clóvis Pestana, Dona Amelia, Araçatuba, São Paulo

^4^Instituto Evandro Chagas, Av. Almirante Barroso, Belem, Pará

^5^Fundaçao Oswaldo Cruz, Instituto Oswaldo Cruz, Lab Doenças Parasitarias, Rio de Janeiro, Brazil

^6^Infectious Disease Transmission and Biology Group, Department of Biomedical and Life Sciences, Faculty of Health and Medicine, Lancaster University, Lancaster, LA1 4YG, UK

orin.courtenay@warwick.ac.uk

In Brazil, zoonotic visceral leishmaniasis (ZVL) is transmitted by the bite of *Lutzomyia longipalpis* sandflies. *Lu. longipalpis*, are catholic in their biting habits and readily take bloodmeals from a wide range of hosts on which they demonstrate complex mating aggregation (lekking) dynamics. This results in non-linearities in the host-vector interactions that influences ZVL transmission. A crucial component of vector control is to reduce contact between vectors and infectious or susceptible hosts. Here, we report on a series of investigations to better understand these dynamics and to identify possible “push/pull” manipulation strategies towards reducing transmission. Preference of sand flies for common peridomestic host types (dogs, humans and chickens) was investigated in households under two separate scenarios (i) over a range of experimentally manipulated maintenance host (chicken) densities, to examine zoopotentiation, and (ii) in households with and without deltamethrin collars (Scalibor^®^) on dogs, to explore the effects of individual based insecticidal interventions on sand fly abundance and displacement. The results indicate significant shifts in sand fly host preference with changes in the density of the maintenance host, whereby the number of flies caught in association with dogs and people reduce as chicken numbers increase, and vice versa. This is likely related to changes in host odour biomass in conjunction with pheromone mediated aggregation behaviour. Similarly, deltamethrin dog collars also appear to confer a protective effect to households beyond individual dog protection, as collar use is related to a reduced absolute number of flies found in association with both dogs and inside houses relative to control households. The implications of these findings in relation to transmission are discussed in the wider context of host demography and vector control options by illustrating potential benefits beyond just individual protection.

**KalaCORE research on the efficacy of control measures against *Phlebotomus orientalis*, the principal vector of Visceral Leishmaniasis in East Africa**

Dia-Eldin Elnaiem^1^, Omran F. Osman^2^, Wossenseged Lemma^3^, Hanan A.A. Elhadi^4^, Bakri Y.M. Nour^5^, Noteila M. Khalid^6^, Mulat Yimer^7^, Jorgi Alvar^8^, Orin Courtenay^9^

^1^Department of Natural Sciences, University of Maryland Eastern Shore, USA

^2^Department of Zoology, Faculty of Science University of Khartoum, Sudan

^3^College of Medicine and Health Sciences, University of Gondar, Gondar, Ethiopia

^4^Department of Parasitology & Microbiology, Faculty of Medicine, Gedarif University, Gedarif, Sudan

^5^Faculty of Medicine, Blue Nile Research and Training Institute, University of Gezira, Sudan

^6^Department of Zoology, Khartoum College of Medical Science, PO Box 10995, Khartoum, Sudan

^7^College of Medicine and Health Sciences, Bahir Dar University, Ethiopia

^8^DNDI, Geneva, Switzerland

^9^School of Life Sciences, University of Warwick, Gibbet Hill Road, Coventry CV4 7AL, UK

daelnaiem@umes.edu

*Phlebotomus orientalis* is the principal vector of visceral leishmaniasis (VL) throughout East Africa. Due to poor knowledge of its behavior and bionomics and the limited attempts to evaluate different control tools, little success has been achieved in controlling this important vector of VL. KalaCORE is a DFID funded consortium initiative to support endemic countries in achieving elimination (South Asia) or improving control of visceral leishmaniasis (East Africa). The aims of KalaCORE entomological research in East Africa is to evaluate cost-effective measures that can be used for control of *Ph. orientalis* and suppression of transmission of VL in Sudan, the Republic of South Sudan and Ethiopia. This includes a sound understanding of the vector bionomics, testing the efficacy of old and new vector control tools against *Ph. orientalis* and evaluation of the acceptability and compliance in use of different control measures under different socioeconomic contexts. The project is conducted in two main study areas; one among resident population in 6 VL endemic villages in Gedaref State, eastern Sudan and the other in agricultural farms in NW Ethiopia where thousands of seasonal and migrant populations are believed to contract the disease. In this presentation we will display the plan of the study in each study area and discuss the research findings from the first year of the project.

**Visceral Leishmaniasis on the Indian Subcontinent: modelling the dynamic relationship between vector control schemes and vector life cycles**

David M. Poché^1^, William E. Grant^2^, Hsiao-Hsuan Wang^2^

^1^Genesis Laboratories, Inc. Wellington, Colorado, USA

^2^Department of Wildlife and Fisheries Sciences, Texas A&M University, College Station, Texas, USA

davidp@genesislabs.com

Visceral leishmaniasis (VL) is a disease, caused by two known vector-borne parasite species (*Leishmania donovani* and *L. infantum*) that are transmitted to man by phlebotomine sand flies (*Phlebotomus* and *Lutzomyia* spp*.*). VL results in approximately 50,000 human fatalities annually of which ≈67% occur on the Indian subcontinent. Indoor residual spraying is the current method of sand fly control in India, but given that recent research suggests *Phlebotomus argentipes* is more exophilic than once believed, alternative means of vector control, such as the treatment of livestock with systemic insecticide-based drugs, are being evaluated. We describe an individual-based, stochastic, life-stage-structured model that represents a temperature-driven sand fly vector population within a village in India and simulates the effects of vector control via fipronil-based drugs orally administered to cattle, which targets both blood-feeding adults and larvae that feed on host feces. Simulation results indicated that the efficacy of fipronil-based control schemes in reducing sand fly abundance depended on the timing of drug applications relative to seasonality of the sand fly life cycle. Taking into account cost-effectiveness and logistical feasibility, two of the most efficacious treatment schemes reduced population peaks occurring from April through August by ≈90% (applications 3 times per year at 2-month intervals initiated in March) and >95% (applications 6 times per year at 2-month intervals initiated in January) relative to no control. The cumulative number of sand fly days occurring during the peak clinical VL period (April–August) reduced by ≈83% and ≈97%, respectively, and during the summer months of peak human exposure (June–August) by ≈85% and ≈97%, respectively. Future research involving an extensive oviposition site survey and fipronil field trial would best validate the simulation results. Although this model represents a sand fly population in India exclusively, parameter values representing oviposition site and blood meal preference and daily air and soil temperatures can be easily adjusted, suggesting that our model could prove useful in *a priori* evaluation of the efficacy of fipronil-based drugs in controlling leishmaniasis on the Indian subcontinent and beyond

## Epidemiology and control (posters)

**Dynamics of *Laroussius* populations and *Leishmania* infection rate of female sand flies in an endemic visceral leishmaniasis region, Tunisia, North Africa**

Meriem Benabid, Adel Rhim, Rania Ben Romdhane, Manel Zerzri, Aïda Bouratbine

Laboratoire de Parasitologie Médicale Biotechnologie et Biomolécules LR11IPT06, Institut Pasteur de Tunis, Tunisia

meriem_benabid@yahoo.fr

In Tunisia, North Africa, visceral leishmaniasis (VL) remains primarily a pediatric disease responsible for considerable infantile morbidity and mortality. It is endemic in many foci and is mainly found in the northern and central areas of the country where the climate is favorable for the development of sand flies species of the subgenus *Larroussius*. Our interest was to study the dynamics of the *Larroussius* populations and the infection rate of female sand flies in a VL “hot spot”, where there is a VL incidence rate higher than the global average. An entomological survey was carried out monthly for one year (from June 2010 to May 2011) in two VL foci located in north eastern Tunisia: Hammed (35°59′51.13″ N/9°58′14.73″ E) and Khadhra (36°11′09.84″ N/10°02′58.39″ E) both of which are reported as belonging to the Tunisian VL “hot spot” region. Sand fly collections were done with light traps placed indoors and in animal sheds. Phlebotominae male (M) and female (F) specimens were separated by microscopy. Male specimens were identified at genus level according to morphological characters. Female specimens caught during early September were conserved separately for molecular analysis. The whole body of female sand flies was used for DNA extraction. DNA samples were analyzed for *Leishmania* infection by kinetoplast DNA (kDNA) real-time PCR. *Leishmania* species were identified by high-resolution melting (HRM) analysis of the 7SL RNA gene. Sand fly species were characterized by cycle sequencing of mitochondrial DNA fragment (cytochrome *b*). A total of 4,021 specimens were collected in the two sites (2,884 in Khadhra and 1,137 in Hammad). Sex-ratio of the combined sites was 1.2 (1,425 M/1,459 F in Khadhra plus 775 M/362 F in Hammad). The proportion of male specimens belonging to the *Larroussius* species group was higher in Khadhra than Hammad (59.1% *versus* 40.7%, *P* < 0.0001). *Phlebotomus perniciosus* was the predominant species in the 2 sites (92% in Khadhra *and* 85.9% in Hammad, *P = ns*). The number of male *Larroussius* specimens captured varied according to period of capture and showed 2 peaks: a small one in July (Khadhra) or in June (Hammad) and a more significant peak and spread over a longer period of time during mid-August–October (Khadhra) and mid-July–November (Hammad). Sixty three female specimens from the 2 sites were tested for *Leishmania* infection. Sixteen (25.4%) were positive by kDNA qPCR. Primilinary results on 7 specimens identified *Leishmania* species as *L. infantum* and sand fly species as *Ph. perniciosus*. This study showed an extended transmission season and a high *L. infantum* infection rate in *Ph. perniciosus* females in the hot spot VL region. These environmental characteristics may explain the high endemicity of visceral leishmaniasis.

**Epidemiologic survey of phlebotomine vectors in a canine leishmaniasis endemic area in Spain**

Rita Velez^1,2^, C. Ballart^1,2^, E. Domenech^3^, J. Cairó^3^, Montserrat Portús^2^, Montserrat Gállego^1,2^

^1^ISGlobal, Barcelona Ctr. Int. Health Res. (CRESIB), Hospital Clínic, Universitat de Barcelona, Barcelona, Spain

^2^Laboratory of Parasitology, Faculty of Pharmacy, Universitat de Barcelona, Barcelona, Spain

^3^Canis Veterinary Hospital, Girona, Spain

rita.velez@isglobal.org

Leishmaniasis caused by *Leishmania infantum* is an endemic vector-borne zoonosis in the Mediterranean region. Canine leishmaniasis (CanL) has a major impact in domestic dog populations, and it represents an important source of the parasite for human infection. In this region, control of both human and canine leishmaniasis greatly depends on an effort to reduce infection burdens in the animal host and phlebotomine vector surveillance. Recently, a vaccine to prevent CanL has been released in Europe (France, Italy, Portugal and Spain) and a project to evaluate its effectiveness in an endemic area of northern Spain has being underway since mid-2015. Though it is known that *Phlebotomus perniciosus* and *Ph. ariasi* are the vectors in *L. infantum* transmission in Spain, little is known about their current distribution or the presence of other sand fly species in Northern Spain. Therefore, a preliminary phlebotomine epidemiologic survey was conducted simultaneously with a CanL seroprevalence study in order to characterize the area where the vaccine field study would take place. A total of 20 dog kennels located in Girona province (Catalonia, northern Spain) were sampled during September 2015. Dog densities in these kennels ranged from 4 to 34 animals, with an average of 16 dogs per sampling site. These were mainly located in rural areas at altitudes varying from 70 m to 400 m above sea level. One to two CDC light traps were placed for one night in each sampling station, from which 33 traps were recovered. All sand flies were preserved in 70% ethanol until mounting in Hoyer’s medium for morphological identification under optical microscopy. A total of 133 phlebotomine specimens were recovered, from which 60 were females (45%). Sand flies were trapped in 13 sampling stations and the species identified in order of decreasing abundance were *Ph. perniciosus* (53%), *Ph. ariasi* (32%) and *Sergentomyia minuta* (15%). *Ph. perniciosus* was present in 50% of the sampling locations (frequency), ranging from altitudes of 84 m to 343 m a.s.l. The frequency of *Ph. ariasi* was 30% and it was found at altitude ranges of 139 m to 259 m a.s.l. *Se. minuta* was found between 73 m and 285 m a.s.l. and presented a frequency of 45%. These preliminary results allow an insight into the phlebotomine vectors present in an endemic CanL region and support the selection of this location for carrying out a CanL vaccine trial. A further characterization of these arthropod populations combined with results from currently ongoing CanL seroprevalence studies will enable a better understanding of this zoonosis and the identification of transmission risk factors in this geographic area.

Financial support: European Union’s Horizon 2020 research and innovation programme under the Marie Sklodowska-Curie grant agreement N° 642609.

**Evidence for stable endemic sand fly populations in the light of migration streams into Austria**

Adelheid G. Obwaller^1,4^, Mehmet Karakus^2^, Wolfgang Poeppl^3^, Seray Toz^3^, Yusuf Ozbel^3^, Horst Aspöck^4^, Julia Walochnik^4^

^1^Federal Ministry of Defence and Sports, Division of Science, Research and Development, Vienna, Austria

^2^Ege University, Faculty of Medicine, Parasitology Department, Bornova, Izmir, Turkey

^3^Department of Dermatology, Medical University of Vienna, Austria

^4^Institute of Specific Prophylaxis and Tropical Medicine, Center for Pathophysiology, Infectiology and Immunology Medical University Vienna, Austria

julia.walochnik@meduniwien.ac.at

Sand flies (Diptera: Psychodidae: Phlebotominae) are the vectors of various pathogens of medical-veterinary importance, including *Leishmania* spp., *Bartonella* spp. and Phleboviruses. In Central Europe, leishmaniasis is a rare disease diagnosed almost exclusively in travellers, soldiers or migrants coming from tropical or subtropical countries. Leishmaniases are however a major public health problem in the Eastern Mediterranean region and the Middle East, reinforced by war, which has resulted in a massive stream of refugees into Europe. Sand fly trapping was performed at different capture sites in South-Eastern Austria in July and August in the years 2012, 2013 and 2015. In several regions of Austria sand fly populations were shown to be stable if not increasing. All individuals found were identified as *Phlebotomus* (*Transphlebotomus*) *mascittii* Grassi 1908, of which one unfed female individual was infected with *L. infantum*. A possible circulation of *Leishmania* spp. might become an important issue in Central Europe.

**Absence of *Leishmania*-infected phlebotomines in gallery forests of the Federal District of Brazil**

Aline Machado Rapello^1^, Thaís Tâmara Castro Minuzzi-Sousa^1^, Tamires Emanuele Vital^2^, Tauana Ferreira^1^, Renata Velôzo Timbó^1^, Andrey José de Andrade^1,3^, Rodrigo Gurgel Gonçalves^1^

^1^Laboratório de Parasitologia Médica e Biologia de Vetores, Área de Patologia, Faculdade de Medicina, Universidade de Brasília, Brasil

^2^Laboratório Interdisciplinar de Biociências, Área de Patologia da Faculdade de Medicina, Universidade de Brasília, Brasil

^3^Laboratório de Parasitologia Molecular, Departamento de Patologia Básica, Universidade Federal do Paraná, Brasil

aline_rapello@hotmail.com

The aim of this study was to detect *Leishmania*-infected phlebotomines in gallery forests of Brasília, Federal District of Brazil (FD), during the dry and the rainy seasons of 2014. Sand flies were captured in four areas, Água Limpa Farm (FAL), Biological Reserve of Contagem (REBIO), Brasilia’s National Park (PNB) and Botanic Garden of Brasília (JBB), in May and September, 2014. The entire capture effort entailed 1,280 HP light traps and 16 Shannon traps. A total of 1,209 sand flies were captured and dissected. Their heads and genitalia were cleared and mounted in Canada balsam for species identification. Other female body parts (thorax, part of the abdomen, legs, and wings) were placed in microtubes with PBS 1× (one specimen for each labeled tube) and frozen. A variable number of female specimens (one to ten), belonging to the same species and capture sites, were pooled for total DNA extraction using the Illustra tissue and cells genomicPrep Mini Spin Kit. Integrity of the samples was checked by a PCR designed to amplify the cacophony gene IVS6 region in sand flies. Trypanosomatid detection was performed by amplifying the SSU rDNA and ITS-1 regions. Male phlebotomines (614) from 14 species and females (594) from 13 species were identified. These included *Bichromomyia flaviscutellata* and *Nyssomyia whitmani*, which are considered to be potential vectors of *Leishmania amazonensis* and *L. braziliensis*, respectively. DNA was extracted from 569 females grouped in 87 pools. Fragments that corresponded to the cacophony gene were amplified in all of the samples, demonstrating the integrity of the extracted DNA. All samples tested negative for *Leishmania* spp. Despite negative results, the high population density of potential vector species in the studied areas, the presence of *Leishmania*-infected small mammals in gallery forests of FD and the known adaptation of *Ny. whitmani* in anthropic environments are risk factors for *Leishmania* transmission in the area. Positive canine and human cases of leishmaniases in adjacent areas of the studied gallery forests were registered, therefore negative results in the present study do not implicate the absence of infected vectors in these gallery forests and surroundings.

**Vectors of the subgenus *Leishmania* (*Viannia*) in the Tapajós national forest reserve located in the lower Amazon Region of Brazil**

Adelson Alcimar de Souza^1,†^, Thiago Vasconcelos dos Santos^1^, Yara Lins Jennings^1^, Edna Aoba Ishikawa^2^, Iorlando Barata^1^, Maria das Graças Silva^1,†^, José Aprígio Lima^1^, Jeffrey Shaw^3^, Ralph Lainson^1,†^, Fernando Silveira^1^

^1^Parasitology Department, Evandro Chagas Institute, Belém, Pará State, Brazil

^2^Tropical Medicine Nucleus, Federal University of Pará, Belém, Pará State, Brazil

^3^Biomedical Sciences Institute, São Paulo University, São Paulo State, Brazil

^†^In memoriam

jayusp@hotmail.com

In the Brazilian Amazon region American cutaneous leishmaniasis (ACL) is caused by at least seven *Leishmania* species *Leishmania* (*Viannia*) *braziliensis*, *L.* (*V.*) *guyanensis*, *L.* (*V.*) *lainsoni*, *L.* (*V.*) *shawi*, *L.* (*V.*) *naiffi*, *L.* (*V.*) *lindenbergi* and *L*. (*Leishmania*) *amazonensis.* The distribution of these species often overlaps within a habitat and they are associated with complex transmission cycles that involve different vectors. The national forest reserves, known locally as FLONAs (FLOresta NAcional) are preserved by law. There are 65 registered FLONAs in Brazil and 13 are located in the Pará State that includes the Tapajós FLONA. These reserves offer unprecedented opportunities to study the natural transmission cycles of the different *Leishmania* species which in this area of Brazil are dominated by *L.* (*Viannia*) species. In total 9,704 sand flies (6,179 females/3,525 males) were captured over a 2 year period between May 2003 and October 2004 using CDC lights traps set at 1.5 m and 20 m (above ground level), a Shannon trap and aspirations from tree bases. Female flies were dissected, identified and cultures made of any flagellates found in their intestines. Isolates were identified by MLEE and monoclonal antibodies. Species abundance was expressed for the four surveyed ecotopes (ground, canopy, Shannon and tree base collections) with index of species abundance (ISA) and standard index of species abundance (SISA). The top 10 SISA indices were as follows: 1. *Nysommyia umbratilis*; 2. *Ny. whitmani*; 3. *Trichophoromyia ubiquitalis*; 4. *Psychodopygus complexus/Ps.wellcomei*; 5. *Ps. davisi*; 6. *Ny. shawi*; 7. *Micropygomyia rorataensis*; 8. *Vianamyia furcata*; 9. *Ny. anduzei*; 10. *Ps. paraensis.* Of these 10 species, *Viannia* infections have previously been found in nine and in our study we identified *Viannia* infections in species whose SISA ranking was 2 (*Ny. whitmani*), 6 (*Ps. davisi*), 11 (*Lu. gomezi*) and 13 (*Ps. h. hirsutus*). Natural flagellate infections were found in 18 of 6,179 dissected females (infection rate: 0.29%) of the following 8 species: *Ny. whitmani* [SISA index 2] (6/486, 3 from CDC 1.5 m, 1 from CDC 20 m and 2 from Shannon), *Mi. pilosa* [SISA index 32] (4/19, 3 from CDC 1.5 m and 1 from CDC 20 m), *Ps. davisi* [SISA index 6] (2/388, from Shannon), *Sc. sordellii* [SISA index 29] (2/35, from CDC 1.5 m), *Lu. gomezi* [SISA index 11] (1/135, from Shannon), *Ps. h. hirsutus* [SISA index 13] (1/100, from CDC 20 m), *Evandromyia infraspinosa* [SISA index 32] (1/76, from CDC 1.5 m) and *Ny. shawi* [SISA index 6] (1/280, from CDC 1.5 m). Of the 18 infections 8 were successfully cultured and 7 were *Leishmana*. Of the 7 infections that were successfully identified L*.* (*V.*) *s. shawi* was found in *Ny. whitmani* (3) and *Lu. gomezi* (1) and *L.* (*V.*) *naiffi* in *Ps. davisi* (2) and *Ps. h. hirsutus* (1). Our results support the hypothesis that *L.* (*Viannia*) species may have more than one vector and that *Ps. davisi* and *Ps. h. hirsutus* are important vectors of *L.* (*V.*) *naiffi.* The finding of a *L.* (*V.*) *s. shawi* in a *Lutzomyia*, *Lu. gomezi*, raises the question of its potential transmission by sand flies belonging to the other genera. The mere presence of 10 sand fly species previously linked to the transmission as well as others that have been found infected elsewhere indicates how very complex cutaneous leishmaniasis transmission is in the Tapajós FLONA. Based on our past experience, 6 infections in *Mi. pilosa* and *Ev. infraspinosa* most probably belonged to a lizard or anuran trypanosome. SISA values need to be interpreted with caution as to their importance as indicating vectors. For instance the high SISA ranking of *Mi. rorataensis* was due to a large number of males that were captured from the base of a tree trunk were it was the dominant species and feeds preferentially on cold blooded vertebrates. With this in mind we consider that SISA indices are important in evaluating vector potential and that our results support this view.

**Natural transovarial and transstadial transmission of *Leishmania infantum* in *Rhipicephalus sanguineus* (Acari: Ixodidae)**

Kourosh Azizi^1^, Qasem Asgari^2^, Mohammad Djaefar Moemenbellah-Fard^1^, Aboozar Soltani^1^, Tahereh Dabaghmanesh^3^

^1^Research Centre for Health Sciences, Department of Medical Entomology and Vector Control, School of Health, Shiraz University of Medical Sciences, Shiraz, Iran

^2^Basic Sciences in Infectious Diseases Research Center, Department of Parasitology and Mycology, School of Medicine, Shiraz University of Medical Sciences, Shiraz, Iran

^3^Department of Medical Entomology and Vector Control, School of Health, Shiraz University of Medical Sciences, Shiraz, Iran

dabaghmanesh@gmail.com

The visceral leishmaniasis parasite, *Leishmania infantum*, is naturally transmitted through the bites of phlebotomine sand flies. Alternative routes of transmission have been suggested. The main aim of this study was to verify the passage of *L. infantum* kDNA in ticks, *Rhipicephalus sanguineus*, blood feeding on a parasitemic dog in Shiraz, south of Iran. Overall, 180 *Leishmania*-free ticks were collected from the field and then fed on lab rodents. They were then were divided into eight groups and allowed to blood-feed on a dog, *Canis familiaris*, for fixed periods of time. These and all third-generation stages of ticks were checked for *L. infantum* kDNA using conventional PCR protocol at time intervals. The infection rate was significantly higher in female than male ticks (*P* = 0.043). The rates were higher among nymphs (50%) than adult ticks (41.7%). The kDNA of *L. infantum* was not detected in ticks 24 h post-feeding. It was, however, positive among the second to fourth groups of nymphs (40, 50 and 55%) and adult (40, 46.7 and 36.7%) ticks. Eggs and unfed larvae recovered from the third and fourth adult groups (2w, 4w) were 100% PCR-positive. The data revealed the passage of *L. infantum* kDNA in nymphs and adults of brown dog tick following fixed time intervals post blood feeding on an infected dog. The natural transovarial and transstadial passage of kDNA through ticks was shown.

**Molecular epidemiology of phlebovirus in four provinces in Morocco**

Nargys Es-Sette^1^, Malika Ajaoud^1^, Rémi N. Charrel^2^, Meryem Lemrani^1^

^1^Laboratoire de Parasitologie et de Maladies Vectorielles, Institut Pasteur du Maroc, Casablanca, Morocco

^2^UMR EPV “Émergence des Pathologies Virales”, Aix Marseille Université, IRD U190, INSERM U1207, IRBA, EFS, EHESP; Marseille, France & Fondation Méditerranée Infection, APHM Public Hospitals of Marseille, Marseille, France

meryem.lemrani@pasteur.ma

Sand flies are vectors of protozoa, viruses, and bacteria. To investigate the transmission of phleboviruses, a total of 8,753 sandflies were collected in four foci of leishmaniasis. A total of 16 species were morphologically identified. Cell culture and Nested-PCR screening for phleboviruses, using an assay targeting the polymerase gene, showed positive results for 19 pools of sand flies belonging to different species that had originated in four different foci, and were different from those commonly reported in the literature. Sequencing of the corresponding products confirmed these results and allowed identification of Toscana virus exclusively. Sequence analysis shows that Moroccan Toscana virus belonging to genotype B and appear close to Toscana virus isolated in France and Spain. This study reported the existence of the virus in the north, center and the south of the country. The abundance and diversity of sand flies in Morocco and Mediterranean climate, would support the continuous circulation of Toscana virus in our country, posing a potential risk of emergence of this arbovirus.

**Phleboviruses circulating in sand flies in Emilia-Romagna region (Northern Italy) in 2013–2015**

Mattia Calzolari^1^, Romeo Bellini^2^, Paolo Bonilauri^1^, Marco Pinna^1^, Francesco Defilippo^1^, Michele Dottori^1^, Paola Angelini^3^

^1^Istituto Zooprofilattico Sperimentale Della Lombardia e dell’Emilia Romagna “B. Ubertini” (IZSLER), Reggio Emilia, Italy

^2^Centro Agricoltura Ambiente “G. Nicoli”, Crevalcore, Italy

^3^Regione Emilia-Romagna, Bologna, Italy

mattia.calzolari@izsler.it

Phlebotomine sand flies are the biological vectors of a variety of viruses belonging to the Phlebovirus genus. As several of these viruses, like Toscana virus, are important agents of diseases in humans, defining the phleboviruses circulating in a particular area is an important health issue. To monitor the presence of phleboviruses, a surveillance system, based on sampling and testing of sand flies, was activated in the Emilia-Romagna region (Northern Italy). The system detected the co-circulation of three different phlebovirus: the well-known Toscana virus and other two previously unreported phleboviruses, tentatively named Ponticelli virus and Sole virus. Sandflies were sampled in 151 geo-referenced sites by suction traps, baited with carbon dioxide, and activated overnight. Sites were placed mainly in the hilly areas of the Region, which are characterized by ecological conditions particularly favorable to sand-flies. Between 2013 and 2015 a total of 90,506 sandflies were sampled. A subsample of 2,527 specimens were morphologically identified: 2,441 (96.5%) were *Phlebotomus perfiliewi* and 86 (3.5%) were *Ph. perniciosus*. This result is consistent with previous results obtained in Emilia-Romagna, which show the overwhelming presence of the *Ph. perfiliewi* compared to *Ph. perniciosus*. The largest number of sand flies (82,181) was collected in 2013. In this year we also caught the largest number of specimens per trap per night, with more than 10,000 sand flies in two different sites. A total of 87,492 sandflies, sorted in 321 pools, were submitted to a Real Time PCR analysis that targeted the Toscana virus, and 35 of these pools, from 17 sites, tested positive. Moreover 26,853 sandflies (in 110 pools) were tested with a pan-phlebovirus PCR followed by the sequencing of produced amplicons, giving 52 positive pools. The phylogenetic analysis made with the homologous sequences of other phleboviruses available in Genbank, suggest the presence of two previously unreported phleboviruses, highlighted by the presence of two well supported clades in the resultant tree. One of these clades falls with the Salehabad serocomplex, and the respective virus has been tentatively named Ponticelli virus, the other clade falls in the Sand fly fever Naples serocomplex, and the respective virus has been tentatively named Sole virus. Both viruses were detected in all the three years of survey in different locations, Ponticelli virus in 11 sites and Sole virus in 10 sites. Interestingly the sequence ascribed to Sole virus was also detected in sand flies from the neighboring Lombardia Region. The isolation and detection of non-described phlebovirus is consistent with the wide variety of new phleboviruses reported in last ten years, especially in Mediterranean basin. Despite Toscana virus having been described as the major cause of summer meningitis in Italy, France and Spain, this virus remains a neglected pathogen. Moreover the discovering of new phleboviruses, reported in this study, and in the Mediterranean basin, raises the issue of their infectious potential, since several of these viruses are serologically detected in vertebrates and show the ability to grow on Vero cells. The sympatric co-circulation of different phlebovirus reported in this study, indicate a very dynamic and complex situation, which deserves a more detailed investigation to characterize the circulation and the possible pathogenicity to humans and animals of these uncharacterized viruses.

**Isolation of Piura virus, an insect-specific negevirus, from *Lutzomyia evansi* in Colombia**

María Angélica Contreras-Gutiérrez^1,2^, Hilda Guzman^3^, Marcio R.T. Nunes^4^, Sandra Uribe^2^, Rafael Vivero^1,2^, Iván Darío Vélez^1^, Nikos Vasilaskis^3^, Robert B. Tesh^3^

^1^Programa de Estudio y Control de Enfermedades Tropicales – PECET – SIU-Sede de Investigación Universitaria – Universidad de Antioquia, Medellín, Colombia

^2^Grupo de Investigación en Sistemática Molecular-GSM, Facultad de Ciencias, Universidad Nacional de Colombia, sede Medellín, Medellín, Colombia

^3^Department of Pathology, University of Texas Medical Branch at Galveston, TX, US

^4^Center for Technological Innovation, Evandro Chagas Institute, Ministry of Health, Ananindeua, Pará, Brazil

maria.contreras@pecet-colombia.org

Phlebotomine sand flies are known vectors of protozoa (*Leishmania* spp.), bacteria (*Bartonella bacilliformis*), and a diverse group of viruses of public health and veterinary importance, including members of the families *Rhabdoviridae*, *Bunyaviridae*, and *Reoviridae*. However, the increasing interest in and discovery of, the diverse nature of the viral microbiome in insects, indicates that phlebotomine sand flies are also naturally infected with other types of insect-specific viruses. These viruses are widely distributed in Diptera around the world. Here we report a new strain of Piura virus (CoR 10), isolated in C6/36 mosquito cells from a pool of adult *Lutzomyia evansi* collected in 2013 from Sucre Department on the Caribbean coast of Colombia. Piura virus induced a rapid cytopathic effect in a C6/36 cell culture. Genome sequencing of CoR 10 showed a genomic RNA of 10500 Nt with poly-A tail positive strand, and polycistronic encoding the viral mRNA in 3 ORFs. Nucleotide and amino acid sequence comparison with sequences of other Negevirus available on Genbank showed that there was 99% identity between CoR 10 and the prototype PIUV, meanwhile, a low nucleotide and amino acid identity was obtained (60–70%) with other members of the Negevirus taxon. According to phylogenetic analysis, CoR 10 is member of the genus *Nelorpivirus*, negevirus taxon, and is a new strain of the recently described PIUV Clade, which includes isolates from Peru, Mexico, and now Colombia. This is the first report of an insect-specific virus from Colombia, and also, the first register of PIUV isolated from phlebotomine sand flies.

**Characterization of susceptibility of Phlebotominae (Diptera: Psychodidae) to the insecticide, alpha-cypermethrin**

Douglas de Almeida Rocha^1^, Andrey José de Andrade^2,4^, Luciana Moura Reinaldo^3^, Marcos Takashi Obara^1^

^1^Núcleo de Medicina Tropical, Universidade de Brasília, Brasil

^2^Laboratório de Parasitologia Médica e Biologia de Vetores, Faculdade de Medicina, Universidade de Brasília, Brasil

^3^Departamento de Estatística – Instituto de Ciências Exatas, Universidade de Brasília, Brasil

^4^Departamento de Patologia Básica, Setor de Ciências Biológicas, Universidade Federal do Paraná, Brasil

dougalmeidarocha@gmail.com

Leishmaniases are a group of infectious diseases primarily transmitted by infected female phlebotomine sand flies. Chemical insecticides used against vector species are one of the control measures for these diseases. In Brazil the pyrethroid alpha-cypermethrin has been recognized by the Ministry of Health. Despite the continuous and intensive control campaigns few studies have been carried out to detect changes in the susceptibility of sand flies to insecticides. The objective of this study was characterize the susceptibility profile of sand fly populations to the pyrethroid alpha-cypermethrin. Sand flies caught in the field in six Brazilian municipalities and specimens from a laboratory colony classified as the Susceptibility Reference Lineage (SRL) were evaluated using CDC bottles with different concentrations of alpha-cypermethrin (3 μg/mL, 5 μg/mL, 7 μg/mL and 9 μg/mL). An acetone control was used for comparison. Males and females from each municipality were tested in the bottle bioassay (number of replicates = 3). A total of 2,198 sand fly specimens were captured and *Lutzomyia longipalpis* was the species most frequently found in all municipalities. It was observed that CDC bottles can be used to evaluate the susceptibility of sand flies to insecticides and in the present study we estimated that the discriminating dose to the SRL population was 2.38 μg/mL. Kaplan-Meier survival curves showed that specimens of Montes Claros and Paracatu municipalities were tolerant to alpha-cypermethrin, compared to specimens from Pirenópolis, Unaí, Januária, and Belo Horizonte. The time taken to kill 50% of sand flies varied according to the insecticide concentrations that they were exposed to: 40 min for 9 ug/mL, 50 min. for 7 ug/mL, 60 min. for 5 ug/mL and 70 min. for 3 ug/mL. All sand fly populations, including the specimens characterized as SRL, showed changes in susceptibility profiles, indicating tolerance to alpha-cypermethrin.

Financial support: CAPES.

**Evaluation of the level of knowledge of public health professionals regarding the vector of visceral leishmaniasis and its control measures**

Anna Ariel Polegato Martins^1^, Mariana Fuga^1^, Alessandra Gutierrez de Oliveira^2^, Mirella Ferreira da Cunha Santos^1^

^1^State University of Mato Grosso do Sul (UEMS), School of Medicine, Campo Grande, Mato Grosso do Sul, Brazil

^2^Federal University of Mato Grosso do Sul (UFMS), Parasitology laboratory, Campo Grande, Mato Grosso do Sul, Brazil

mirella.santos@uems.br

The sand fly *Lutzomyia longipalpis* is the major vector of *Leishmania* (*Leishmania*) *infantum*, the causative agent of visceral leishmaniasis in Central and South America. A thorough understanding of the transmission mechanism of any infectious agent is crucial to implementing an effective intervention strategy. An accurate identification of sand fly species and of the epidemiological aspects concerning leishmania vectors is therefore important, especially to public health professionals in endemic areas. Based on theoretical public opinion research reference methodology, this study investigated the level of knowledge of the vector of visceral leishmaniasis (VL) by social actors directly involved in the prevention and control of the disease. A total of 67 public health professionals, in 10 Basic Units of Family Health (UBSF) in Campo Grande, Mato Grosso do Sul, Brazil, answered the questionnaire containing 11 questions regarding sand flies, VL and control measures. Almost 97% of those who answered the questionnaire considered VL relevant to public health, but only 80% declared knowing the main symptoms of the disease. Some 88% referred to sand flies as responsible for the transmission of VL, but were not capable of describing morphological features. 77% knew of some measures of insect control and 67% had given some guidance on vector control to the public. The directions that they reported that they had given to the population were wrong in 55% of cases. These results demonstrate weakness between the theoretical and practical understanding, since most professionals were not able to offer correct instructions on leishmaniasis and its vectors, although they considered that they had sufficient knowledge of the issue.

**Comparison of various recombinant salivary proteins as epidemiological markers for dog exposure to *Phlebotomus perniciosus* in different localities in Italy, Portugal and Spain**

Laura Willen^1^, Tatiana Kostalova^1^, Nikola Polanska^1^, Tereza Lestinova^1^, Carla Maia^2,3^, Petra Sumova^1^, Michaela Vlkova^1^, Eleonora Fiorentino^4^, Aldo Scalone^4^, Gaetano Oliva^5^, Fabrizia Veronesi^6^, José Manuel Cristóvão^2^, Orin Courtenay^7^, Lenea Campino^2,8^, Luigi Gradoni^4^, Marina Gramiccia^4^, Cristina Ballart^9,10^, Montserrat Gállego^9,10^, Petr Volf^1^

^1^Dept. Parasitol., Charles University, Prague, Czech Republic

^2^Med. Parasitol. Unit, GHTM, IHMT, Universidade Nova de Lisboa, Portugal

^3^Fac. Med. Vet., Universidade Lusófona de Humanidades e Tecnologias, Lisboa, Portugal

^4^Istituto Superiore di Sanità, Rome, Italy

^5^Dept. Vet. Med. Animal Production, University Federico II, Naples, Italy

^6^Dept. Vet. Med., Universityof Perugia, Italy

^7^WIDER and School of Life Sci., University of Warwick, UK

^8^Dept. de Ciências Biomédicas e Medicina, Universidade do Algarve, Faro, Portugal

^9^Lab. Parasitol., Universitat de Barcelona, Spain

^10^ISGlobal, Hospital Clínic – UB, Barcelona, Spain

laura.willen@gmail.com

In Europe *Phlebotomus perniciosus* (Diptera: Psychodidae) is known as the main vector of *Leishmania infantum* (Trypanosomatida: Trypanosomatidae), the parasite that causes canine and human leishmaniasis. When hosts are bitten, the injected saliva of the sand fly will elicit an antibody response in the host, which enables the measurement of the frequency of sand fly – host contact. Since the use of whole salivary gland homogenate (SGH) is a time-consuming and labour-intensive process, recombinant salivary proteins and salivary peptides are being suggested as a valid replacement for SGH in large-scale serological studies. Moreover, the use of peptides as epidemiological markers in large-scale serology studies is beneficial because of the opportunity to decrease the chance of cross-reactivity, and thus increasing the specificity of the antibody response against antigens of *Ph. perniciosus*. However, it is essential to determine if these antigens display the same sensitivity in the different localities where *Ph. perniciosus* is present. In this study, 214 sera samples from naturally exposed dogs from Campania (south Italy) and Umbria (central Italy), 341 sera samples from Metropolitan Lisbon region (Portugal), and a total of 60 sera samples from Catalonia and Balearic Islands (Spain) were tested with *Ph. perniciosus* 43 kDa yellow-related recombinant protein (rSP03B), 42 kDa yellow-related recombinant protein (rSP03) and SGH. Studies on salivary peptides that represent the most antigenic parts of the proteins are in progress. In all the sampling areas, where *Ph. perniciosus* is the principal vector of *L. infantum*, a strong correlation was observed between the antibody response against SGH and rSP03B, suggesting that different populations of *Ph. perniciosus* share similar antigenic properties of this salivary protein. Furthermore, no significant differences were detected across regions, supporting the use of rSP03B as a universal epidemiological marker throughout the geographical distribution of *Ph. perniciosus*. In contrast, correlation between antibody response against SGH and rSP03 was only found in sera from the two localities in Spain. This suggests that rSP03 is not suitable as universal marker throughout the geographical distribution of *Ph. perniciosus*. In order to confirm the presence of similar antigenic epitopes in the native yellow-related protein and rSP03B, an inhibition blot was performed. This resulted in a clear inhibition of the binding of IgG to the native protein after pre-incubating the sera with rSP03B. The same procedure was performed with rSP03; in this case no inhibition of the binding of IgG to the native protein was observed which ensures the specificity of the antibody response against rSP03B. In conclusion, we propose rSP03B as a universal marker of sand fly exposure throughout the geographical distribution of *Ph. perniciosus*.

Financial support: Partially by Charles University (GAUK – 1642314/2014), the EU grants FP6-010284 EDEN and FP7-261504 EDENext, the Spanish projects AGL2004-06909-C02-01, CGL2007-66943-C02-01/BOS and CG12010-22368-C02-01 and the European Union’s H2020 Programme under the MSCA GA n° 642609.

**Can we identify *Leishmania* super-spreaders to reduce transmission to sand fly vectors?**

Aurore Lison^1^, Steve Reed^2^, Orin Courtenay^1^

^1^School of Life Sciences, University of Warwick, CV4 7AL, Coventry, UK

^2^Infectious Disease Research Institute, 1616 Eastlake Avenue East, Seattle, Washington, USA

orin.Courtenay@warwick.ac.uk

Heterogeneities in the vector-host interactions leading to onward transmission will determine the degree of aggregation in infection, and ultimately in the effort required to reduce transmission. In longitudinal xenodiagnosis studies of natural infection with *Leishmania infantum*, a large fraction of transmission events to the sand fly vector *Lutzomyia longipalpis* is from a very small fraction of the infected canine reservoir population. Most infected individuals are not significantly infectious, and the available serological tests, such as those based on anti-*Leishmania* antigens rK39 and rK28, are designed to detect current and historical exposure, thus may fail to target the highly infectious population which is desirable to directly impact on transmission. Targeting these “super-spreaders”, and the sand flies that they infect, to manage visceral leishmaniasis incidence requires a different approach to current suboptimal blanket control strategies, as these epidemiologically significant vector-host interactions will otherwise require extremely high intervention coverage to successfully include them. Increasing the specificity is also likely to reduce the likewise non-infectious (and asymptomatic) infection dogs. The primary objective of this study was to test existing and novel anti-*Leishmania* antigens and certain combinations in order to identify and differentiate super-spreaders in the mixed reservoir population. The ELISA assays were performed on archived sera collected from a naturally infected cohort population of Brazilian dogs. Their transmission potential was measured by xenodiagnoses during a two years longitudinal study. These dog sera samples are well characterized in terms of infection, disease and infectiousness; and classified as infected, latent infected and infectious, current and in their sampled lifetime. Here we present preliminary results towards modeling the impact of such a new diagnostic test specific to reduce transmission.

**Vector control using long-lasting insecticidal nets against kala-azar in Bangladesh**

Chizu Sanjoba^1^, Yusuf Ozbel^2^, Bunpei Tojo^3^, Eisei Noiri^3^, Yoshitsugu Matsumoto^1^

^1^Laboratory of Molecular Immunology, Graduate School of Agricultural and Life Sciences, University of Tokyo, Tokyo, Japan

^2^Department of Parasitology, Ege University of Faculty of Medicine, Bornova, Izmir, Turkey

^3^Division of Advanced Medical Science of Nephrology, the University of Tokyo Hospital, Tokyo, Japan

asanjoba@mail.ecc.u-tokyo.ac.jp

Kala-azar is one of the major public health problems in Bangladesh, where the disease has been endemic for many decades. Vector control takes on a role as an important part in controlling the disease which is transmitted by phlebotomine sand flies, but the most appropriate vector control measures is still a matter of debate. Here, the efficacy of two long-lasting insecticidal nets (LLINs), Olyset^®^ and Olyset^®^ Plus against field collected sand flies was evaluated in a kala-azar endemic area in Bangladesh. Sand fly bioassays were conducted according to the WHO-approved cone test methodology with modification. The major species of sand flies (91.28%) tested was *Phlebotomus argentipes*. Sand flies were introduced into a plastic cone which had a piece of Olyset^®^ or Olyset^®^ Plus netting attached over the wide-end for 3 min and then removed and placed in a plastic cup and provided with sucrose solution soaked onto an absorbent cotton pad. Mortality was recorded 24 h after the exposure. Approximately 20–25 sand flies were used in each cone-test, which was repeated 4 times. The mortality of sand flies recorded 24 h after the exposure was 100% in the Olyset^®^ Plus group while the mortality of sand flies in the Olyset^®^ group was 83.63% (corrected mortality = (% test mortality − % control mortality)/(100 − % control mortality) × 100). It is essential to understand the knowledge, attitude and practice of people who live in an endemic area towards Kala-azar in order to propose successful vector control strategies. Therefore, a questionnaire-based survey was also carried out to understand whether or not vector control using LLINs is a sustainable application. The questionnaire consisted of three sections, socio-demographic characteristics, knowledge of kala-azar and history of kala-azar and perceptions of kala-azar vector control. Based on the analysis of 1,393 households, the knowledge, attitude and practice of the people who live in the endemic area about kala-azar was relatively low. Though utilization of LLINs is promising, its ownership is noticeably low. Vector control using Olyset^®^ Plus could be a potential tool for reducing the morbidity rate of kala-azar in an endemic area in Bangladesh but any control program would require community based education and acceptance.

Financial support: JST/JICA, Science and Technology Research Partnership for Sustainable Development.

**Analysis of gene expression in a *Lutzomyia longipalpis*-derived cell line**

Luzia M.C. Cortes^1^, Barbara C.A. Melo^1^, Franklin Souza-Silva^1^, Bernardo A.S. Pereira^1,2^, Felio J. Bello^2^, Otacilio C. Moreira^1^, Daniela de Pita-Pereira^1^, Constança Britto^1^, Carlos R. Alves^1^

^1^Laboratório de Biologia Molecular e Doenças Endêmicas-IOC, Fiocruz, Av. Brasil, 4365 Manguinhos CEP 21045-900 – Rio de Janeiro, RJ – Brasil

^2^Universidad Antonio Nariño, Facultad de Medicina, Bogotá, Colombia

lmccortes@gmail.com

Parasites of the genus *Leishmania* are transmitted to mammals during blood feeding by sand flies (Diptera: Psychodidae: Phlebotominae), which are widely distributed in tropical and subtropical regions worldwide. Preventing the development of the parasites in the gut of the vector sand fly is a feasible strategy to control the infection. The success of this strategy will only be achieved with the full knowledge of the interactive events that occur between the parasite and its vector, especially those that take place in the sand fly gut. *In vitro* studies with insect cell lines can be useful to shed light on some of these interactions, as they provide a more controlled environment. In this context, it was previously demonstrated that a cell line obtained from embryonic tissue of *Lutzomyia longipalpis* (Lulo cells) can be an appropriate model for such studies. Therefore, we aimed to develop molecular and biochemical studies of Lulo cells to further chatacterize this cell line’s features and, thus, its actual application for studies with *Leishmania* spp. Recently, in a proteomic approach to studying Lulo cells, we observed that promastigotes of *Leishmania* (*Viannia*) *braziliensis* are able to bind to some proteins of the Lulo cells (data submitted for publication). Based on this previous study, we analysed, by Real-Time PCR, the expression profile of some Lulo cell genes, which we observed to be relevant for host-parasite interactions, before and after incubation of Lulo cells with *L.* (*V.*) *braziliensis* during different periods of time. The gene targets are based on the following *Lu. longipalpis* protein sequences: enolase, zinc ion-binding protein, heat shock protein, oxidoreductase, peroxiredoxin and putative Cu/Zn superoxide dismutase. As housekeeping reference genes, we used the *Lu. longipalpis* GAPDH and RD 49 genes. The relative expression of the target genes was assessed using the ΔΔCt methodology. Trials in different times of interaction with the parasite *Leishmania* spp. will be conducted to simulate what happens in the phlebotomine gut and to evaluate the possible role of these molecules of the parasite during the process of access to vector. This study represents a step towards the establishment of a new *in vitro* model of the interaction between *Leishmania* and the Lulo cells in order to simulate the events that occur in the digestive tract of infected insects.

## Modern tools for sand flies studies (oral communications)

***Leishmania* HASP and SHERP genes are required for *in vivo* differentiation, parasite transmission and host virulence attenuation**

Johannes S.P. Doehl^1^, Jovana Sádlová^2^, Hamide Aslan^3^, Sonia Metangmo^3^, Jan Votýpka^2^, Shaden Kamhawi^3^, Petr Volf^2^, Deborah F. Smith^1^

^1^Centre for Immunology and Infection, Department of Biology, University of York, York, UK

^2^Department of Parasitology, Faculty of Science, Charles University, Prague, Czech Republic

^3^Vector Molecular Biology Section, Laboratory of Malaria and Vector Research, National Institute of Allergy and Infectious Diseases, National Institutes of Health, Rockville, Maryland, USA

johannes.doehl@gmail.com

Differentiation of extracellular *Leishmania* promastigotes within their sand fly vector, termed metacyclogenesis, is considered to be essential for parasites to regain mammalian host infectivity. Metacyclogenesis is accompanied by changes in the local parasite environment, including secretion of complex glycoconjugates (the promastigote secretory gel) and colonization and degradation of the sand fly stomodeal valve. Deletion of the stage-regulated HASP and SHERP genes on chromosome 23 of *Leishmania major* is known to stall metacyclogenesis in the sand fly but not in *in vitro* culture. Here, parasite mutants deficient in specific genes within the HASP/SHERP chromosomal region were used to investigate their role in metacyclogenesis, parasite transmission and establishment of infection. HASP/SHERP mutants stalled in metacyclogenesis *in vivo*, although still capable of osmotaxis, failed to secrete promastigote secretory gel, correlating with a lack of parasite accumulation in the thoracic midgut and failure to colonize the stomodeal valve. These defects prevent parasite transmission to a new mammalian host. Sand fly midgut homogenates modulate parasite behaviour *in vitro*, suggesting a role for molecular interactions between parasite and vector in *Leishmania* development within the sand fly. For the first time, stage-regulated expression of the small HASPA proteins has been demonstrated: HASPA2 is expressed only in extracellular promastigotes and HASPA1 only in intracellular amastigotes of *Leishmania*. Replacement of HASPA2 into the null locus background delays onset of pathology in BALB/c mice, despite its lack of expression in amastigotes, a phenotype associated with significantly slower onset and progression of pathology. This HASPA2-dependent effect is reversed by HASPA1 gene addition, suggesting that the HASPAs may be involved in host immunomodulation.

**A glance at what *Leishmania infantum chagasi* expresses inside *Lutzomyia longipalpis***

Erich Loza Telleria, Thais Lemos da Silva, João Ramalho Ortigão Farias, Yara Maria Traub-Csekö

Instituto Oswaldo Cruz-Fiocruz, Rio de Janeiro – RJ, Brasil

ytraub@ioc.fiocruz.br

*Leishmania infantum chagasi* is the causative agent and *Lutzomyia longipalpis* the main vector of visceral leishmaniasis. The parasite is acquired when a female sand fly feeds on an infected host. The ingested parasites must resist blood digestion and differentiate into promastigote forms to survive in the insect gut. In the early hours of blood digestion, intense proteolytic activity caused by the insect digestive enzymes occurs, as well as oxidative stress caused by the production of heme derived from hemoglobin digestion. After blood digestion and degradation of the perithrophic matrix the parasites migrate to the anterior part of the insect gut and differentiate into metacyclic promastigotes. During the whole cycle inside the insect, the parasites must survive the insect immune response and interaction with the gut microbiota. We are investigating which *L. i. chagasi* molecules are expressed during the establishment of infection inside the insect. Previous work has described *Leishmania* molecules that interact with the insect, such as lipophosphoglycans and proteophosphoglycans, but little is known regarding other genes expressed by *Leishmania* inside *Lu. longipalpis*. We analyzed the transcriptomes of *Lu. longipalpis* infected with *L. i. chagasi* promastigotes available in the Vector Base website. We identified *Leishmania* genes that are differentially expressed at 6 h, 24 h and 144 h post infection. At 6 h post infection we found an up-regulation of *Leishmania* amino acid permease and putative glycerol uptake protein genes related to amino acid metabolism and gluconeogenesis. At 24 h post infection, there was an increased expression of *Leishmania* HSP70, HSP60 and aldehyde dehydrogenase genes involved in stress responses and lipid metabolism. At 144 h, there was a major expression of *Leishmania* glucose transporter and amino acid transporter related to carbohydrate and amino acid metabolism. In order to validate this information we performed RTPCR using sand flies artificially infected with procyclic *L. i. chagasi* collected at 6 h, 24 h, 72 h and 96 h post infection, or procyclic and metacyclic-like parasites obtained from culture. The gene expression levels were calculated relative to a housekeeping gene (actin). For initial gene expression assays, we selected genes that were previously under investigation by our group. The expression level of FLAG/SMP1, which plays a role in the attachment of *Leishmania major* to the gut of *Phlebotomus papatasi*, and so far without a known function in *Lu. longipalpis*, was reduced at 24 h and 72 h, which related to the course of insect digestion, before returning to control levels at 96 h. In parasites from cultures, FLAG/SMP1 levels did not present significant variation. The promastigote surface antigen GP63, a zinc dependent metalloprotease, is notably increased in culture metacyclic-like parasites compared to procyclic parasites. This increase was also observed in *Leishmania*-infected insects that increased GP63 expression above control levels at 96 h post infection. We are currently investigating the expression of other genes that are differentially expressed by the parasite when infecting *Lu. longipalpis*. In conclusion, *Leishmania* present a plethora of differentially expressed genes that may favor the parasite survival in the constantly changing gut micro-environment.

Financial support: Fiocruz/PROEP; Faperj/APQ1; CNPq- Ciência sem Fronteiras- BJT.

***Lutzomyia longipalpis* TGF-β has a role in *Leishmania infantum chagasi* survival in the vector**

Tatiana Di-Blasi, E. Loza-Telleria, C. Marques, R. Macedo-Couto, M. Neves, A.J. Tempone, M. Ramalho-Ortigão, Yara Maria Traub-Csekö

Laboratório de Biologia Molecular de Parasitas e Vetores, IOC-FIOCRUZ, Rio de Janeiro, Brazil

tati.di.blasi@gmail.com

Leishmaniases are caused by *Leishmania* protozoans and transmitted to the vertebrate host mainly through bites of *Lutzomyia* (New World) or *Phlebotomus* (Old World) sand fly species. Following blood digestion, the ingested parasites adhere to the sand fly midgut epithelium, which is an important step to avoid parasite elimination together with digested blood remnants. This intimate relationship with the parasite leads to an immune response by the insect in the attempt to control infection. Previous studies from our group have shown that Caspar silencing in *Lutzomyia. longipalpis*, decreases the parasite load inside the vector, and that different bacterial challenges modulate the expression of a defensin. Other groups also showed that *Leishmania major* infection leads to an increase of defensin expression in *Phlebotomus duboscqi* and that high levels of ROS in *Lu. longipalpis* decrease the number of *L. mexicana* promastigotes 96 h after infection. We found that a *Lu. longipalpis* TGF-β (Transforming Growth Factor – β) was overexpressed 72 h post infection with *L. infantum chagasi*, when parasites were starting to attach to the midgut epithelium. In an attempt to test the effect of TGF-β depletion during the *Leishmania* infection, we fed *Lu. longipalpis* with *L. i. chagasi* and anti-TGF-β polyclonal antibody. We observed that the antibody-fed group had a significant increase in the number of parasites, in comparison to the control group without antibody, at the end of digestion. These results indicate that the *Leishmania* infection modulates TGF-β gene expression when the parasite contacts the insect digestive tract, and that TGF-β blocking at the beginning of the infection increases the parasite load. This indicates a possible role for TGF-β in controlling *L. i. chagasi* infection inside the sand fly. Previous studies with the malaria vector *Anopheles* showed that the modulation of a TGF-β expression could influence the production of NO and that this modulation affects the *Plasmodium* infection cycle. We have successfully silenced the TGF-β gene and are now investigating the effect of this silencing on the *L. i. chagasi* infection, iNOS gene expression and NO production.

**Novel method to quantify *Leishmania* metacyclic promastigotes delivered by individual sand fly bite reveals the efficiency of parasite transmission**

Émilie Giraud, Oihane Martin, Matthew Rogers

Faculty of Infectious and Tropical Diseases, London School of Hygiene and Tropical Medicine, WC1E 7HT, UK

matthew.rogers@lshtm.ac.uk

The composition of the infectious dose has never been directly determined from single infective bites. We developed a real-time PCR-based method for determining the number of *Leishmania mexicana* and *L. infantum* metacyclic promastigotes transmitted by individual *Lutzomyia longipalpis* sands flies to living mice. Despite high variation in the numbers of parasites delivered per bite, 76% of *L. mexicana* bites and 71% *L. infantum* bites contained ≥75% metacyclics. Analysis of multiple bites from individual infected flies revealed that approximately 60% could transmit ≥80% *L. mexicana* metacyclics for up to 10 consecutive bites. Meta-analysis of the 10th bite in relation to the vector’s midgut infection revealed that sand flies were more likely to repeatedly transmit a high proportion of metacyclics if the midgut was significantly blocked with parasites and a parasite-derived glycan-rich plug – the promastigote secretory gel. To test the impact of the proportion of metacyclics for infection, we mimicked “high-quality” (100% metacyclics) and “low-quality” (50% metacyclics), low dose *L. mexicana* transmissions to BALB/c mice using needles. Doses enriched for metacyclic promastigotes were associated with slower growing cutaneous lesions that harboured significantly more amastigotes compared to low quality infections and demonstrated greater transmission potential back to sand flies. This new method for interrogating sand fly infection and *Leishmania* transmission highlights the efficiency of parasite transmission and reveals the composition of the infectious dose as an important infection determinant.

**Blood feeding effect on *Phlebotomus papatasi* SP15 and SP44 salivary transcripts**

Nasibeh Hosseini-Vasoukolaei^1,2^, Amir Ahmad Akhavan^1^, Mahmood Jeddi-Tehrani^3^, Farah Idali^4^, Ali Khamesipour^5^, Mohammad Reza Yaghoobi-Ershadi^1^, Shaden Kamhawi^6^, Jesus G. Valenzuela^6^

^1^Department of Medical Entomology and Vector Control, School of Public Health, Tehran University of Medical Sciences, Tehran, Iran

^2^Department of Medical Entomology and Vector Control, Health Sciences Research Center, School of Public Health, Mazandaran University of Medical Sciences, Sari, Iran

^3^Monoclonal Antibody Research Center, Avicenna Research Institute, ACECR, Tehran, Iran

^4^Reproductive Immunology Research Center, Avicenna Research Institute, ACECR, Tehran, Iran

^5^Center for Research and Training in Skin Diseases and Leprosy, Tehran University of Medical Sciences, Tehran, Iran

^6^Laboratory of Malaria and Vector Research, National Institute of Allergy and Infectious Diseases, National Institute of Health, Rockville, MD 20852, USA

nasibeh.hoseini@gmail.com

As the components of an insect’s meal can induce hormonal changes in blood sucking insects and consequently gene expression, we verified whether or not blood feeding can modulate expression of *Phlebotomus papatasi* salivary gland transcripts. Phlebotomines were collected using aspirating tubes during 2012–2013 from Esfahan province, a hyperendemic area of zoonotic cutaneous leishmaniasis in central Iran. Sand flies were identified according to morphological characters using a valid systematic key. Females of *Ph. papatasi* were categorized into four groups according to physiological stages: blood fed, unfed, semi-gravid and gravid. In flies from each of these groups, the expression of PpSP15 and PpSP44 salivary transcripts were assessed using a Real-Time PCR method. Testing the effect of blood feeding on expression of PpSP15 in collected *Ph. papatasi* showed 2.41 ± 0.09 fold change in unfed vs. 4.44 ± 0.21 in fed group (*p* < 0.05). Semi-gravid flies expressed a significantly higher level of the PpSP15 transcript (2.74 ± 0.07) than gravid flies (1.17 ± 0.02) (*p* < 0.05). The transcript level of PpSP44 showed a 3.33 ± 0.25 fold increase in unfed vs. 4.58 ± 0.23 in the fed group (*p* < 0.05). Semi-gravid flies expressed a significantly higher level of the PpSP44 transcript (2.6 ± 0.05) than gravid flies (1.26 ± 0.02) (*p* < 0.05). The highest amount of PpSP15 and PpSP44 transcripts was observed in blood-fed sand flies and the lowest amount in gravid flies. This difference was highly significant (*p* < 0.01) using the Kruskal-Wallis statistical test. This induction of salivary transcripts following blood feeding of sand flies suggests that an important role is played by salivation during feeding. Higher gene expression in fed flies may be because of the subsequent need for regeneration of salivary proteins after a meal. Lower mRNA expression in unfed flies may suggest that sand fly has already deposited a sufficient amount of saliva that can be used during the feeding process, down regulating salivary gene transcription. The result of this study showed the correlation between sand fly feeding and the expression of salivary gene in wild-collected *Ph. papatasi*, thevector of ZCL in Iran.

***Phlebotomus orientalis* salivary proteins and antigens**

Iva Rohousova^1^, Alon Warburg^2^, Petr Volf^1^

^1^Department of Parasitology, Charles University in Prague, Prague, Czech Republic

^2^Department of Microbiology and Molecular Genetics, The Institute for Medical Research Israel-Canada, The Kuvin Centre for the Study of Infectious and Tropical Diseases, The Hebrew University – Hadassah Medical School, The Hebrew University of Jerusalem, Jerusalem, Israel

iva.rohousova@natur.cuni.cz

*Phlebotomus orientalis* is the most important vector of human visceral leishmaniasis in East Africa. As part of an international collaborative project dedicated to investigating the ecology and transmission dynamics of visceral leishmaniasis in Ethiopia, we had a unique opportunity to study salivary proteins of this sand fly species. Sand fly salivary proteins are an important part of the infective inoculum. They play a key role in the establishment of *Leishmania* infection. Furthermore, salivary proteins can be employed also in ELISA tests to measure exposure to sand fly bites. Some salivary molecules are highly antigenic and elicit a strong antibody response in repeatedly exposed hosts. This antibody response can be utilized as a marker of exposure to evaluate the effectiveness of vector control interventions, to estimate the risk of *Leishmania* transmission, or to indicate the feeding preferences of sand flies in search of *Leishmania* reservoir hosts. First we studied the transcriptome and proteome of *Ph. orientalis* female saliva. Thirteen main protein families were identified in the *Ph. orientalis* spitome, including enzymes and antigens known from saliva of other sand fly species such as apyrases, a hyaluronidase, yellow-related proteins, ParSP25-like proteins, D7-related proteins, and antigen 5-related proteins. All these *Ph. orientalis* proteins showed highest homology with their counterparts in saliva of *Ph. perniciosus*. Additionally, *Ph. orientalis* apyrases showed activity comparable to *Ph. perniciosus*, while hyaluronidase activity was the lowest among three *Larroussius* species tested. Further research was designed to characterize the main salivary antigens using sera of hosts repeatedly bitten by *Ph. orientalis*, either experimentally (murine model) or naturally (domestic animals from northwest Ethiopia). Individuals belonging to all animal species tested (cattle, dogs, sheep, goats, donkeys) possessed anti-*Ph. orientalis* saliva IgG antibodies, indicating their possible involvement in the transmission of *Leishmania donovani* as sources of blood for vector sand flies. The most intensive reactions with murine and canine sera were detected with the yellow-related proteins, apyrases, D7-related proteins, and antigen 5-related proteins. The five most auspicious antigens were bacterially-expressed to determine the possibility of replacing salivary gland homogenate in ELISA tests, which would enable broader use of this test independent of sand fly colony maintenance. Out of these five recombinant proteins, yellow-related protein rPorSP24 showed the most promising features; it achieved high correlation with salivary gland homogenate (*ρ* = 0.8) and the highest values of specificity, sensitivity, positive and negative predictive values in ELISA tests with sera of dogs and sheep naturally-exposed to *Ph. orientalis*. Finally, we selected and tested B-cell epitopes from antigenic salivary proteins to further simplify the serology screening. Two peptides from a yellow-related protein (PorSP24) and one peptide from a ParSP25-like protein (PorSP65) are hot candidate antigens to measure anti-*Ph. orientalis* saliva antibodies in canine sera. Such an approach may increase the specificity and sensitivity of the reaction and could enable large-scale epidemiological studies.

Financial support: Bill and Melinda Gates Foundation (OPPGH5336); Czech Science Foundation (13-05292S).

**Parity/nulliparity and sand fly salivary gland-gene expression**

Nasibeh Hosseini-Vasoukolaei^1,2^, Amir Ahmad Akhavan^1^, Mahmood Jeddi-Tehrani^3^, Farah Idali^4^, Ali Khamesipour^5^, Mohammad Reza Yaghoobi-Ershadi^1^, Shaden Kamhawi^6^, Jesus G. Valenzuela^6^

^1^Department of Medical Entomology and Vector Control, School of Public Health, Tehran University of Medical Sciences, Tehran, Iran

^2^Department of Medical Entomology and Vector Control, Health Sciences Research Center, School of Public Health, Mazandaran University of Medical Sciences, Sari, Iran

^3^Monoclonal Antibody Research Center, Avicenna Research Institute, ACECR, Tehran, Iran

^4^Reproductive Immunology Research Center, Avicenna Research Institute, ACECR, Tehran, Iran

^5^Center for Research and Training in Skin Diseases and Leprosy, Tehran University of Medical Sciences, Tehran, Iran

^6^Laboratory of Malaria and Vector Research, National Institute of Allergy and Infectious Diseases, National Institute of Health, Rockville, MD 20852, USA

nasibeh.hoseini@gmail.com

In the current study we evaluated the expression of two salivary gland genes, PpSP15 and PpSP44, in both parous and nulliparous wild-collected sand flies. As nulliparous flies have never oviposited, parity is usually used to determine the age structure of a population. This study was carried out in Esfahan province, a hyperendemic area of zoonotic cutaneous leishmaniasis in central Iran. Sand flies were collected using aspirating tubes and were identified according to morphological characters. Females of *Phlebotomus papatasi* were dissected and categorized into two groups, parous and nulliparous, according to the status of the accessory glands. The expression of PpSP15 and PpSP44 salivary transcripts were assessed in the two groups using Real-Time PCR. The fold changes for parous and nulliparous groups of sand flies, respectively, were 2.46 ± 0.14 and 2.04 ± 0.12 for PpSP15 expression and 1.29 ± 0.07 and 1.35 ± 0.11 fold for PpSP44 expression. In this study of salivary gland-gene expression in *Ph. papatasi*, only SP15 appeared to be consistently influenced by parity. In contrast, the modulation of the SP44 expression profile was not statistically significant between parous and nulliparous flies. A higher expression level of SP15 transcript was observed in the parous group of flies, which are older compared to nulliparous flies. This study showed that the status of accessory glands in sand flies is another physiological factor influencing salivary gene expression profiles.

**Different approaches for further application of MALDI-TOF mass spectrometry for species identification of phlebotomine sand flies**

Kristýna Hlavackova^1^, Vit Dvorak^1^, Petr Halada^2^, Petr Volf^1^

^1^Department of Parasitology, Charles University, Prague, Czech Republic

^2^Institute of Microbiology, Academy of Science, Prague, Czech Republic

hlavackova.k@centrum.cz

Traditional morphological approaches to identification of sand fly species rely on minute characters, which can be difficult to access, especially in females. Moreover, there is no morphological approach available that would readily enable the identification of immature stages (larvae, pupae). Nevertheless, conclusive species identification in endemic areas, where morphologically similar species with different vectorial capacity and ecology may occur, is critical, emphasizing the importance of developing alternative molecular methods of species identification. Protein profiling by matrix-assisted laser desorption/ionization time-of-flight (MALDI-TOF) mass spectrometry is a promising technique for identification of various organisms including insects. It is simple, accurate, and requires minimal sample preparation. Extraction of peptides and small proteins is mixed with a suitable aromatic acid serving as a MALDI matrix. After co-crystallization, the crystals are irradiated, desorbed and ionized by laser pulses to generate gaseous peptide/protein ions, which are then measured by mass spectrometer. The obtained mass spectrum represents a characteristic and unique protein fingerprint, which allows rapid and effective species identification. We optimized methods of specimen capture, storage, sample preparation, tested suitability of different body parts of sand fly for protein identification, and developed a multi-approach protocol that utilizes a single insect body for several identification methods: morphology-, DNA- and protein-based. In sand fly females, we studied the influence of blood meal and egg development at several time intervals after feeding on the quality of protein profile. Moreover, we tested the possibility of identifying blood-meal origin; sand fly females experimentally fed on different vertebrate hosts were analyzed at various points in time and protein profiles of abdomen containing host blood were compared with protein profiles obtained from host blood only. In larvae (L2 to L4) and pupae of different stages of development, protein spectra were acquired and compared with profiles of adult sand flies. Impact of larval diet on the quality of the protein profile was investigated. In summary, MALDI-TOF protein profiling is a suitable, time- and cost-effective method for species identification of sand flies including large sets of field-caught sand flies, if these are collected, stored and analyzed using a standard, optimized protocol.

Financial support: This project is supported by the Czech Science Foundation (GACR 15-04329S) and by VectorNet.

**MALDI-TOF protein profiling as a method of choice for high-throughput species identification of sand flies – an example from the Balkan**

Vit Dvorak^1^, Kristýna Hlavackova^1^, Petr Halada^2^, Bulent Alten^3^, Vladimir Ivovic^4^, J. Omeragic^5^, I. Pajovic^6^, F. Martinkovic^7^, O. Mikov^8^, J. Stefanovska^9^, Petr Volf^1^

^1^Department of Parasitology, Charles University, Prague, Czech Republic

^2^Institute of Microbiology, Academy of Science, Prague, Czech Republic

^3^Department of Biology, Hacettepe University, Ankara, Turkey

^4^Department of Biodiversity, University of Primorska, Koper, Slovenia

^5^Department of Parasitology, University of Sarajevo, Sarajevo, Bosnia and Herzegovina

^6^Center of Plant Protection, University of Montenegro, Podgorica, Montenegro

^7^Department for Parasitology and Parasitic Diseases, University of Zagreb, Zagreb, Croatia

^8^Parasitology and Tropical Medicine, National Centre of Infectious and Parasitic Diseases, Sofia, Bulgaria

^9^Faculty of Veterinary Medicine, Ss. Cyril and Methodius University, Skopje, Macedonia

vidvorak@natur.cuni.cz

Conclusive species identification of sand flies by morphological analysis remains a challenging task due to limited availability of robust characters, their intraspecific variability among different populations and a need of expertise to assess them. According to proposed scenarios of future climatic trends, sand flies may emerge in regions with a lack of expertise for their species identification by classical morphological approach. Therefore, especially for high-throughput assessment of field surveys in endemic areas, there is a need for complementary methods of species identification relying on molecular techniques. Protein profiling by matrix-assisted laser desorption/ionization time-of-flight (MALDI-TOF) mass spectrometry emerged as a molecular method recently used for species identification of various medically important arthropod vectors including phlebotomine sand flies. With a standardized protocol of specimen capture, storage and sample preparation and a robust reference database, it provides characteristic and unique protein fingerprints that allow conclusive species identification. It requires minimal sample preparation that enables utilization of a single sand fly specimen for several purposes. Balkan countries are in a transition area in the European part of the Mediterranean basin where sand fly fauna of the western part meets with eastern elements and where several species have the limit of their geographical distribution. At the same time, data on sand fly presence and species composition are scarce due to recent upheavals. Due to two field surveys in the Balkan area and adjacent countries (Bosnia and Hercegovina, Bulgaria, Croatia, Hungary, Macedonia, Montenegro, Serbia and Slovenia) in summer of 2015, approx. 700 sand fly specimens of a total of 8000 captured were analyzed by protein profiling. The sand flies were trapped by CDC traps placed in domestic, peridomestic and sylvatic sites and processed according to standardized protocol. An in-house reference spectrum database was used as reference comprising 16 sand fly species based on specimens from both laboratory colonies as well as field-collected specimens whose species identity was conclusively confirmed by morphological analysis and DNA sequencing. Analyzed specimens belonged to species of four subgenera of the genus *Phlebotomus*: *Adlerius* (*Ph. balcanicus* and *Ph. simici*), *Larroussius* (*Ph. neglectus*, *Ph. perfiliewi*, *Ph. tobbi*), *Paraphlebotomus* (*Ph. sergenti*) and *Phlebotomus* (*Ph. papatasi*). Moreover, two species of the genus *Sergentomyia* were also recorded (*Se. minuta* and *Se. dentata*). The method proved very suitable for identification of female specimens of the subgenus *Larroussius* (492 analyzed specimens) where species-specific diagnostic markers were characterized beside the overall protein spectra. All dubious specimens where morphological and protein-based identification did not agree were further characterized by a sequencing analysis (cytochrome oxidase 1 gene) which confirmed the identification by MALDI-TOF MS in all cases. MALDI-TOF protein profiling proved to be a time- and cost-effective method of choice for species identification of large sets of field-caught sand flies if these are collected, stored and analyzed using a standard, optimized protocol.

Financial support: This project is supported by the Czech Science Foundation (GA15-04329S) and by VectorNet.

**New generation sequencing (NGS) as a tool for identification of pooled sand flies**

Nazli Ayhan^1,2^, Vit Dvorak^3^, Cigdem Alkan^1,2^, Petr Volf^3^, Rémi N. Charrel^1,2^

^1^UMR “Émergence des Pathologies Virales” (EPV: Aix-Marseille Universite – IRD 190 – Inserm 1207 – EHESP), Marseille, France

^2^Institut hospitalo-universitaire Méditerranée infection, APHM Public Hospitals of Marseille, Marseille, France

^3^Department of Parasitology, Charles University, Prague, Czech Republic

remi.charrel@univ-amu.fr

The recent renewed interest in viruses transmitted by sand flies has raised technical questions which should not be neglected to achieve multidisicplinary project and to share the entomological material collected from field work in a manner that can be beneficial for all bodies involved in the project. Identification of sand flies at the species level remains a difficult task and should be conducted at the individual level which is time and labor-consuming regardless the technique used (morphologic, sequencing, mass spectrometry). Manipulations of sand flies lead to a degradation of live viruses and of their RNA; therefore, the requirements of virologists is to keep the insects “*as untouched as possible*”. When a virus is isolated, and sequenced, the next question is “what is the vector?”. Frequently facing this paradoxical situation, we developed a method that allows to identify the different species of sand flies contained in a pool. This method takes advantage (i) of PCR using generic primers to amplify sequences that can discriminate the species of sand flies, (ii) of NGS capability to generate large numbers of reads that can be statistically analysed. This technique has been initially used “experimentally” from field-collected pools of sandflies. In view of the promising results, we have decided to evaluate this method in a more “scientific” and “systematic” manner based on previously identified sandflies. The results of both stages will be presented and discussed. This method cannot replace gold standard techniques for identification; however, it may provide direct information concerning the species of sand flies acting as vectors of viruses.

